# Characteristics of *Staphylococcus epidermidis* Isolated from Humans and Animals

**DOI:** 10.3390/ijms27198585

**Published:** 2026-09-25

**Authors:** Paulina Prorok, Magdalena Siedlecka, Magdalena Karwańska, Milena Skrok, Marta Miszczak, Maciej Wernecki, Marta Książczyk, Krzysztof Rypuła, Karolina Bierowiec

**Affiliations:** 1 Division of Infectious Diseases and Veterinary Administration, Department of Epizootiology with Exotic Animal and Bird Clinic, Faculty of Veterinary Medicine, Wroclaw University of Environmental and Life Sciences, 50-366 Wroclaw, Poland; 2 Department of Microbiology, Faculty of Biological Sciences, University of Wroclaw, 51-148 Wroclaw, Poland

**Keywords:** One Health, *Staphylococcus epidermidis*, CoNS, antimicrobial resistance

## Abstract

*Staphylococcus epidermidis* is an important opportunistic pathogen and a potential reservoir of antimicrobial resistance determinants, yet comparatively little is known about isolates originating from both humans and companion animals. This study characterized 320 *S. epidermidis* isolates obtained from humans, cats, and dogs within a One Health framework using phenotypic, molecular, proteomic, and genomic approaches. Antimicrobial susceptibility testing revealed high frequencies of resistance to oxacillin (67.19%), erythromycin (66.56%), tetracycline (55.63%), and clindamycin (53.75%) by disk diffusion. PCR-based analysis identified *blaZ* as the most prevalent resistance determinant (50.63%), whereas whole-genome sequencing of a selected subset revealed a broader and heterogeneous repertoire of resistance-associated genes. Among 67 sequenced isolates, 26 known sequence types were identified, including several STs occurring in isolates from more than one host species. Core-genome phylogenetic analysis demonstrated substantial genomic diversity without complete segregation according to host species or clinical status. Genotype–phenotype concordance was generally limited and gene dependent, and discrepancies were also observed between PCR- and WGS-based detection for selected resistance determinants. Biofilm formation was significantly influenced by incubation temperature, host species, and clinical status, while growth kinetics showed pronounced strain-specific variability and no consistent relationship with biofilm-forming capacity. MALDI-TOF MS profiling and partial *rpoB* phylogeny likewise showed considerable overlap among isolates from different host groups. In the *Galleria mellonella* infection model, mortality was inoculum dependent and varied substantially among strains. Overall, the findings demonstrate marked phenotypic and genomic heterogeneity among *S. epidermidis* isolated from humans and companion animals and indicate that some clonal backgrounds and resistance determinants occur across host species, highlighting the relevance of this species within a One Health context.

## 1. Introduction

Among coagulase-negative staphylococci (CoNS), *Staphylococcus epidermidis* (*S. epidermidis*) is considered one of the most clinically important species. Among all CoNS species, it is the most frequently isolated cause of healthcare-associated infections (HAIs) [1,2]. For many years, this bacterium was regarded as a harmless skin commensal or merely a contaminant of clinical specimens; however, it is now recognized as one of the most important opportunistic pathogens associated with healthcare settings [1,2,3,4]. The transition from commensalism to pathogenicity is primarily associated with biomaterial colonization, biofilm formation, adaptation to the hospital environment, and the acquisition of antimicrobial resistance determinants [1,3]. In some studies, *S. epidermidis* accounted for more than 50% of all CoNS isolates recovered from blood cultures, making it the most frequently isolated member of this group from clinical specimens [2,5].

The natural reservoir of *S. epidermidis* is primarily the skin and mucosal surfaces of humans, where it represents one of the most abundant members of the skin microbiota and is present in the vast majority of healthy individuals. In sebaceous gland-rich body sites, its density may reach up to 10^5^ colony-forming units per square centimeter (CFU/cm^2^), making it one of the most frequently isolated staphylococcal species colonizing these skin regions [1,6]. It most commonly colonizes the skin, anterior nares, and oral cavity [1,2]. Beyond humans, this bacterium has also been isolated from companion animals, including dogs and cats. Studies conducted in Poland demonstrated that *S. epidermidis* is among the most frequently isolated CoNS species in healthy cats [7]. In dogs, it is regularly isolated as a component of the skin and mucosal microbiota, although it is generally less prevalent than *Staphylococcus pseudintermedius* [2]. In companion animals, it has also been reported as an opportunistic pathogen associated with skin infections, wounds, external ear canal infections, and implant-associated infections [2,7].

Despite exhibiting substantially lower virulence than *Staphylococcus aureus*, *S. epidermidis* represents a leading cause of infections associated with biomaterials and medical devices, being the most frequently identified CoNS species involved in prosthetic joint infections (PJIs) and catheter-related bloodstream infections (CRBSIs) [3,8,9]. The species plays a particularly important role in infections involving prosthetic heart valves, vascular catheters, and other implanted medical devices. Moreover, it is recognized as a dominant etiological agent of late-onset neonatal sepsis (LOS), accounting for approximately 30–40% of such infections in preterm infants and very-low-birth-weight (VLBW) neonates admitted to neonatal intensive care units [1,3,10]. Infections caused by this species are associated with prolonged hospitalization, increased patient morbidity, and substantial healthcare costs [4,10].

Biofilm formation is considered the most important virulence factor of *S. epidermidis* and largely determines its success as an opportunistic pathogen [3,11]. This structure facilitates adherence to both biotic and abiotic surfaces, limits antimicrobial penetration, and protects bacterial cells from host immune defenses [11,12]. In contrast to *S. aureus*, whose pathogenicity largely depends on the production of numerous toxins and enzymes, *S. epidermidis* compensates for its lower virulence through an exceptional ability to form biofilms, adapt to hospital environments, and acquire resistance genes [1,3,4]. In addition to biofilm formation, the pathogenicity of this species is associated with numerous surface adhesins (including SdrG/Fbe, AtlE, and Embp), phenol-soluble modulins (PSMs), extracellular enzymes, and mechanisms that facilitate evasion of the host immune response [3,4].

In recent years, increasing attention has been paid to the genomic plasticity of *S. epidermidis*. Studies have demonstrated the existence of specialized hospital-adapted lineages characterized by enhanced virulence and multidrug resistance (MDR). Of particular epidemiological importance is the global dissemination of multidrug-resistant clones, especially those belonging to sequence type ST2, which predominates among hospital-associated *S. epidermidis* isolates across multiple continents [13,14]. Furthermore, *S. epidermidis* is recognized as an important reservoir of mobile genetic elements (MGEs), including the staphylococcal cassette chromosome *mec* (SCC*mec*), which mediates methicillin resistance [13,15]. Of particular concern is the increasing prevalence of methicillin-resistant *S. epidermidis* (MRSE) strains, which frequently exhibit resistance to multiple additional classes of antimicrobial agents [2,4,13]. Genetic exchange between *S. epidermidis* and other staphylococcal species, including *S. aureus*, may contribute to the dissemination of resistance determinants and the emergence of novel epidemic clones [2,15].

From a One Health perspective, the potential transmission of strains and resistance genes between humans and companion animals is of particular interest. The presence of *S. epidermidis* in both humans and animals, together with the genetic relatedness observed among some isolates, suggests that this species may play a role in the exchange of antimicrobial resistance determinants between different hosts [2,7]. Although *S. epidermidis* is one of the best-characterized CoNS species in humans, knowledge regarding animal-derived isolates remains limited. This is particularly true for comparative studies simultaneously investigating isolates from humans, dogs, and cats, including their pathogenic potential, biofilm-forming capacity, antimicrobial resistance profiles, and population diversity.

The aim of the present study was to characterize *Staphylococcus epidermidis* isolates obtained from humans, dogs, and cats within a One Health framework. Antimicrobial resistance profiles, biofilm-forming ability under different temperature conditions, growth dynamics, proteomic diversity, and whole-genome characteristics were evaluated to identify potential host- and clinical-status-associated differences. Whole-genome sequencing was further used to investigate sequence type diversity, antimicrobial resistance determinants, and phylogenomic relationships among the isolates, thereby providing a comprehensive assessment of the epidemiology, population structure, and pathogenic potential of *S. epidermidis* across human and companion-animal hosts.

## 2. Results

### 2.1. Species Identification and Phylogenetic Analysis

Species identity was confirmed for all 320 archived isolates using MALDI-TOF MS and *S. epidermidis*-specific *gseA* PCR. Additional *rpoB*-based molecular identification of the 67 isolates selected for genomic analysis was concordant with their identification as *S. epidermidis*.

Principal component analysis (PCA) of the MALDI-TOF MS spectral profiles revealed substantial overlap among *S. epidermidis* isolates originating from different hosts and representing different clinical statuses, with no clear separation of the individual groups. Hierarchical clustering of the 320 study isolates and one *S. epidermidis* reference strain (*n* = 321 spectral profiles in total) identified seven clusters: (1) blue (*n* = 20), (2) dark green (*n* = 125), (3) light green (*n* = 20), (4) purple (*n* = 4), (5) yellow (*n* = 102), (6) pink (*n* = 2), and (7) red (*n* = 48).

The distribution of isolates within the clusters was heterogeneous. The largest, dark-green cluster was strongly dominated by human-derived isolates, which accounted for 119 of 125 profiles (95.2%), including 109 isolates from healthy individuals and 10 from clinically affected individuals. The yellow cluster contained the largest absolute number of isolates obtained from clinically affected hosts among all identified clusters. Of the 101 study isolates assigned to this cluster, 12 originated from clinically affected hosts, including 8 human, 2 feline, and 2 canine isolates. The remaining isolates comprised 57 isolates from healthy humans, 22 from healthy cats, and 10 from healthy dogs; the *S. epidermidis* reference strain constituted the 102nd profile assigned to this cluster.

Isolates from healthy humans were most frequently assigned to the dark-green cluster (109/243; 44.9%), followed by the yellow (57/243; 23.5%) and red clusters (38/243; 15.6%). Isolates from clinically affected humans were most frequently represented in the dark-green cluster (10/21; 47.6%), followed by the yellow cluster (8/21; 38.1%). Among animal-derived isolates, the yellow cluster contained 22/32 (68.8%) isolates from healthy cats, both isolates from clinically affected cats (2/2; 100%), 10/18 (55.6%) isolates from healthy dogs, and 2/4 (50.0%) isolates from clinically affected dogs. Despite these differences in relative distribution, isolates from healthy and clinically affected hosts were distributed across the same major clusters, and no distinct host- or clinical-status-specific clustering pattern was evident.

To provide a clearer visualization of the relationships among isolates obtained from clinically affected hosts, these isolates were analysed separately together with the *S. epidermidis* reference strain in Figure 1. The complete PCA and hierarchical clustering analysis comprising all 320 study isolates together with the reference strain is provided in Supplementary Figure S1 and Table S1.

Phylogenetic analysis based on partial *rpoB* gene sequences confirmed the assignment of all analysed isolates to *S. epidermidis*. The isolates, together with the reference strain *S. epidermidis* CP066303.1, formed a clearly separated clade distinct from the other analysed *Staphylococcus* species, with bootstrap support of 100%. Some phylogenetic heterogeneity and several internal subgroups were observed within the *S. epidermidis* clade. One of the more distinct subgroups comprised isolates hCa3, hCa13, hCa14, and hCa8 together with hFe19 and was supported by a bootstrap value of 92%. This pattern may indicate local similarity among some isolates originating from the same host group; however, no complete phylogenetic segregation according to host origin or clinical status was observed, as isolates representing the respective groups were also distributed across other parts of the *S. epidermidis* clade. A detailed phylogenetic tree is presented in Figure 2.

### 2.2. Comparative Whole-Genome Analysis

#### 2.2.1. Whole-Genome Sequencing Quality Assessment

Quality assessment was performed for all 67 *S. epidermidis* genome assemblies. Assemblies were evaluated based on total genome length, estimated completeness, contamination, and number of contigs. Numerical acceptance criteria for downstream WGS-based antimicrobial resistance and concordance analyses were defined as a total genome length of 2.2–2.8 Mb, completeness ≥ 95%, contamination ≤ 5%, and fewer than 200 contigs.

Fifty-five of 67 genomes (82.09%) met the predefined numerical criteria and were retained for downstream WGS-based antimicrobial resistance and genotype–phenotype concordance analyses. Of these, 54 assemblies additionally fulfilled the high-quality criteria used for pangenome reconstruction and construction of the core-genome phylogenetic backbone. The remaining assembly, sHo18, met the acceptance criteria for WGS-based antimicrobial resistance analyses but did not meet the more stringent criteria for inclusion in the high-quality phylogenetic backbone and was therefore subsequently incorporated by phylogenetic placement.

Eleven additional assemblies did not meet the acceptance criteria for WGS-based antimicrobial resistance analyses but fulfilled the predefined criteria for lower-quality phylogenetic placement. Together with sHo18, these 12 assemblies were placed onto the 54-genome high-quality backbone, resulting in a final phylogeny comprising 66 isolates. One assembly, hFe23, did not meet the minimum completeness criterion for phylogenetic placement and was excluded from the final phylogenetic analysis.

Failure to meet the WGS-based antimicrobial resistance criteria was predominantly associated with reduced genome completeness, frequently accompanied by assembly lengths below 2.2 Mb. None of the excluded genomes exceeded the contamination threshold of 5% or the maximum contig count of 200. Detailed assembly quality metrics for all isolates are provided in Supplementary Table S3.

#### 2.2.2. Multilocus Sequence Typing

Multilocus sequence typing (MLST) was performed using WGS data from 67 *S. epidermidis* isolates. Complete seven-locus MLST profiles were obtained for 52/67 isolates (77.61%). Of these, 49/67 isolates (73.13%) had complete and unambiguous profiles corresponding to sequence types represented in the PubMLST database at the time of final verification, comprising 26 distinct sequence types (STs), whereas three isolates (4.48%) had complete allelic profiles that did not correspond to any ST available in the database and were therefore retained as unassigned profiles. Six isolates (8.96%) showed ambiguous allele assignments, whereas nine isolates (13.43%) had one or more missing MLST loci and could not therefore be reliably assigned to an ST.

Among the isolates assigned to known STs, ST153 was the most frequent sequence type, occurring in seven isolates, followed by ST87 in four isolates. ST297, ST19, ST210, ST190, and ST5 were each represented by three isolates, whereas ST32, ST35, ST81, and ST88 occurred in two isolates each. The remaining known STs were represented by single isolates.

Several sequence types occurred in isolates originating from more than one host species. ST153 and ST87 were identified among isolates from humans, cats, and dogs. ST19, ST32, ST35, and ST88 were shared between human and feline isolates, whereas ST190 and ST81 occurred among human and canine isolates. ST5 was detected among feline and canine isolates. In contrast, ST297 was detected only among human isolates and ST210 only among feline isolates within the analysed WGS subset. The occurrence of identical STs in different host species was interpreted as evidence of shared clonal lineages rather than direct evidence of interspecies transmission. Detailed MLST results, including seven-locus allelic profiles, assignment status, host origin, clinical status, and sampling site, are provided in Supplementary Table S4.

#### 2.2.3. Pangenome Structure and Core-Genome Phylogeny

Pangenome reconstruction was performed using 54 high-quality *S. epidermidis* genome assemblies. A total of 4711 gene clusters were identified across the analysed genomes. Using a threshold of presence in ≥95% of isolates, 1973 gene clusters were assigned to the core-genome component, comprising 1927 genes present in ≥99% of genomes and 46 soft-core genes present in 95–99% of genomes. The remaining 2738 gene clusters constituted the accessory genome, including 502 shell genes and 2236 cloud genes.

Maximum-likelihood phylogenetic analysis based on the concatenated core-gene alignment demonstrated substantial genetic diversity within the analysed *S. epidermidis* collection. Human-, feline-, and canine-derived isolates were distributed throughout the phylogeny, and no complete phylogenetic segregation according to host species or clinical status was observed. Several closely related groups contained isolates originating from different host species and, in some cases, from both clinically healthy and clinically affected hosts.

Core-genome relationships were broadly concordant with MLST assignments for several recurrent sequence types. All seven ST153 isolates formed a closely related phylogenetic group comprising isolates of human, feline, and canine origin. The ST297 isolates also clustered closely within the phylogeny, as did the ST210 isolates. Close phylogenetic relationships were additionally observed among several other recurrent sequence types. However, seven-locus MLST did not completely reproduce the relationships resolved by core-genome analysis, illustrating the greater discriminatory resolution provided by genome-wide sequence comparison.

The maximum-likelihood backbone phylogeny was reconstructed using the 54 high-quality genome assemblies. Lower-quality assemblies fulfilling the predefined placement criteria were subsequently incorporated into the phylogenetic framework using phylogenetic placement. The resulting final tree comprised 66 isolates (Figure 3). Most major internal branches were strongly supported, and the scale bar represents 0.005 substitutions per site.

#### 2.2.4. Antimicrobial Resistance Determinants Identified by WGS

Analysis of the 55 genome assemblies retained for WGS-based antimicrobial resistance analysis identified multiple acquired antimicrobial resistance determinants. The most frequently detected were *blaZ* (36/55; 65.45%), *msrA* (23/55; 41.82%), *mphC* (19/55; 34.55%), *mecA* (17/55; 30.91%), *tet(K)* (14/55; 25.45%), *ermC* (11/55; 20.00%), and *aac(6′)-Ie-aph(2″)-Ia* (11/55; 20.00%). *ANT(4′)-Ia* was detected in 9/55 isolates (16.36%), whereas *vgaA* was present in 9/55 (16.36%) and *mupA* in 7/55 (12.73%). *fusB* was identified in 5/55 isolates (9.09%), whereas *lnuA* was detected in 4/55 isolates (7.27%). Additional acquired resistance determinants occurred at lower frequencies.

Several resistance-associated genes encoding multidrug efflux systems or regulatory components were highly prevalent or ubiquitous within the analysed collection, including *mgrA*, *norA*, *LmrS*, *sepA*, *sdrM*, and *arlR/arlS*. These determinants were considered separately from acquired antimicrobial resistance genes because they represent broadly distributed intrinsic or resistance-associated functions. Genes associated with reduced susceptibility to antiseptic compounds, including *qacA* and *qacH*, were also identified. Detailed CARD results are provided in Supplementary Table S6.

#### 2.2.5. Distribution of WGS-Detected Antimicrobial Resistance Determinants According to Host Origin and Clinical Status

The distribution of acquired antimicrobial resistance determinants was compared according to host species and clinical status among the 55 genomes retained for WGS-based antimicrobial resistance analysis. Although numerical differences were observed for several genes, none remained statistically significant after correction for multiple comparisons using the Benjamini–Hochberg false discovery rate procedure.

Before correction, the distribution of *mphC* (*p* = 0.036), *lnuA* (*p* = 0.009), and *dfrG* (*p* = 0.037) differed among host species; however, these associations were no longer significant after FDR adjustment (*q* = 0.211, 0.158, and 0.211, respectively). No acquired resistance determinant showed a significant association with clinical status, either before or after FDR correction. Overall, no statistically supported host- or clinical-status-specific distribution pattern was identified within the analysed WGS subset.

#### 2.2.6. Genotype–Phenotype Concordance Based on WGS

Genotype–phenotype concordance was assessed among the 55 isolates retained for WGS-based antimicrobial resistance analysis by comparing WGS-detected antimicrobial resistance determinants with phenotypic susceptibility classifications obtained by disk diffusion and MIC testing. Both phenotypic methods were analysed independently.

Overall agreement was limited and varied among antimicrobial classes. For oxacillin, *mecA* showed essentially no agreement with disk-diffusion classification (Cohen’s κ = −0.008) and only slight agreement with MIC-based classification (κ = 0.101), with significant discordance predominantly directed towards phenotype-resistant/genotype-negative isolates (McNemar *p* < 0.001 for both methods). For erythromycin, the combined presence of *ermA*, *ermC*, *msrA*, or *mphC* showed the highest agreement among the MIC-based comparisons (κ = 0.221), although significant phenotype-resistant/genotype-negative discordance remained. Clindamycin and tetracycline showed low agreement, with discordance again predominantly directed towards phenotype-resistant/genotype-negative isolates. Among the additional disk-diffusion comparisons, *mupA* showed moderate agreement with mupirocin resistance (κ = 0.411), whereas *aac(6′)-Ie-aph(2″)-Ia* showed fair agreement with gentamicin resistance (κ = 0.375). Detailed genotype–phenotype concordance results, including concordant and discordant categories, Cohen’s κ values, McNemar test results, and the direction of discordance, are provided in Supplementary Table S7.

#### 2.2.7. Concordance Between PCR- and WGS-Based Detection of Antimicrobial Resistance Genes

The concordance between PCR- and WGS-based detection of antimicrobial resistance genes was assessed for the 55 *S. epidermidis* isolates retained in the WGS antimicrobial resistance dataset. Agreement varied considerably depending on the analysed resistance determinant.

For *blaZ*, the gene was detected by PCR in 39/55 isolates (70.9%), whereas WGS identified *blaZ* in 36/55 isolates (65.5%). Concordant positive results were obtained for 32 isolates, whereas seven isolates were PCR-positive/WGS-negative and four were PCR-negative/WGS-positive. The remaining 12 isolates were negative by both methods. Overall agreement was 80.0%, corresponding to moderate agreement beyond chance (Cohen’s κ = 0.541). No significant directional asymmetry was observed between the discordant categories (McNemar *p* = 0.549).

For *mecA*, PCR detected the gene in 13/55 isolates (23.6%), compared with 17/55 isolates (30.9%) by WGS. All 13 PCR-positive isolates were also positive by WGS, whereas four isolates were PCR-negative/WGS-positive and 38 isolates were negative by both methods. Overall agreement was 92.7%, corresponding to almost perfect agreement beyond chance (κ = 0.818), with no statistically significant asymmetry between discordant categories (McNemar *p* = 0.125).

The highest concordance was observed for *aac(6′)-Ie-aph(2″)-Ia*, for which 11 isolates were positive by both methods and no discordant results were observed. The remaining 44 isolates were negative by both methods. Overall agreement was 100.0% (κ = 1.000; McNemar *p* = 1.000).

For *ermC*, overall agreement was 89.1% (κ = 0.571); five isolates were positive by both methods, whereas six additional isolates were detected by WGS but not by PCR (McNemar *p* = 0.031). Agreement was 90.9% for *ermA*, although no concordant positive isolates were observed and the corresponding κ value was negative (κ = −0.046), reflecting the very low prevalence of positive results.

Agreement was lower for *tetK*, *fusB*, and *mupA*. For *tetK*, five isolates were positive by both methods, eight were PCR-positive/WGS-negative, nine were PCR-negative/WGS-positive, and 33 were negative by both methods, resulting in an overall agreement of 69.1% (κ = 0.166; McNemar *p* = 1.000). For *fusB*, overall agreement was 85.5% (κ = 0.120), whereas for *mupA* agreement was also 85.5% (κ = 0.134). These low κ values reflected the limited concordance among positive detections despite relatively high overall agreement.

Overall, PCR–WGS concordance was strongly gene dependent, ranging from complete agreement for *aac(6′)-Ie-aph(2″)-Ia* and almost perfect agreement for *mecA* to substantially lower agreement for *tetK*, *fusB*, and *mupA*. Detailed gene-specific PCR–WGS concordance data, including concordant and discordant categories, Cohen’s κ values, and McNemar test results, are presented in Supplementary Table S8.

### 2.3. Phenotypic Antimicrobial Susceptibility

#### 2.3.1. Antimicrobial Susceptibility Testing by Disk Diffusion

The highest overall frequencies of resistance were observed for oxacillin (215/320; 67.19%), erythromycin (213/320; 66.56%), tetracycline (178/320; 55.63%), and clindamycin (172/320; 53.75%). Resistance to penicillin G was detected in 127/320 isolates (39.69%), whereas resistance to ampicillin and amoxicillin/clavulanic acid was observed in 84/320 (26.25%) and 36/320 (11.25%) isolates, respectively. Resistance to ciprofloxacin was less frequent (16/320; 5.00%), followed by mupirocin (14/320; 4.38%), gentamicin (6/320; 1.88%), fusidic acid and marbofloxacin (5/320; 1.56% each), chloramphenicol (4/320; 1.25%), and trimethoprim/sulfamethoxazole (1/320; 0.31%). No isolates fulfilled the applied resistance criteria for tobramycin, rifampin, tigecycline, or linezolid.

Among the larger study groups, oxacillin resistance was most frequent among isolates from clinically healthy humans (171/243; 70.37%), whereas erythromycin and tetracycline resistance were particularly frequent among isolates from clinically healthy cats (26/32; 81.25% and 23/32; 71.88%, respectively). Clindamycin resistance was similar across the larger groups, ranging from 52.38% in clinically affected humans to 55.56% in clinically healthy dogs. Ciprofloxacin resistance was more frequent among clinically healthy feline isolates (6/32; 18.75%) than among clinically healthy humans (8/243; 3.29%) or canine isolates (1/18; 5.56%). No consistent pattern indicating higher antimicrobial resistance in clinically affected hosts was apparent across the antimicrobial panel. Because the clinically affected feline and canine groups comprised only two and four isolates, respectively, percentages obtained for these subgroups were interpreted descriptively.

A total of 89 distinct phenotypic antimicrobial susceptibility profiles were identified among the 320 *S. epidermidis* isolates. Of these, 39 resistance profiles were recurrent, occurring in at least two isolates and collectively accounting for 259/320 isolates (80.94%), whereas 49 resistance profiles were unique to individual isolates. An additional 12 isolates were susceptible to all antimicrobial agents tested. The most frequent recurrent resistance profile comprised resistance to oxacillin, clindamycin, erythromycin, and tetracycline (OX–DA–E–TET; *n* = 30), followed by OX–E–TET (*n* = 22), OX–E (*n* = 18), OX alone (*n* = 17), and OX–DA–E (*n* = 17).

Considerable phenotypic diversity was observed across the study population, with substantial overlap of recurrent resistance profiles among isolates originating from different host and clinical-status groups. Recurrent resistance profiles, together with the 12 fully susceptible isolates, are presented in Supplementary Table S9. Resistance profiles occurring in only a single isolate were retained in the complete susceptibility dataset but were not individually listed among the recurrent profiles. Detailed data on phenotypic antimicrobial resistance of all tested isolates, determined using the disk diffusion method, are presented in Table 1.

#### 2.3.2. Minimum Inhibitory Concentration and Minimum Bactericidal Concentration for Selected Antimicrobials

Among the 320 analyzed *S. epidermidis* isolates, no teicoplanin-resistant isolates were detected. Resistance to vancomycin was uncommon and detected in only 4 isolates (1.25%). In contrast, the highest overall resistance rate was observed for oxacillin (284/320; 88.75%), followed by erythromycin (245/320; 76.56%), clindamycin (217/320; 67.81%), and tetracycline (208/320; 65.00%).

The observed resistance patterns were further reflected in the analysis of the relationship between the minimum inhibitory concentration (MIC) and the minimum bactericidal concentration (MBC). Analysis of the log_2_(MBC/MIC) ratio revealed considerable variability both among antimicrobial agents and among individual isolates. For some isolate–antimicrobial combinations, only minor differences between MIC and MBC values were observed, whereas in others substantially higher concentrations were required to achieve a bactericidal effect. This variability was observed irrespective of the MIC susceptibility category, indicating marked isolate-dependent differences in bactericidal activity. A comprehensive heatmap illustrating the log_2_(MBC/MIC) ratios for all analyzed isolates, together with the corresponding MIC values and susceptibility classifications, is presented in Supplementary Figure S2.

### 2.4. Detection of Resistance Genes Using PCR-Based Genotypic Analysis

The analysis of antimicrobial resistance genes (Table 2) revealed that *blaZ* was the most frequently detected determinant, being present in 162/320 isolates (50.63%). The *tetK* gene was detected in 66/320 isolates (20.63%), followed by *mecA* in 53/320 (16.56%), *fusB* in 26/320 (8.13%), *ermC* in 24/320 (7.50%), and *aac(6′)-Ie-aph(2″)-Ia* in 13/320 isolates (4.06%). The *tetO* and *ermA* genes were detected in 11/320 (3.44%) and 9/320 isolates (2.81%), respectively, whereas *mupA* was identified in 5/320 isolates (1.56%). The *tetL* and *vanA* genes were rare, each being detected in only 3/320 isolates (0.94%). In contrast, *mecC, tetM, ermB,* and *vanB* were not detected in any of the analysed isolates.

The distribution of resistance genes varied among the analysed host groups. The *blaZ* gene was detected in 107/243 (44.03%) clinically healthy human isolates, 16/21 (76.19%) clinically affected human isolates, 23/32 (71.88%) clinically healthy feline isolates, 2/2 (100%) clinically affected feline isolates, 11/18 (61.11%) clinically healthy canine isolates, and 3/4 (75.00%) clinically affected canine isolates. The *mecA* gene was detected in 32/243 (13.17%) clinically healthy human isolates, 1/21 (4.76%) clinically affected human isolates, 14/32 (43.75%) clinically healthy feline isolates, 4/18 (22.22%) clinically healthy canine isolates, and 2/4 (50.00%) clinically affected canine isolates, whereas it was absent from the two clinically affected feline isolates.

Among tetracycline resistance determinants, *tetK* was the most prevalent, occurring in 66/320 isolates (20.63%), whereas *tetO* and *tetL* were considerably less frequent. The *ermC* gene was more common than *ermA*, while *ermB* was not detected. The *fusB* gene occurred in 26/320 isolates (8.13%), predominantly among human-derived isolates. The *mupA* and *vanA* genes occurred only sporadically.

Analysis of the complete resistance-gene patterns identified 42 distinct genotypic profiles among the 320 isolates. Ninety isolates (28.13%) did not harbour any of the 15 resistance genes investigated. Among gene-positive profiles, the most common profile consisted of *blaZ* alone, observed in 76/320 isolates (23.75%), followed by *blaZ + tetK* in 27/320 isolates (8.44%), *tetK* alone in 23/320 isolates (7.19%), and *blaZ + mecA* in 13/320 isolates (4.06%). Further recurrent profiles included *fusB* alone in 9/320 isolates (2.81%), *blaZ + fusB* in 8/320 (2.50%), *mecA* alone in 8/320 (2.50%), and *ermC* alone in 6/320 isolates (1.88%). The profiles *blaZ + mecA + aac(6′)-Ie-aph(2″)-Ia + tetK* and *blaZ + mecA + tetK* were each identified in 5/320 isolates (1.56%), while the remaining genotypic profiles occurred in four or fewer isolates.

### 2.5. Genotype–Phenotype Concordance Based on PCR

Genotype–phenotype concordance was assessed for the complete collection of 320 *S. epidermidis* isolates by comparing PCR-detected antimicrobial resistance genes with phenotypic susceptibility classifications obtained by disk diffusion and MIC testing. Disk diffusion and MIC results were analysed independently.

Overall agreement between PCR-detected resistance genes and phenotypic resistance was limited for most antimicrobial classes. For oxacillin, the combined detection of *mecA/mecC* showed essentially no agreement with disk-diffusion classification (Cohen’s κ = 0.004) and worse-than-chance agreement with MIC-based classification (κ = −0.025), with significant discordance predominantly directed towards phenotype-resistant/genotype-negative isolates (McNemar *p* < 0.001 for both methods). In contrast, *blaZ* showed limited agreement with penicillin and ampicillin disk-diffusion phenotypes (κ = 0.246 and 0.205, respectively). In both comparisons, discordance remained significantly skewed towards genotype-positive/phenotype-susceptible isolates (McNemar *p* = 0.002 for penicillin and *p* < 0.001 for ampicillin).

Similarly, weak agreement was observed for macrolide, lincosamide, and tetracycline resistance. For erythromycin and clindamycin, discordance was consistently dominated by phenotype-resistant/genotype-negative isolates, whereas for *blaZ*-associated penicillin and ampicillin resistance the opposite pattern was observed, with significant predominance of genotype-positive/phenotype-susceptible isolates (McNemar *p* < 0.001).

The strongest PCR-based genotype–phenotype agreement was observed between *aac(6′)-Ie-aph(2″)-Ia* and gentamicin resistance (κ = 0.406), corresponding to moderate agreement, with no significant directional asymmetry (McNemar *p* = 0.065). In contrast, *mupA* showed only slight agreement with mupirocin resistance (κ = 0.192), while *fusB* showed no agreement with fusidic acid resistance (κ = −0.027). Detailed genotype–phenotype concordance results, including concordant and discordant categories, Cohen’s κ values, McNemar test results, and the direction of discordance, are provided in Supplementary Table S10.

### 2.6. Analysis of Bacterial Growth Curves and Biofilm Formation

Growth kinetics were analysed for 15 representative *S. epidermidis* isolates (hHo236, hHo238, hHo240, hHo242, hHo243, sHo21, hFe5, sFe1, sFe2, hCa12, hCa13, hCa14, hCa16, sCa1, and sCa4), together with two reference strains (*S. epidermidis* PCM 1869 and *Staphylococcus aureus* ATCC 43300), following incubation at 37 °C, 38 °C, and 39 °C. Growth curves obtained at the three incubation temperatures for the analysed isolates are presented in Supplementary Figure S3a–S3u. The specific growth rate (*μ*, h^−1^) and corresponding doubling time (*tD*, h), calculated individually for each isolate and reference strain at each incubation temperature, are presented in Supplementary Table S11, whereas temperature-dependent differences in doubling time are visualized in Supplementary Figure S4.

For descriptive purposes, the observed responses were grouped into five temperature-dependent patterns. A gradual decrease in growth rate with increasing temperature was observed for hFe5 and the reference strain *S. aureus* ATCC 43300. A gradual increase was observed for hHo238, sHo21, and hCa16, whereas hHo240, hHo242, and sCa4 showed maximal growth rates at 38 °C. Five isolates (hHo236, hHo243, hCa13, hCa14, and sCa1) showed an initial decrease in growth rate at 38 °C followed by recovery at 39 °C. In contrast, sFe1, sFe2, hCa12, and the reference strain *S. epidermidis* PCM 1869 showed only limited temperature-dependent variation and were classified as having a temperature-independent growth pattern. The distribution of the analysed strains among the identified growth patterns and their relationship with biofilm-forming capacity are summarized in Table 3. Comparison of temperature-dependent growth kinetics with biofilm-forming capacity showed no consistent relationship between changes in planktonic growth and biofilm production. Isolates displaying similar growth responses to increasing temperature differed in their biofilm phenotypes, while isolates assigned to different growth patterns frequently showed comparable biofilm-forming capacities. Among the 15 representative *S. epidermidis* isolates included in the growth-kinetics analysis, 13/15 (86.7%) were classified as weak biofilm producers at 39 °C, 1/15 (6.7%) as a moderate producer, and 1/15 (6.7%) as a strong biofilm producer. None of the representative isolates was classified as a non-biofilm producer at this temperature. Thus, the temperature associated with maximal planktonic growth was strain dependent and did not consistently correspond to increased biofilm-forming capacity.

Biofilm-forming capacity was assessed after 24 h of incubation at 37 °C, 38 °C, and 39 °C. At 37 °C, 35/320 (10.94%) isolates were classified as non-biofilm producers, 27/320 (8.44%) as weak, 74/320 (23.13%) as moderate, and 184/320 (57.50%) as strong biofilm producers. At 38 °C, 18/320 (5.63%) isolates showed no biofilm formation, 85/320 (26.56%) were classified as weak, 50/320 (15.63%) as moderate, and 167/320 (52.19%) as strong producers. At 39 °C, 33/320 (10.31%) isolates were classified as non-biofilm producers, 74/320 (23.13%) as weak, 61/320 (19.06%) as moderate, and 152/320 (47.50%) as strong biofilm producers.

Quantitative analysis of biofilm production showed an overall decrease in OD_570_ values with increasing incubation temperature. Mean OD_570_ values were 1.398 ± 1.089 at 37 °C, 1.230 ± 1.020 at 38 °C, and 1.059 ± 0.902 at 39 °C.

Incubation temperature had a significant overall effect on quantitative biofilm production (likelihood-ratio χ^2^ = 102.24, df = 2, *p* < 0.001). Holm-adjusted pairwise comparisons showed significantly greater biofilm production at 37 °C than at 39 °C (estimated mean difference in OD_570_ = 0.339, adjusted *p* < 0.001). Biofilm production was also significantly greater at 38 °C than at 39 °C (estimated mean difference = 0.171, adjusted *p* < 0.001), and significantly greater at 37 °C than at 38 °C (estimated mean difference = 0.169, adjusted *p* < 0.001).

Host species also had a significant overall effect on quantitative biofilm production (likelihood-ratio χ^2^ = 29.79, df = 2, *p* < 0.001). Human-derived isolates exhibited significantly greater biofilm formation than feline-derived isolates (estimated mean difference in OD_570_ = 0.766, adjusted *p* < 0.001) and canine-derived isolates (estimated mean difference = 0.695, adjusted *p* = 0.0010), whereas no significant difference was observed between feline- and canine-derived isolates (estimated mean difference = 0.071, adjusted *p* = 0.773).

Clinical status of the host was also significantly associated with biofilm production (likelihood-ratio χ^2^ = 5.85, df = 1, *p* = 0.0155). Isolates obtained from clinically affected hosts showed higher quantitative biofilm production than isolates obtained from clinically healthy hosts (estimated mean difference in OD_570_ = 0.442, *p* = 0.015).

No significant interaction was detected between incubation temperature and host species (likelihood-ratio χ^2^ = 6.51, df = 4, *p* = 0.164) or between incubation temperature and clinical status (likelihood-ratio χ^2^ = 1.44, df = 2, *p* = 0.486). Accordingly, these interaction terms were not retained in the final additive model. The observed effects of host species and clinical status therefore did not show evidence of substantial modification across the three incubation temperatures.

Quantitative biofilm production and the distribution of biofilm-forming capacity categories according to incubation temperature and host origin are shown in Figure 4A and Figure 4B, respectively, while detailed quantitative data for individual isolates at each incubation temperature are provided in Supplementary Table S12.

### 2.7. Pathogenicity Assessment Using the Galleria mellonella Infection Model

Kaplan–Meier survival analysis demonstrated significant differences in *Galleria mellonella* (*G. mellonella*) larval survival among the tested inoculum concentrations and the PBS control for all eight analysed *S. epidermidis* isolates and the reference strain PCM 2532 (log-rank Mantel–Haenszel test, *p* ≤ 0.009). For seven strains, these differences were highly significant (*p* < 0.001), whereas significant differences were also observed for sCa2 (*p* = 0.001) and sHo21 (*p* = 0.009). Overall, larval survival decreased with increasing bacterial inoculum, demonstrating a clear dose-dependent effect on mortality.

At the highest inoculum concentration (OD_600_ = 0.5), larval mortality after 120 h ranged from 66.7% to 100%. The greatest larval mortality was observed for isolates hCa15 and hCa16, both of which caused 100% mortality (30/30 larvae) within the observation period. Isolates hCa14 and hHo240, as well as the reference strain PCM 2532, caused 90.0% mortality (27/30 larvae). Intermediate mortality was observed for hFe5 and sFe1 (76.7%; 23/30 larvae) and sHo21 (70.0%; 21/30 larvae), whereas sCa2 exhibited the lowest mortality at the highest inoculum concentration, with 66.7% mortality (20/30 larvae). Median survival time (LT_50_) at OD_600_ = 0.5 varied markedly among strains. The shortest LT_50_, 24 h, was recorded for hCa15, hCa16, hHo240, and PCM 2532, indicating rapid larval killing at the highest bacterial concentration. The LT_50_ was 48 h for hCa14, 72 h for hFe5 and sFe1, 96 h for sCa2, and 120 h for sHo21.

Survival in the PBS control group remained high throughout the experiment, with 90% of larvae surviving until the end of the 120 h observation period. A single death was recorded in the PBS control group. Despite substantial differences in virulence among individual isolates, no consistent qualitative pattern according to host species or clinical status was apparent. Isolates associated with relatively high and low larval mortality occurred across different host species and clinical-status categories, indicating considerable isolate-level variability. For example, the three healthy dog-derived isolates showed marked heterogeneity, with mortality at OD_600_ = 0.5 ranging from 90.0% for hCa14 to 100% for hCa15 and hCa16. Given the limited and uneven representation of isolates within individual host and clinical categories, no formal inferential comparisons between these groups were performed.

Detailed Kaplan–Meier survival curves are presented in Figure 5A–I, whereas log-rank Mantel–Haenszel test statistics (χ^2^ and *p*-values), LT_50_ values, and larval survival rates after 120 h at the highest inoculum density (OD_600_ = 0.5) are summarized in Table 4. At the highest inoculum density (OD_600_ = 0.5), Kaplan–Meier survival distributions differed significantly among the eight analysed *S. epidermidis* isolates and the reference strain (log-rank Mantel–Haenszel test, χ^2^ = 97.71, df = 8, *p* < 0.001), confirming substantial strain-dependent variation in virulence.

## 3. Discussion

The present study provides a comparative characterization of *S. epidermidis* isolates originating from humans, cats, and dogs, combining phenotypic, proteomic, molecular, and genomic approaches. Overall, the analysed population was highly heterogeneous, while clear segregation according to host species or clinical status was generally not observed. This finding is consistent with the substantial population diversity previously described for *S. epidermidis*, a species characterized by extensive recombination, frequent acquisition of mobile genetic elements, and coexistence of multiple clonal lineages [1,16]. The considerable overlap observed in MALDI-TOF MS PCA and hierarchical clustering, together with the absence of complete host-associated separation in the *rpoB*-based phylogeny, therefore suggests that variation within the species cannot be explained solely by host origin. MALDI-TOF MS clustering should, however, be interpreted as reflecting similarity of protein spectral profiles rather than genomic relatedness, and the observed predominance of particular host groups within some clusters should not be regarded as evidence of host-specific lineages.

The MLST results further demonstrated substantial genetic diversity, with 26 known sequence types identified among isolates for which complete profiles were obtained. Such diversity is consistent with previous population studies of *S. epidermidis*, in which numerous STs were identified within both clinical and carriage populations [16,17]. Importantly, several STs in the present study, including ST153 and ST87, occurred in isolates derived from more than one host species. Previous studies have likewise identified related or identical *S. epidermidis* lineages in humans and animals [17,18]. These observations support the concept that some *S. epidermidis* clonal lineages are not strictly restricted to a single host species. Nevertheless, detection of the same ST provides relatively low-resolution evidence of relatedness and cannot establish direct interspecies transmission. Confirmation of transmission would require substantially higher-resolution genomic comparison combined with appropriate epidemiological linkage.

Whole-genome phylogenetic analysis provided a higher-resolution view of the genetic relationships within the sequenced subset and further supported the marked genomic heterogeneity of the collection. The absence of a clear segregation of isolates according to host origin or clinical status is consistent with the broad population structure described for *S. epidermidis*, in which multiple phylogenetic lineages and sequence types coexist and extensive genomic diversity may occur even within individual hosts or epidemiological settings [19,20]. Recent population-genomic analyses involving more than 1800 *S. epidermidis* genomes identified four major phylogenetic groups and 237 sequence types, further emphasizing the highly diverse genomic architecture of this species [20]. Importantly, whole-genome phylogeny provides substantially greater discriminatory power than seven-locus MLST, and isolates sharing the same ST may nevertheless occupy different genomic backgrounds [20]. Accordingly, the occurrence of shared STs across human and animal isolates in the present study should be interpreted as evidence of broadly shared clonal backgrounds rather than proof of recent transmission.

Antimicrobial susceptibility testing revealed a high frequency of resistance to oxacillin, erythromycin, tetracycline, and clindamycin, whereas resistance to several other agents, including gentamicin, fluoroquinolones, chloramphenicol, and trimethoprim/sulfamethoxazole, was considerably less frequent. Methicillin and multidrug resistance are well recognized among *S. epidermidis*, particularly within healthcare-associated populations, but resistant strains have also been recovered from healthy humans and companion animals [2,13,18]. The extensive diversity of phenotypic resistance profiles observed here, together with their overlap between host groups, is therefore compatible with the heterogeneous and highly plastic resistome described for this species. Although some numerical differences were apparent among host groups, no consistent pattern indicating greater resistance among isolates from clinically affected hosts was evident. This observation should be interpreted cautiously because of the unequal group sizes, particularly the small numbers of clinically affected cats and dogs.

The molecular analyses further illustrated the complexity of antimicrobial resistance in this collection. PCR identified *blaZ* as the most frequent determinant, followed by *tetK*, *mecA*, *fusB*, and *ermC*, whereas WGS revealed a broader range of acquired resistance determinants, including *msrA*, *mphC*, *aac(6′)-Ie-aph(2″)-Ia*, *ANT(4′)-Ia*, *vgaA*, and genes associated with reduced susceptibility to selected topical antimicrobial or antiseptic compounds. The diversity of resistance determinants detected in *S. epidermidis* is consistent with its recognized capacity to acquire and maintain mobile genetic elements and its proposed role as a reservoir of antimicrobial resistance genes within staphylococcal populations [2,15,17]. Large-scale genomic studies similarly demonstrate substantial variation in the antimicrobial-resistance gene content of *S. epidermidis* and a strong association of particular high-risk lineages with expanded resistance repertoires [13,20]. Importantly, however, none of the apparent host-associated differences in WGS-detected acquired resistance genes remained significant after correction for multiple comparisons. Thus, the present data do not support a distinct host- or clinical-status-specific resistome within the sequenced subset.

Concordance between PCR- and WGS-based detection of antimicrobial resistance determinants was gene dependent. Almost perfect agreement was observed for *mecA* (92.7%; κ = 0.818), while *blaZ* showed moderate agreement (80.0%; κ = 0.541). Complete agreement was observed for *aac(6′)-Ie-aph(2″)-Ia*, whereas lower concordance was observed for *tetK* and several less prevalent determinants. Such variation indicates that resistance-gene detection may depend partly on the analytical method used and that targeted PCR and genome-based detection should not necessarily be regarded as interchangeable. Differences between these approaches may reflect incomplete recovery of particular loci in draft genome assemblies, sequence divergence affecting genome-based gene calling, differences in detection thresholds, or the highly targeted nature of PCR assays. Conversely, WGS may identify resistance determinants that are not included in predefined PCR panels and therefore provides a broader overview of the resistome [21,22]. The gene-specific differences observed in the present study therefore support the complementary use of targeted molecular assays and WGS when detailed characterization of antimicrobial resistance determinants is required.

One of the notable findings was the limited concordance between the presence of selected resistance determinants and phenotypic susceptibility classifications. This was particularly evident for oxacillin, for which *mecA* detection showed little or no agreement with phenotypic resistance, as well as for macrolides, lincosamides, and tetracyclines. Genotype–phenotype discrepancies have previously been reported in *S. epidermidis*, including heterogeneous expression of methicillin resistance and discordance between *mecA* carriage and oxacillin susceptibility [23,24,25]. The phenotype-resistant/genotype-negative pattern observed for several antimicrobial classes in the present study may additionally reflect resistance mechanisms not covered by the PCR panel, regulatory or mutational mechanisms, or limitations inherent to genotype-based prediction. Conversely, detection of a resistance gene in a phenotypically susceptible isolate does not necessarily imply constitutive expression of that determinant. Recent genomic studies have similarly shown that the relationship between resistance genes and phenotypic susceptibility in *S. epidermidis* can vary according to the determinant investigated [26]. Consequently, phenotypic and genotypic approaches should be regarded as complementary rather than interchangeable in the characterization of antimicrobial resistance.

Biofilm formation was common across the collection, consistent with the central role of this phenotype in *S. epidermidis* persistence and opportunistic pathogenicity [3,11]. Quantitative biofilm biomass showed an overall decrease with increasing incubation temperature. Biofilm production differed significantly among all three incubation temperatures, with the highest quantitative biofilm biomass observed at 37 °C and the lowest at 39 °C. Temperature is known to influence the structural and physiological properties of *S. epidermidis* biofilms [27], although direct comparisons with the present experiment are limited because previous studies have used different temperatures, exposure conditions, and biofilm models. The present results therefore indicate that relatively small changes within the physiologically relevant temperature range may modify biofilm production under the conditions used here, rather than demonstrating a universal inhibitory effect of increased temperature. Human-derived isolates produced significantly greater biofilm biomass than feline- and canine-derived isolates. Clinical status was also significantly associated with quantitative biofilm production, with isolates obtained from clinically affected hosts showing higher estimated biofilm biomass than those obtained from clinically healthy hosts. However, this association should be interpreted cautiously given the unequal representation of clinical-status categories across host groups. This difference may reflect characteristics of the sampled isolate population and should not be interpreted as evidence of an inherently greater biofilm-forming capacity of isolates associated with a particular host or clinical status.

Growth-kinetics experiments provided additional evidence of marked strain-level heterogeneity. Five different responses to increasing temperature were observed, and the temperature associated with the highest planktonic growth rate differed among isolates. Moreover, no consistent relationship was identified between temperature-dependent planktonic growth and biofilm-forming capacity. This distinction is biologically plausible because biofilm development represents a regulated multicellular phenotype rather than a direct consequence of rapid planktonic replication [1,11]. Accordingly, the results suggest that adaptation of *S. epidermidis* to changing temperature involves strain-specific responses and that planktonic fitness alone does not predict biofilm biomass.

The *G. mellonella* model demonstrated a clear inoculum-dependent effect on larval survival and substantial variation in killing capacity among the selected *S. epidermidis* isolates. Previous studies have successfully used *G. mellonella* to evaluate *S. epidermidis* infection and changes in virulence-associated phenotypes, supporting its utility as a comparative in vivo model [28,29]. In the present study, isolates associated with rapid larval mortality occurred among different host species and clinical categories, indicating pronounced strain-dependent variability. Given the deliberately selected and limited isolate panel, together with the uneven representation of host and clinical-status categories, these observations should not be extrapolated to infer greater virulence of isolates from a particular host species or clinical category. Rather, the findings indicate that isolates recovered from both healthy and clinically affected hosts may express markedly different pathogenic potential in this experimental model.

Several limitations should be considered when interpreting these findings. The isolate collection was unevenly distributed among host species and clinical categories, with human-derived and clinically healthy isolates representing the largest groups, which may have reduced statistical power for comparisons involving the smaller feline, canine, and clinically affected groups. In addition, WGS, growth-kinetics, and *Galleria mellonella* experiments were performed on selected subsets rather than the complete isolate collection. Although core-genome phylogenetic analysis provides substantially higher discriminatory power than MLST, the current WGS subset and available epidemiological metadata remain insufficient to infer direct transmission events between humans and companion animals. The biofilm experiments were performed at 37 °C, 38 °C, and 39 °C to represent the selected physiological temperature range relevant to the investigated host species; however, temperatures below 37 °C were not examined, which limits conclusions regarding biofilm formation under lower-temperature conditions. Furthermore, the observed PCR–WGS discrepancies cannot be attributed to a single technical or biological mechanism without additional targeted validation. Finally, biofilm formation and virulence were evaluated using in vitro and *G. mellonella* models, respectively, which reproduce only selected aspects of the complex interaction between *S. epidermidis* and its mammalian hosts.

Taken together, the present findings emphasize the extensive phenotypic and genetic diversity of *S. epidermidis* circulating among humans and companion animals. The occurrence of shared sequence types, overlapping resistance profiles, and the absence of complete host-associated segregation in the genomic analyses are compatible with a population in which at least some lineages and resistance determinants are not strictly host restricted, while the available data do not demonstrate direct interspecies transmission. The simultaneous presence of antimicrobial resistance, biofilm-forming capacity, and variable pathogenic potential among isolates from both healthy and clinically affected hosts further illustrates why *S. epidermidis* should be considered within a One Health surveillance framework. Companion animals may represent an additional component of the ecological network in which CoNS and their resistance determinants circulate, but higher-resolution genomic studies involving epidemiologically linked human–animal populations will be required to define the extent and direction of such exchange [18,30].

## 4. Materials and Methods

### 4.1. Staphylococcus epidermidis Strain Collection

A total of 320 *S. epidermidis* isolates were included in the study. The isolates originated from an archival bacterial collection maintained at the Department of Epizootiology and the Clinic of Birds and Exotic Animals, Faculty of Veterinary Medicine, Wroclaw University of Environmental and Life Sciences, Wroclaw, Poland. The samples were collected between 2013 and 2022 from humans, cats, and dogs in Poland.

The 320 isolates originated from 243 individual hosts, including 191 humans, 33 cats, and 19 dogs. More than one isolate could originate from the same individual; however, each isolate represented a distinct anatomical sampling site. Among the human-derived isolates, 243 were obtained from 175 clinically healthy individuals, whereas 21 originated from 16 clinically affected individuals. Among the feline isolates, 32 were obtained from 31 clinically healthy cats and two from two clinically affected cats. The canine collection comprised 18 isolates obtained from 15 clinically healthy dogs and four isolates obtained from four clinically affected dogs. The distribution of isolates according to host species, clinical status, sampling site, and number of individual hosts is summarized in Table 5.

For the purposes of the present study, individuals were classified as clinically healthy when no clinical signs of disease were observed at the time of sampling. Individuals classified as clinically affected presented clinical abnormalities at the time of sample collection, including, among others, coughing, conjunctival discharge, wounds, or other skin lesions, as described for the original sampling framework [31,32]. The clinical-status classification referred exclusively to the condition of the host at the time of sampling and did not imply that *S. epidermidis* was established as the causative agent of the observed clinical signs.

Because the numbers of isolates and individual hosts differed substantially among host species and clinical-status categories, particularly in the clinically affected feline and canine groups, comparisons involving these smaller subgroups were interpreted cautiously and were considered primarily descriptive where appropriate.

The original sampling scheme included epidemiologically linked humans and companion animals originating from the same households. Within this paired subset, four households yielded *S. epidermidis* isolates from both a human participant and a companion animal, including one human–cat household and three human–dog households. These isolates were considered epidemiologically linked solely based on household information recorded at the time of sampling. Their occurrence within the same household was not interpreted as evidence of direct interspecies transmission.

The human-derived isolates originated from a previously approved research project (approval No. KB-814/2019) granted by the Bioethics Committee of the Medical University of Wroclaw, Poland. Biological material was collected using non-invasive swab sampling, and written informed consent was obtained from all participants prior to sample collection. Animal-derived isolates were obtained as part of the same sampling project using non-invasive swab collection. According to the Local Ethical Committee for Animal Experiments in Wroclaw, this sampling did not constitute an experimental procedure under applicable Polish regulations and therefore did not require separate ethical approval. The present study utilized exclusively archived bacterial isolates and involved no additional procedures in either humans or animals. The original sampling framework and ethical considerations were described previously [32].

Before long-term storage, the isolates were cultured under standard conditions and subsequently preserved at −80 °C in Brain Heart Infusion (BHI) broth (Oxoid Ltd., Basingstoke, UK) supplemented with 40% (*v*/*v*) glycerol (Thermo Fisher Scientific, Kandel, Germany).

### 4.2. Identification of Staphylococcus epidermidis Isolates

All 320 isolates included in the study were recovered from frozen stocks kept at −80 °C and subcultured on Mannitol Salt Agar (Oxoid Ltd., Basingstoke, UK) and Columbia Agar supplemented with 5% sheep blood (Oxoid Ltd., Basingstoke, UK). Plates were incubated aerobically at 37 °C for 24 h. Preliminary identification of staphylococci was based on colony morphology and the tube coagulase test (Immunolab, Gdańsk, Poland).

Species-level identification was performed using matrix-assisted laser desorption/ionization time-of-flight mass spectrometry (MALDI-TOF MS) with a MALDI Biotyper Sirius mass spectrometer IVD IV (Bruker Corporation, Billerica, MA, USA). Protein extraction was performed using the standard ethanol–formic acid extraction procedure recommended by the manufacturer. Briefly, individual colonies were suspended in sterile water and mixed with absolute ethanol (Sigma-Aldrich, St. Louis, MO, USA). Following centrifugation, the resulting pellet was treated sequentially with 70% formic acid (Sigma-Aldrich, St. Louis, MO, USA) and acetonitrile (Honeywell, Charlotte, NC, USA), followed by a second centrifugation step. Subsequently, 1 μL of the supernatant was applied to a polished steel MALDI target plate, air-dried, and overlaid with 1 μL of saturated α-cyano-4-hydroxycinnamic acid matrix solution (10 mg/mL in acetonitrile–water–trifluoroacetic acid, 50:47.5:2.5, *v*/*v*/*v*).

Each isolate was analysed in duplicate, with two technical spectra obtained from each protein extract. Spectra were acquired and interpreted according to the manufacturer’s recommendations. Species-level identification was accepted for MALDI-TOF MS scores >2.000, and all 320 isolates included in the final study collection yielded identification scores above this threshold.

Proteomic similarity among the isolates was evaluated based on MALDI-TOF MS spectral peak profiles. Hierarchical clustering and principal component analysis (PCA) were performed using MALDI Biotyper Compass Explorer software, version 4.1.100 (Bruker Corporation), to visualize similarities and differences among the analysed isolates. These analyses were used to assess overall similarity of MALDI-TOF MS protein spectral profiles and were not interpreted as a measure of genomic relatedness.

Species identity was further confirmed by conventional PCR targeting the *S. epidermidis*-specific *gseA* gene, encoding a glutamic-acid-specific protease, according to the method described by Ikeda et al. [33]. Amplification was performed using primers *gseA* F157–177 (5′-ATGAAAAAGAGATTTTTATCT-3′) and *gseA* R660–642 (5′-GTTTGGTGACACTCTTAAG-3′), yielding a 503 bp amplification product. Cycling conditions consisted of an initial denaturation at 94 °C for 2 min, followed by 30 cycles of 94 °C for 10 s, 50 °C for 10 s, and 72 °C for 60 s, with a final extension at 72 °C for 4 min. PCR products were visualized by agarose gel electrophoresis.

To further support species identification and assess gene-based relatedness, partial sequencing of the *rpoB* gene was performed for the same subset of 67 *S. epidermidis* isolates subsequently subjected to whole-genome sequencing, as described in Section 4.3. PCR amplification of the *rpoB* gene was performed using previously published primers and amplification conditions described by Drancourt and Raoult [34]. The obtained amplicons were purified and sequenced bidirectionally. Consensus sequences were assembled and compared with reference sequences deposited in the NCBI GenBank database using BLASTn (https://blast.ncbi.nlm.nih.gov/Blast.cgi). Species identity was determined based on sequence similarity using a ≥99% identity threshold.

Phylogenetic relationships based on aligned partial *rpoB* sequences were reconstructed in MEGA11, version 11.0.11, using the Neighbor-Joining method, with evolutionary distances calculated according to the Tamura–Nei model. The robustness of the inferred tree topology was evaluated by bootstrap analysis with 1000 replicates. Reference sequences of selected *Staphylococcus* species retrieved from the NCBI GenBank database were included for comparison. The corresponding species names, strain identifiers, and GenBank accession numbers are provided in Supplementary Table S2.

### 4.3. Whole-Genome Sequencing and Comparative Genomic Analysis

Whole-genome sequencing was performed using the Oxford Nanopore Technologies platform with the SQK-RBK114-24 Rapid Barcoding Kit. Raw sequencing signals were basecalled and demultiplexed using Dorado v1.0.2 with the SUP model (dna_r10.4.1_e8.2_400bps_sup@v5.2.0). Sequence reads were subsequently quality filtered using Chopper, retaining reads with a minimum length of 500 bp and a mean Q-score ≥10 [35]. De novo genome assembly was performed using Flye v2.9 in nano-hq mode [36]. Assembly completeness and contamination were estimated using CheckM2 v1.0.2 [37]. Genome assemblies were evaluated according to total genome size, estimated completeness, contamination, and number of contigs. Assemblies with a total genome size of 2.2–2.8 Mb, completeness ≥ 95%, contamination ≤ 5%, and fewer than 200 contigs were considered suitable for downstream WGS-based antimicrobial resistance and concordance analyses. Fifty-five of the 67 assemblies met these predefined numerical acceptance criteria.

For pangenome reconstruction and construction of the core-genome phylogenetic backbone, a high-quality subset of 54 genome assemblies was used. These assemblies had completeness ≥ 99% and contamination ≤ 3%. Of the 55 assemblies retained for WGS-based antimicrobial resistance and concordance analyses, 54 met these more stringent criteria. The remaining assembly, sHo18, was retained for antimicrobial resistance and concordance analyses but was not included in the original Panaroo pangenome reconstruction or maximum-likelihood backbone-tree inference. Lower-quality assemblies with completeness ≥ 50% and contamination < 5% were evaluated separately for subsequent phylogenetic placement. In total, 12 assemblies were incorporated by phylogenetic placement, including sHo18 and 11 additional assemblies that did not meet the criteria for WGS-based antimicrobial resistance analyses. One assembly, hFe23, with completeness below 50%, was excluded from the phylogenetic analysis. Genome annotation was performed using Bakta v1.12.1 [38].

#### 4.3.1. Multilocus Sequence Typing and Sequence Type Assignment

Multilocus sequence typing was performed in silico for all 67 *S. epidermidis* genome assemblies using the seven-locus MLST scheme comprising *arcC, aroE, gtr, mutS, pyrR, tpiA*, and *yqiL* [39]. Allelic profiles were compared with the *S. epidermidis* profiles available in the PubMLST database [40].

Complete and unambiguous seven-locus allelic profiles matching an existing database profile were assigned to the corresponding sequence type (ST). Profiles containing ambiguous allele calls were retained as unresolved and were not assigned to an ST, whereas profiles lacking one or more loci were classified as incomplete. Complete seven-locus profiles without an initial ST assignment were manually rechecked against the current PubMLST database before final classification.

#### 4.3.2. Pangenome Reconstruction and Core-Genome Phylogenetic Analysis

Pangenome reconstruction was performed using the 54 high-quality genome assemblies retained for comparative genomic analysis. Annotated assemblies were analysed using Panaroo v1.6.1 in strict clean mode [41]. Gene clusters present in at least 95% of the analysed genomes were defined as belonging to the core genome.

Individual core-gene clusters were aligned using MAFFT v7.5 [42], and the resulting alignments were concatenated to generate the core-genome alignment used for phylogenetic reconstruction. Maximum-likelihood phylogenetic analysis was performed using IQ-TREE v2.4.0 [43]. The optimal nucleotide substitution model was selected automatically using ModelFinder Plus, resulting in the GTR+F+R4 model [44]. Branch support was evaluated using 1000 SH-aLRT replicates [45].

To incorporate lower-quality assemblies into the phylogenetic framework without including them in the original pangenome reconstruction or backbone-tree inference, core-gene homologues were identified by BLASTn searches against the representative high-quality core-gene set. Identified sequences were added to the corresponding Panaroo per-gene alignments using MAFFT. Lower-quality assemblies were subsequently placed onto the maximum-likelihood backbone tree using EPA-ng v0.3.8 under the GTR+FC+G4 model [46] and processed using gappa v0.9.0 [47].

The resulting phylogenetic framework comprised the 54 high-quality assemblies used for backbone reconstruction together with lower-quality assemblies meeting the predefined placement criteria. Isolate identifiers correspond to those reported in Supplementary Table S5.

#### 4.3.3. Antimicrobial Resistance Determinant Analysis

Antimicrobial resistance determinants were screened against the Comprehensive Antibiotic Resistance Database (CARD) [48] using ABRicate v1.4.0 [49]. For the analyses reported in the present study, antimicrobial resistance determinants were evaluated in the 55 genome assemblies retained according to the predefined WGS quality criteria. Hits were retained using minimum thresholds of 70% nucleotide identity and 60% sequence coverage. The CARD version used for the analysis was dated 3 April 2026.

Acquired antimicrobial resistance determinants were analysed separately from broadly distributed intrinsic resistance-associated genes, multidrug efflux systems, and regulatory components. Genes associated with reduced susceptibility to antiseptic compounds were also recorded.

### 4.4. Antimicrobial Susceptibility Testing

Phenotypic antimicrobial susceptibility was assessed by the disk diffusion method on Mueller–Hinton agar using standardized antimicrobial susceptibility testing procedures. The antimicrobial panel comprised amoxicillin/clavulanic acid (AMC, 20/10 μg), ampicillin (AMP, 10 μg), ciprofloxacin (CIP, 5 μg), chloramphenicol (C, 30 μg), clindamycin (DA, 2 μg), erythromycin (E, 15 μg), fusidic acid (FD, 10 μg), gentamicin (CN, 10 μg), linezolid (LZD, 30 μg), oxacillin (OX, 1 μg), mupirocin (MUP, 200 μg), penicillin G (P, 10 U), rifampin (RD, 5 μg), tetracycline (TET, 30 μg), tigecycline (TGC, 15 μg), tobramycin (TOB, 10 μg), and trimethoprim/sulfamethoxazole (SXT, 1.25/23.75 μg) (Liofilchem, Roseto degli Abruzzi, Italy), as well as marbofloxacin (MAR, 5 μg) (MASTDISCS^®^, Mast Group Ltd., Liverpool, UK). *S. aureus* ATCC 25923 was used as the quality control strain.

Inhibition zone diameters were interpreted using antimicrobial-specific criteria selected from the most appropriate available standards. Current Clinical and Laboratory Standards Institute (CLSI) M100 criteria were applied where active interpretive breakpoints for staphylococci were available [50]. For oxacillin, the species-specific CLSI disk-diffusion criterion for *S. epidermidis* using a 1-μg disk was applied. Marbofloxacin was interpreted according to veterinary-specific criteria provided in CLSI VET01S, 7th edition [51]. Where current CLSI criteria were unavailable, previously established interpretive criteria were retained, including those for amoxicillin/clavulanic acid, ampicillin, and tobramycin [52,53,54]. Fusidic acid and mupirocin were interpreted according to EUCAST Breakpoint Tables version 14.0 [55]; for mupirocin, the *S. aureus* breakpoint was used as a surrogate. Tigecycline was interpreted using the published disk-diffusion criterion for *Staphylococcus* spp. [56]. The disk contents, interpretive thresholds, applicable bacterial groups, and corresponding reference standards used for each antimicrobial agent are provided in Supplementary Table S13.

Minimum inhibitory concentrations of oxacillin, erythromycin, clindamycin, tetracycline, vancomycin, and teicoplanin were determined by broth microdilution in Mueller–Hinton broth according to CLSI methodology. Two-fold serial dilutions were prepared over concentration ranges of 0.03125–16 μg/mL for oxacillin, clindamycin, and tetracycline; 0.0625–32 μg/mL for erythromycin; and 0.5–128 μg/mL for vancomycin and teicoplanin. *Staphylococcus aureus* ATCC 29213 was used as the quality control strain. The MIC was defined as the lowest antimicrobial concentration that completely inhibited visible bacterial growth after incubation.

Minimum bactericidal concentrations were subsequently determined from wells showing no visible bacterial growth. Material from these wells was subcultured onto Brain Heart Infusion agar (Oxoid, Basingstoke, UK) and incubated under the conditions specified for the assay. For the purposes of the present study, the MBC was defined operationally as the lowest antimicrobial concentration from which no bacterial colonies were recovered. The relationship between inhibitory and bactericidal activity was expressed as the log_2_-transformed MBC/MIC ratio. A value of 0 indicated identical MIC and MBC values, whereas progressively higher values reflected increasing differences between the concentrations required to inhibit visible growth and to reach the defined bactericidal endpoint.

### 4.5. Detection of Antimicrobial Resistance Genes by PCR

Genomic DNA was extracted from all *S. epidermidis* isolates using the Genomic Mini AX Staphylococcus Spin Kit (A&A Biotechnology, Gdańsk, Poland) according to the manufacturer’s instructions. Genes associated with resistance to selected antimicrobial classes were detected using a combination of probe-based real-time PCR (qPCR) and conventional PCR assays. Probe-based qPCR assays were performed using target-specific hydrolysis probes labelled with FAM or HEX reporter dyes and a BHQ-1 quencher.

Probe-based qPCR assays were applied for the detection of *blaZ* [57], *mecA* [58], *ermA, ermB,* and *ermC* [59], *tetK* [32], and *tetL*, *tetM*, and *tetO* [60]. Conventional PCR assays were used for the detection of *mecC* [61], *aac(6′)-Ie-aph(2″)-Ia* [62], *fusB* [63], *vanA* [64], *vanB* [65], and *mupA* [66]. Detailed primer sequences, reaction conditions, and expected amplicon sizes for the conventional PCR assays were described previously [32] and in the respective original methodological references cited above. Positive controls carrying the respective resistance determinants were included in all PCR assays to verify reaction performance.

### 4.6. Bacterial Growth Kinetics and Biofilm Formation

Bacterial growth kinetics were assessed using a purposively selected panel of *S. epidermidis* isolates (hHo236, hHo238, hHo240, hHo242, hHo243, sHo21, hFe5, sFe1, sFe2, hCa12, hCa13, hCa14, hCa16, sCa1, and sCa4). The subset was selected to maximize representation across host origin, clinical-status categories, and contrasting antimicrobial resistance phenotypes and was not intended to constitute a statistically representative sample of the full isolate collection. Two reference strains, *S. epidermidis* PCM 1869 and *S. aureus* ATCC 43300, were included for comparison. Overnight bacterial cultures were diluted in Brain Heart Infusion (BHI) broth (Oxoid, Basingstoke, UK) to an initial OD_600_ of approximately 0.1. Cultures were incubated in triplicate at 37 °C, 38 °C, and 39 °C for 24 h under continuous agitation, and OD_600_ was measured at hourly intervals using a Tecan Infinite 200 Pro M Nano microplate reader (Tecan, Männedorf, Switzerland).

Because the onset and duration of the exponential growth phase varied among isolates and incubation temperatures, the exponential phase was identified separately for each individual growth curve. For each isolate and incubation temperature (37 °C, 38 °C, and 39 °C), the specific growth rate (*μ*, h^−1^) was calculated as the slope of the linear regression fitted to the natural logarithm of OD_600_ values during the exponential growth phase. The corresponding doubling time (*tD*, h) was calculated according to the equation *tD* = ln(2)/*μ*. For descriptive comparison, temperature-dependent growth responses were categorized according to changes in the specific growth rate across the three incubation temperatures as follows: gradual decrease, gradual increase, maximum growth rate at 38 °C, decrease followed by recovery, or temperature-independent growth. These categories were used solely to summarize the observed growth-response patterns and were not treated as independent inferential endpoints.

Biofilm-forming capacity was evaluated for all *S. epidermidis* isolates using the crystal violet microtiter plate assay as previously described [67]. *Staphylococcus aureus* ATCC 29213 was used as the positive control, whereas sterile Tryptone Soya Broth (TSB; Oxoid, Basingstoke, UK) served as the negative control. For each isolate and incubation temperature, the assay was performed in two independent experiments, with six technical replicates per experiment. Following crystal violet staining and solubilization, biofilm biomass was quantified by measuring absorbance at 570 nm (A_570_), and the mean A_570_ value calculated from all replicate measurements was used for subsequent analyses.

The optical density cut-off (ODc) was calculated separately for each incubation temperature as the mean A_570_ of the corresponding negative controls plus three standard deviations (SD), according to the method described by Stepanović et al. [67]. Because the ODc values obtained at 37 °C, 38 °C, and 39 °C were highly similar, a common cut-off value of 0.2122 was applied to all temperatures to ensure consistent classification across experimental conditions. Isolates were classified as non-biofilm producers (A_570_ ≤ ODc), weak biofilm producers (ODc < A_570_ ≤ 2 × ODc), moderate biofilm producers (2 × ODc < A_570_ ≤ 4 × ODc), or strong biofilm producers (A_570_ > 4 × ODc). Accordingly, the numerical thresholds used in the present study were ≤0.2122, >0.2122–0.4244, >0.4244–0.8488, and >0.8488, respectively.

### 4.7. Galleria mellonella Infection Model 

The pathogenicity of selected *Staphylococcus epidermidis* isolates was investigated using the *Galleria mellonella* larval infection model. The experimental procedure was based on previously published protocols [68,69] and incorporated modifications described in our earlier study [32]. A panel of eight representative *S. epidermidis* isolates (hHo240, sHo21, hFe5, sFe1, hCa14, hCa15, hCa16, and sCa2) was selected to encompass different host species, clinical-status categories, and antimicrobial resistance profiles. The reference strain *S. epidermidis* PCM 2532 was included as a comparator.

Overnight cultures were collected by centrifugation at 3500 rpm and washed three times with sterile phosphate-buffered saline (PBS; Argenta, Poznań, Poland). Bacterial suspensions were subsequently adjusted to OD_600_ values of 0.5, 0.1, and 0.01. To estimate the viable bacterial concentrations corresponding to these optical-density-based inocula, quantitative culture was performed separately using *S. epidermidis* isolate sCa1 as a calibration strain. Viable counts of approximately 1.6 × 10^8^ CFU/mL, 3.2 × 10^7^ CFU/mL, and 4.0 × 10^6^ CFU/mL were obtained at OD_600_ values of 0.5, 0.1, and 0.01, respectively. Based on the 10 μL injection volume, these concentrations corresponded to approximate doses of 1.6 × 10^6^, 3.2 × 10^5^, and 4.0 × 10^4^ CFU per larva, respectively. These CFU values were used as approximate estimates of the inoculum size and were not determined individually for each isolate included in the infection experiment.

For each isolate and inoculum concentration, 30 larvae with a body weight of 300 ± 30 mg were used and divided into three replicate groups of 10 individuals. Each larva received 10 μL of the corresponding bacterial suspension injected directly into the hemocoel using a 100 μL Hamilton microsyringe (Hamilton Bonaduz AG, Bonaduz, Switzerland). A single common negative-control group consisting of 10 larvae injected with sterile PBS was included in the experiment.

Following inoculation, larvae were maintained at 37 °C for 120 h and examined at 24 h intervals. Larvae were classified as dead when complete melanization was present and no response to tactile stimulation could be elicited. For survival analysis, the time of death of each larva was assigned to the observation time point at which mortality was first recorded. Larvae remaining alive at 120 h were treated as right-censored observations.

Kaplan–Meier survival curves were generated using individual larval time-to-event data. Survival distributions for each bacterial isolate and inoculum concentration were compared with the common PBS control using the log-rank (Mantel–Haenszel) test. Statistical analyses were performed in JASP version 0.19.7.1 (JASP Team, University of Amsterdam, Amsterdam, The Netherlands).

### 4.8. Statistical Analysis

Statistical analyses were conducted using Statistica 13 (TIBCO Software Inc., Tulsa, OK, USA) and JASP version 0.19.7.1 (JASP Team, University of Amsterdam, Amsterdam, The Netherlands). Quantitative biofilm formation, expressed as absorbance at 570 nm (A_570_), was analysed using a linear mixed-effects model accounting for the hierarchical and repeated-measures structure of the dataset. Incubation temperature, host species, and clinical status were included as fixed effects. Individual host identity was included as a random effect, with isolate identity nested within host identity, thereby accounting both for repeated measurements of individual isolates across the three incubation temperatures and for the occurrence of multiple isolates originating from the same host. Interactions between incubation temperature and host species and between incubation temperature and clinical status were initially evaluated. As neither interaction was statistically significant, the final model included the corresponding additive effects. Overall fixed effects were assessed using likelihood-ratio tests. Pairwise comparisons were performed using estimated marginal means with Holm adjustment for multiple comparisons. Statistical significance was defined as *p* < 0.05.

Associations between the presence of individual acquired antimicrobial resistance determinants and host species or clinical status within the WGS subset were assessed using Fisher’s exact test. To account for multiple comparisons, *p*-values were adjusted using the Benjamini–Hochberg false discovery rate procedure. Genotype–phenotype concordance was assessed by comparing phenotypic antimicrobial susceptibility classifications with detection of the corresponding resistance determinants using Cohen’s kappa coefficient and McNemar’s test. The same statistical approach was applied to assess concordance between PCR- and WGS-based detection of antimicrobial resistance genes. For binary genotype–phenotype concordance analyses, MIC results classified as intermediate (I) were grouped with susceptible/non-resistant results. Disk-diffusion results recorded as R/S were treated as resistant for the descriptive and concordance analyses.

Survival outcomes from the *G. mellonella* infection experiments were analysed using the Kaplan–Meier method, and differences between survival distributions were assessed using the log-rank (Mantel–Haenszel) test. Survival distributions were compared among inoculum concentrations and the PBS control for each strain, as well as among the analysed strains at the highest inoculum density. Formal inferential comparisons according to host species or clinical status were not performed because of the limited and uneven representation of isolates within these categories.

Analyses related to whole-genome sequencing, pangenome reconstruction, and phylogenetic inference were carried out using the bioinformatic software and procedures described in Section 4.3.

## Figures and Tables

**Figure 1:**
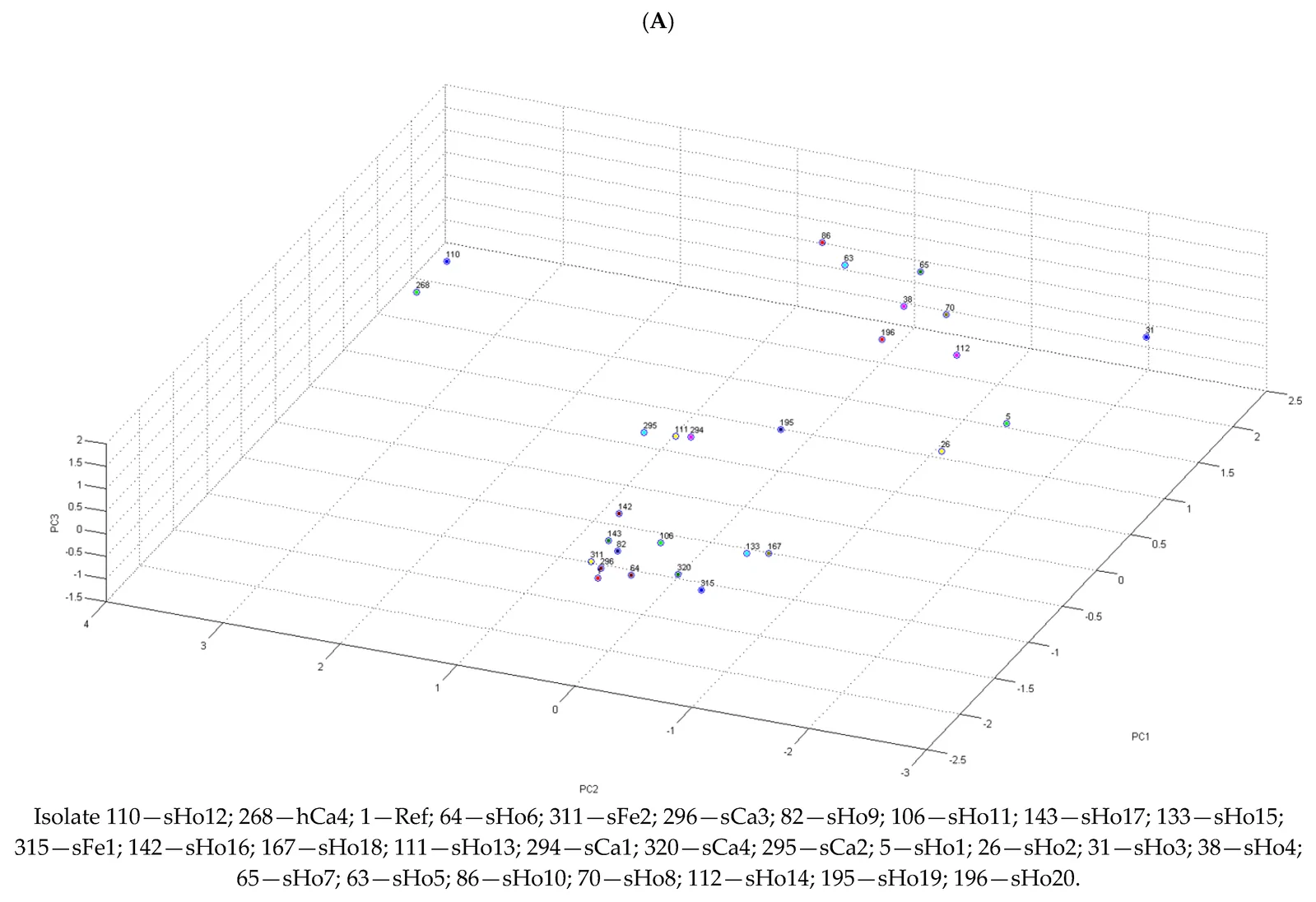

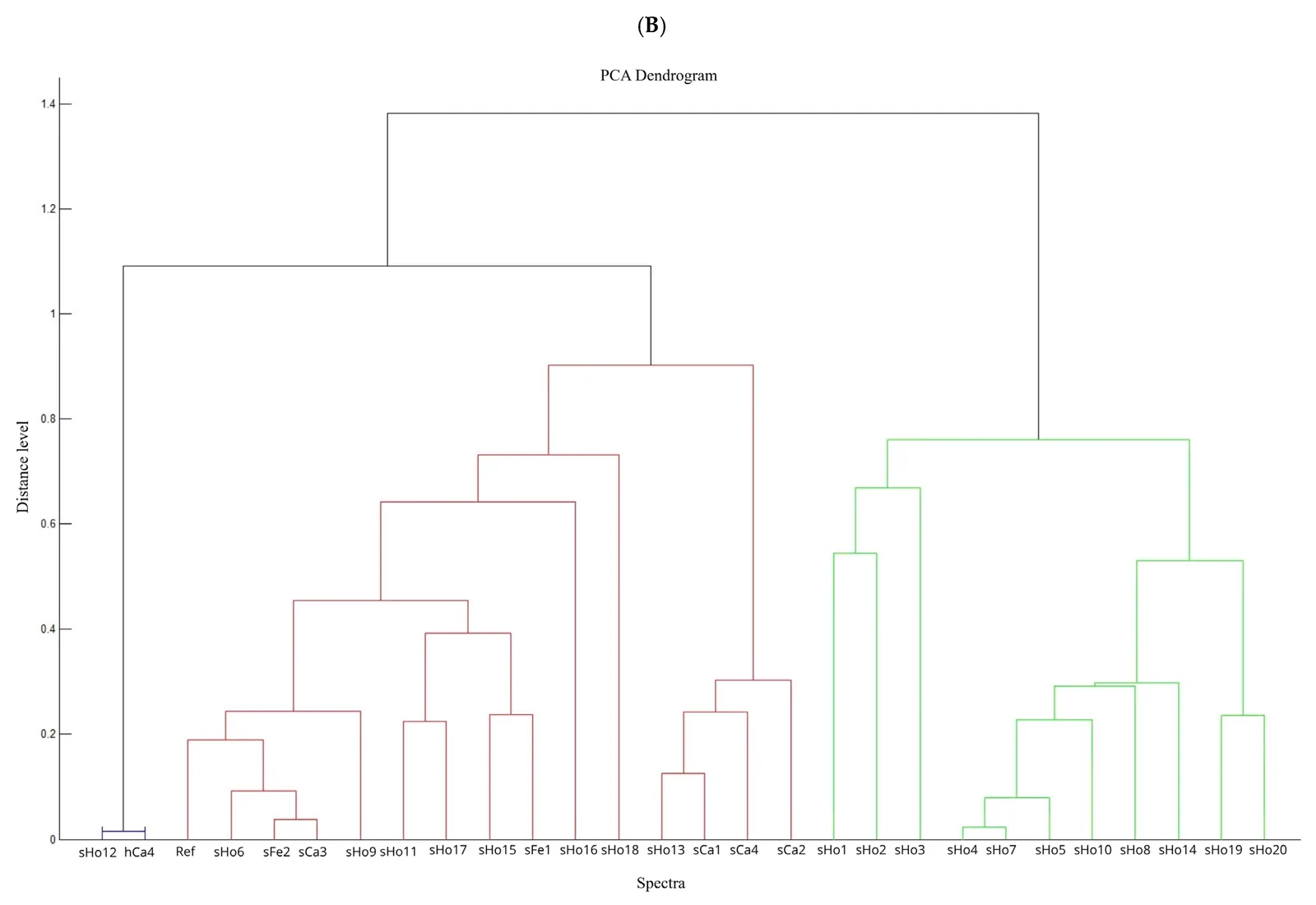
MALDI-TOF MS analysis of *Staphylococcus epidermidis* isolates obtained from clinically affected hosts. (**A**) Principal component analysis (PCA) visualization of isolates obtained from clinically affected humans, cats, and dogs together with an *S. epidermidis* reference strain. (**B**) Hierarchical clustering dendrogram of the same isolates based on MALDI-TOF MS spectral profiles. Numbers shown next to individual isolates correspond to PCA numbers; the corresponding study isolate IDs are provided in the figure legend. Branch colors indicate clusters identified by hierarchical clustering based on MALDI-TOF MS spectral similarity and do not represent host species or clinical status.

**Figure 2:**
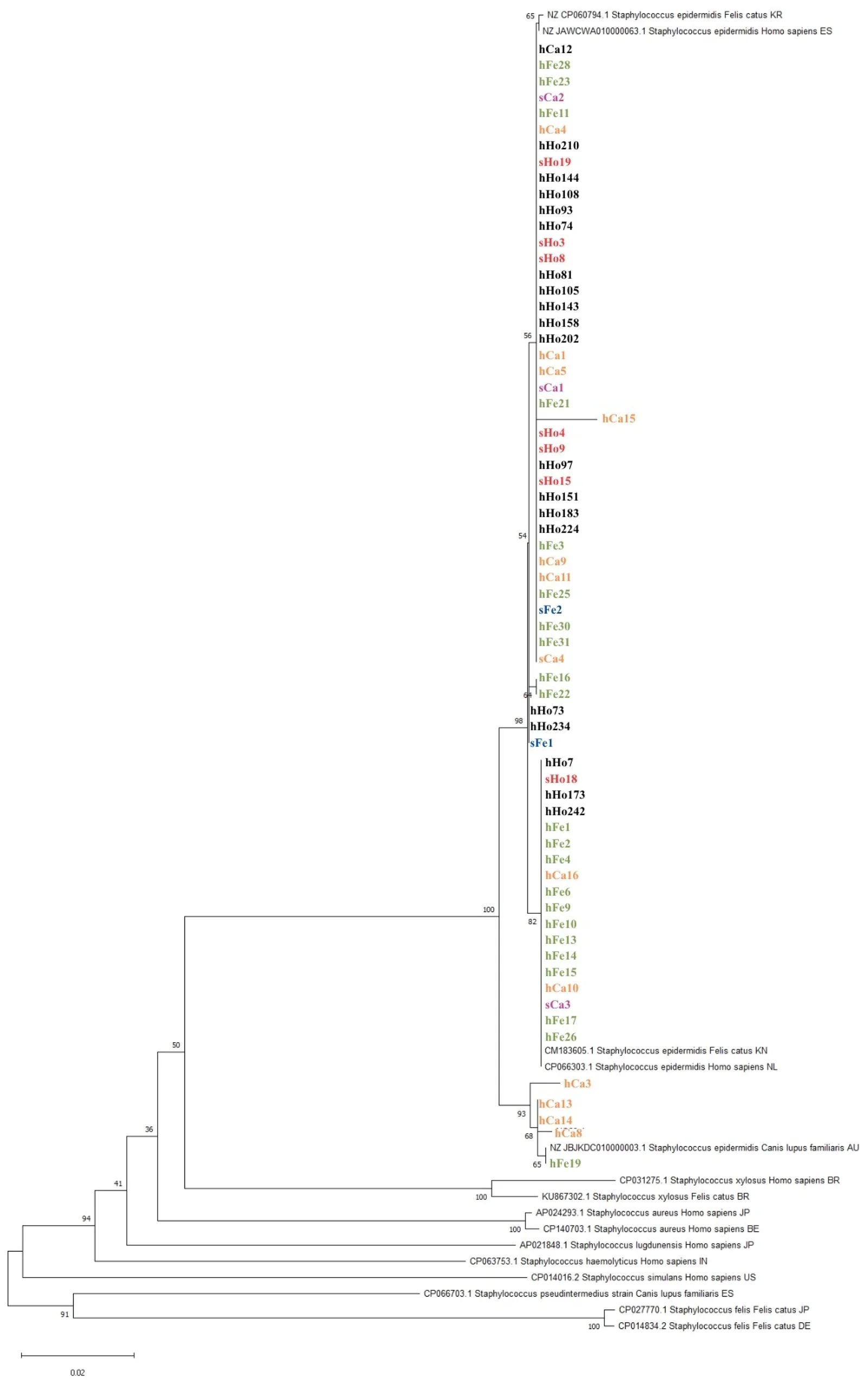
Phylogenetic relationships among *Staphylococcus epidermidis* isolates based on partial *rpoB* gene sequences. The tree was reconstructed in MEGA11 using the Neighbor-Joining method, with evolutionary distances calculated according to the Tamura–Nei model. The robustness of the inferred topology was assessed using 1000 bootstrap replicates. Reference sequences of selected *Staphylococcus* species retrieved from the NCBI GenBank database were included for comparison. Among the study isolates, label colours indicate host species and clinical status: black, clinically healthy humans; red, clinically affected humans; green, clinically healthy cats; blue, clinically affected cats; orange, clinically healthy dogs; and pink, clinically affected dogs. Reference sequences are identifiable by their GenBank accession numbers and species names. Bootstrap values are shown at the corresponding nodes, and the scale bar indicates nucleotide substitutions per site. Isolate identifiers correspond to those provided in Supplementary Table S2.

**Figure 3:**
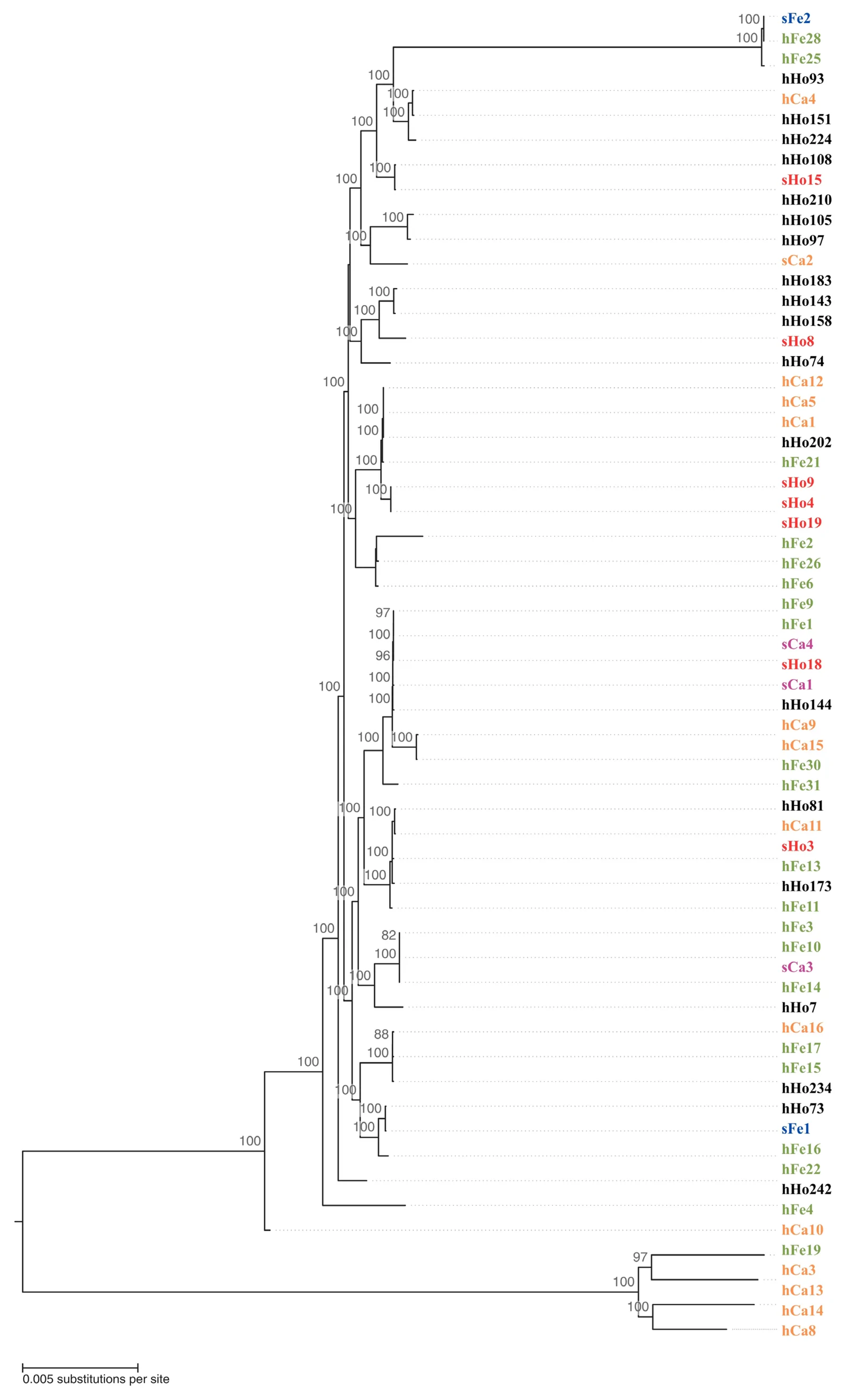
Core-genome phylogenetic relationships among *Staphylococcus epidermidis* isolates. The maximum-likelihood backbone phylogeny was reconstructed from a concatenated core-gene alignment generated from 54 high-quality *S. epidermidis* genome assemblies. Core genes were defined as genes present in ≥95% of the analysed high-quality genomes. Individual core-gene clusters were aligned using MAFFT, and the concatenated alignment was analysed using IQ-TREE. Lower-quality genome assemblies fulfilling the predefined placement criteria were subsequently incorporated into the phylogenetic framework using EPA-ng and gappa. The final phylogenetic tree comprises 66 isolates. Label colours indicate host species and clinical status: black, clinically healthy humans; red, clinically affected humans; green, clinically healthy cats; blue, clinically affected cats; orange, clinically healthy dogs; and pink, clinically affected dogs. Branch support values are indicated at the corresponding nodes. The scale bar represents 0.005 substitutions per site. The phylogenetic inclusion status of individual isolates is provided in Supplementary Table S5.

**Figure 4:**
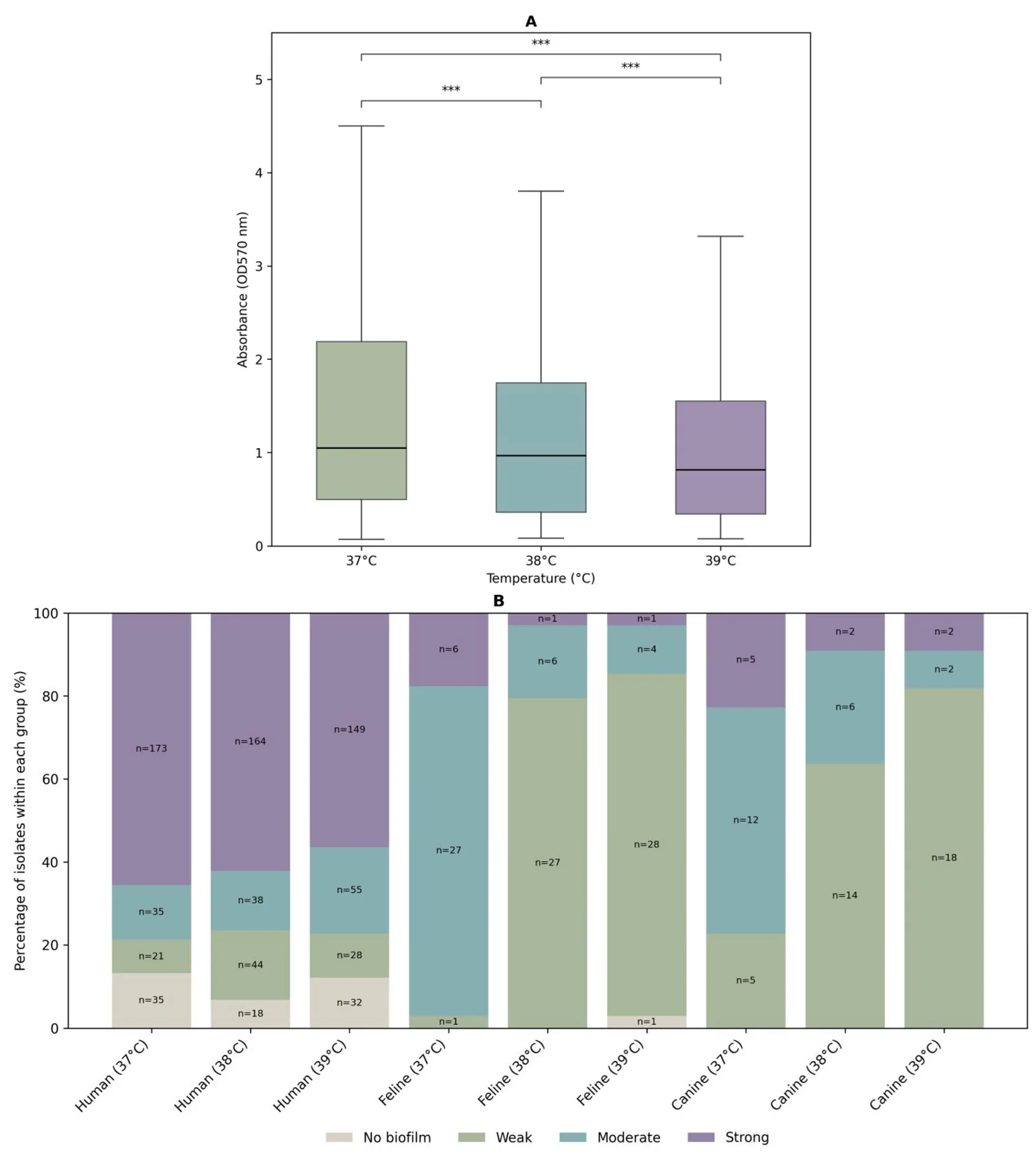
(**A**) Quantitative biofilm production by *Staphylococcus epidermidis* isolates after 24 h of incubation at 37 °C, 38 °C, and 39 °C, expressed as OD_570_ values. Statistical significance was assessed using a linear mixed-effects model with Holm-adjusted pairwise comparisons (*** *p* < 0.001; ns, not significant). (**B**) Distribution of biofilm-forming capacity categories (no biofilm, weak, moderate, and strong) among *S. epidermidis* isolates according to host origin (human, feline, and canine) and incubation temperature (37 °C, 38 °C, and 39 °C). Stacked bars represent the percentage of isolates within each host–temperature group, and the corresponding isolate counts (*n*) are shown within individual categories. The “no biofilm” category denotes isolates with OD_570_ values at or below the predefined ODc threshold and should be interpreted as no detectable biofilm production above the assay threshold under the tested conditions, rather than as a complete absence of biofilm-forming capacity.

**Figure 5:**
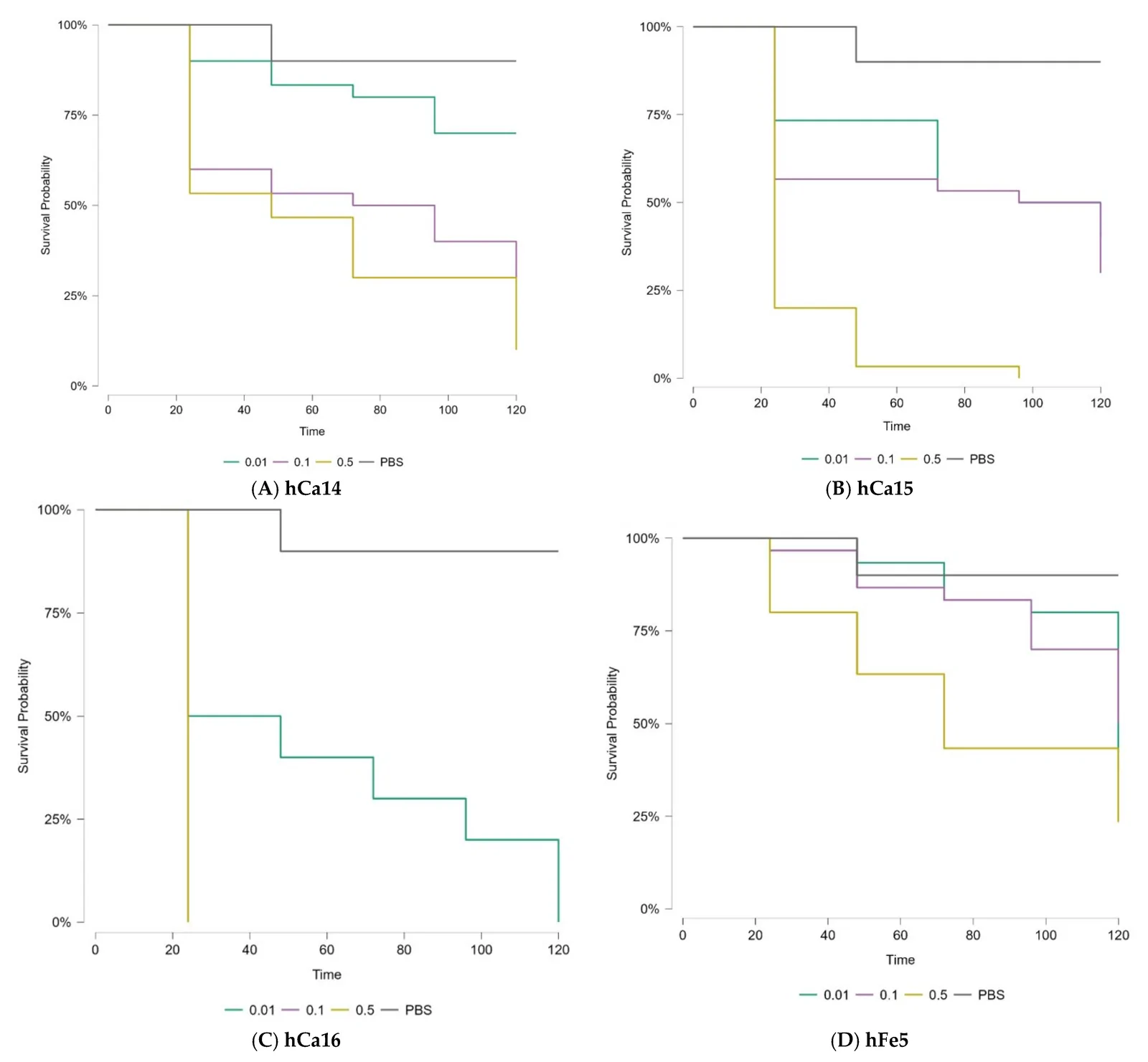

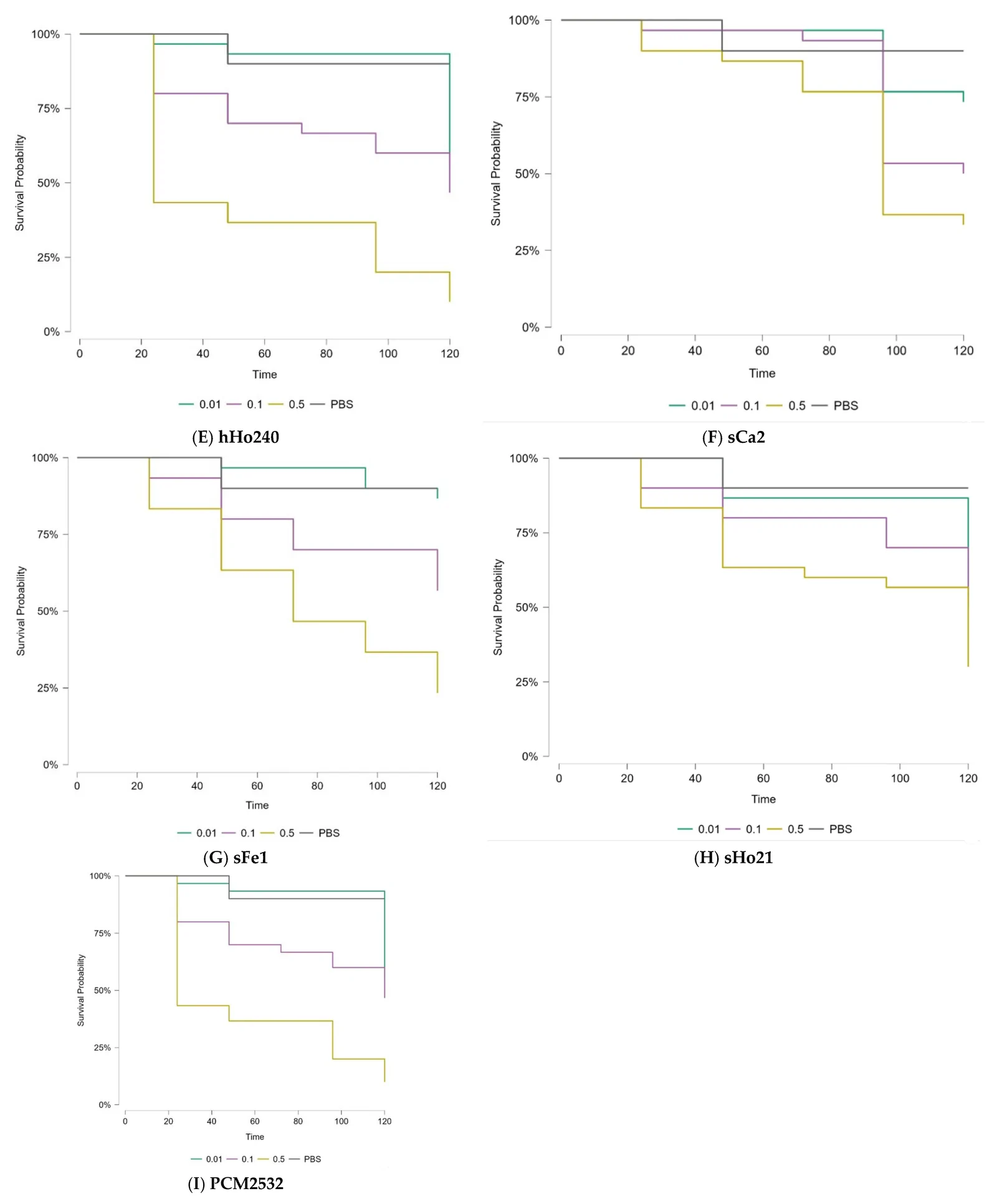
Kaplan–Meier survival analysis of *Galleria mellonella* larvae infected with selected *Staphylococcus epidermidis* isolates and the reference strain. (**A**) hCa14; (**B**) hCa15; (**C**) hCa16; (**D**) hFe5; (**E**) hHo240; (**F**) sCa2; (**G**) sFe1; (**H**) sHo21; (**I**) *S. epidermidis* PCM 2532. Survival was monitored for 120 h following infection with inocula corresponding to OD_600_ = 0.01, 0.1, and 0.5.

**Table 1:** Resistance (%) to selected antibiotics in *S. epidermidis* strains from humans and animals determined by the disk diffusion method.

Group	AMP	AMC	P	OX	SXT	MUP	FD	MAR	CIP	CN	TOB	RD	DA	E	C	TGC	LZD	TET
hHo (*n* = 243)	69/243 28.40%	33/243 13.58%	103/243 42.39%	171/243 70.37%	1/243 0.41%	13/243 5.35%	4/243 1.65%	4/243 1.65%	8/243 3.29%	3/243 1.23%	0/243 0%	0/243 0%	131/243 53.91%	168/243 69.14%	3/243 1.23%	0/243 0%	0/243 0%	135/243 55.56%
sHo (*n* = 21)	5/21 23.81%	2/21 9.52%	7/21 33.33%	12/21 57.14%	0/21 0%	0/21 0%	0/21 0%	0/21 0%	0/21 0%	0/21 0%	0/21 0%	0/21 0%	11/21 52.38%	9/21 42.86%	0/21 0%	0/21 0%	0/21 0%	5/21 23.81%
hFe (*n* = 32)	8/32 25.0%	1/32 3.13%	13/32 40.63%	17/32 53.13%	0/32 0%	1/32 3.13%	0/32 0%	1/32 3.13%	6/32 18.75%	2/32 6.25%	0/32 0%	0/32 0%	17/32 53.13%	26/32 81.25%	0/32 0%	0/32 0%	0/32 0%	23/32 71.88%
sFe (*n* = 2)	0/2 0%	0/2 0%	0/2 0%	2/2 100.0%	0/2 0%	0/2 0%	0/2 0%	0/2 0%	0/2 0%	0/2 0%	0/2 0%	0/2 0%	1/2 50.0%	1/2 50.0%	0/2 0%	0/2 0%	0/2 0%	2/2 100.0%
hCa (*n* = 18)	1/18 5.56%	0/18 0%	2/18 11.11%	9/18 50.00%	0/18 0%	0/18 0%	0/18 0%	0/18 0%	1/18 5.56%	0/18 0%	0/18 0%	0/18 0%	10/18 55.56%	7/18 38.89%	0/18 0%	0/18 0%	0/18 0%	10/18 55.56%
sCa (*n* = 4)	1/4 25.0%	0/4 0%	2/4 50.0%	4/4 100.0%	0/4 0%	0/4 0%	1/4 25.0%	0/4 0%	1/4 25.0%	1/4 25.0%	0/4 0%	0/4 0%	2/4 50.0%	2/4 50.0%	1/4 25.0%	0/4 0%	0/4 0%	3/4 75.0%
Total (*n* = 320)	84/320 26.25%	36/320 11.25%	127/320 39.69%	215/320 67.19%	1/320 0.31%	14/320 4.38%	5/320 1.56%	5/320 1.56%	16/320 5.0%	6/320 1.88%	0/320 0%	0/320 0%	172/320 53.75%	213/320 66.56%	4/320 1.25%	0/320 0%	0/320 0%	178/320 55.63%

AMP = ampicillin, AMC = amoxicillin/clavulanic acid, MUP = mupirocin, P = penicillin, FD = fusidic acid, MAR = marbofloxacin, CIP = ciprofloxacin, OX = oxacillin, LZD = linezolid, DA = clindamycin, E = erythromycin, C = chloramphenicol, RD = rifampicin, CN = gentamicin, SXT = trimethoprim/sulfamethoxazole, TGC = tigecycline, TET = tetracycline.

**Table 2:** Occurrence of selected antibiotic resistance genes among *S. epidermidis* strains from humans and animals (%).

Group	*blaZ*	*mecA*	*mecC*	*Aac* *	*tetM*	*tetO*	*tetK*	*tetL*	*ermA*	*ermB*	*ermC*	*fusB*	*vanA*	*vanB*	*mupA*
hHo (*n* = 243)	107/243 44.03%	32/243 13.17%	0/243 0%	4/243 1.65%	0/243 0%	5/243 2.06%	46/243 18.93%	3/243 1.23%	4/243 1.65%	0/243 0%	17/243 7%	23/243 9.47%	2/243 0.82%	0/243 0%	2/243 0.82%
sHo (*n* = 21)	16/21 76.19%	1/21 4.76%	0/21 0%	0/21 0%	0/21 0%	1/21 4.76%	4/21 19.05%	0/21 0%	0/21 0%	0/21 0%	2/21 9.52%	3/21 14.29%	1/21 4.76%	0/21 0%	1/21 4.76%
hFe (*n* = 32)	23/32 71.88%	14/32 43.75%	0/32 0%	6/32 18.75%	0/32 0%	0/32 0%	10/32 31.25%	0/32 0%	2/32 6.25%	0/32 0%	4/32 12.50%	0/32 0%	0/32 0%	0/32 0%	0/32 0%
sFe (*n* = 2)	2/2 100%	0/2 0%	0/2 0%	0/2 0%	0/2 0%	0/2 0%	0/2 0%	0/2 0%	0/2 0%	0/2 0%	0/2 0%	0/2 0%	0/2 0%	0/2 0%	0/2 0%
hCa (*n* = 18)	11/18 61.11%	4/18 22.22%	0/18 0%	1/18 5.56%	0/18 0%	5/18 27.78%	5/18 27.78%	0/18 0%	3/18 16.67%	0/18 0%	1/18 5.56%	0/18 0%	0/18 0%	0/18 0%	2/18 11.11%
sCa (*n* = 4)	3/4 75.0%	2/4 50.0%	0/4 0%	2/4 50%	0/4 0%	0/4 0%	1/4 25%	0/4 0%	0/4 0%	0/4 0%	0/4 0%	0/4 0%	0/4 0%	0/4 0%	0/4 0%
Total (*n* = 320)	162/320 50.63%	53/320 16.56%	0/320 0%	13/320 4.06%	0/320 0%	11/320 3.44%	66/320 20.63%	3/320 0.94%	9/320 2.81%	0/320 0%	24/320 7.50%	26/320 8.13%	3/320 0.94%	0/320 0%	5/320 1.56%

* *aac(6′)-Ie-aph(2″)-Ia.*

**Table 3:** Temperature-dependent growth patterns of selected *Staphylococcus epidermidis* isolates and their biofilm-forming phenotypes at 39 °C.

Growth Pattern	No. of Strains	Representative Isolates	Biofilm Phenotype of *S. epidermidis* Isolates at 39 °C *
Gradual decrease	2	**hFe5**, *S. aureus* ATCC 43300	Weak (1/1)
Gradual increase	3	**hHo238**, **sHo21**, **hCa16**	Weak (1/3), moderate (1/3), strong (1/3)
Peak at 38 °C	3	**hHo240**, **hHo242**, **sCa4**	Weak (3/3)
Decrease followed by recovery	5	**hHo236**, **hHo243**, **hCa13**, **hCa14**, **sCa1**	Weak (5/5)
Temperature-independent	4	**sFe1**, **sFe2**, **hCa12**, *S. epidermidis* PCM 1869	Weak (3/3)

* Biofilm phenotype at 39 °C refers only to the analysed *S. epidermidis* isolates; reference strains were included exclusively in the growth-kinetics analysis. Biofilm formation was classified as no biofilm, weak, moderate, or strong based on the crystal violet assay. Colour coding indicates host species and clinical status: black—clinically healthy humans; red—clinically affected humans; green—clinically healthy cats; blue—clinically affected cats; orange—clinically healthy dogs; pink—clinically affected dogs. Bold indicates study isolates; reference strains are shown in regular type.

**Table 4:** Kaplan–Meier survival analysis of *Galleria mellonella* larvae infected with selected *Staphylococcus epidermidis* isolates and the reference strain.

Isolate	Host	Status	χ^2^ (Log-Rank)	*p*-Value	LT_50_ at OD_600_ = 0.5 (h)	Survival at 120 h, OD_600_ = 0.5 (%)
hHo240	human	Healthy	39.34	<0.001	24	10.0
sHo21	human	Diseased	11.63	0.009	120	30.0
hCa14	dog	Healthy	31.68	<0.001	48	10.0
hCa15	dog	Healthy	48.53	<0.001	24	0.0
hCa16	dog	Healthy	75.06	<0.001	24	0.0
hFe5	cat	Healthy	16.78	<0.001	72	23.3
sFe1	cat	Diseased	32.61	<0.001	72	23.3
sCa2	dog	Diseased	15.75	0.001	96	33.3
PCM 2532	Reference strain	–	39.34	<0.001	24	10.0

LT_50_ represents the median survival time at the highest bacterial inoculum (OD_600_ = 0.5). The log-rank (Mantel–Haenszel) test compared survival distributions among the three bacterial inoculum concentrations and the PBS control.

**Table 5:** Distribution of *Staphylococcus epidermidis* isolates according to host species, clinical status, sampling site, and number of individual hosts.

Host Species	Clinical Status	Isolates, *n*	Individuals, *n*
Human	Clinically healthy	243	175
Human	Clinically affected	21	16
**Human total**		**264**	**191**
Cat	Clinically healthy	32	31
Cat	Clinically affected	2	2
**Cat total**		**34**	**33**
Dog	Clinically healthy	18	15
Dog	Clinically affected	4	4
Dog total		22	19
**Total**		**320**	**243**

Bold indicates subtotal and overall total values.

## Data Availability

The original contributions presented in this study are included in the article/Supplementary Material. Further inquiries can be directed to the corresponding author(s).

## Supplementary Materials

The following supporting information can be downloaded at: https://www.mdpi.com/article/10.3390/ijms27198585/s1.
