# Supplementary material for: Characteristics of Staphylococcus epidermidis Isolated from Humans and Animals
Source: Int J Mol Sci. 2026 Sep 25;27(19):8585. doi: 10.3390/ijms27198585 (PMC13665364; doi:10.3390/ijms27198585)
Supplement: Supplementary file 1 [file ijms-27-08585-s001.zip › ijms-4576749-Supplementary-Material.pdf]

Figure S1. Hierarchical clustering of MALDI-TOF MS spectral profiles of *Staphylococcus epidermidis* isolates. The dendrogram shows hierarchical clustering of all 320 study isolates together with the *S. epidermidis* reference strain based on MALDI-TOF MS spectral profiles. Cluster membership is detailed in legend.

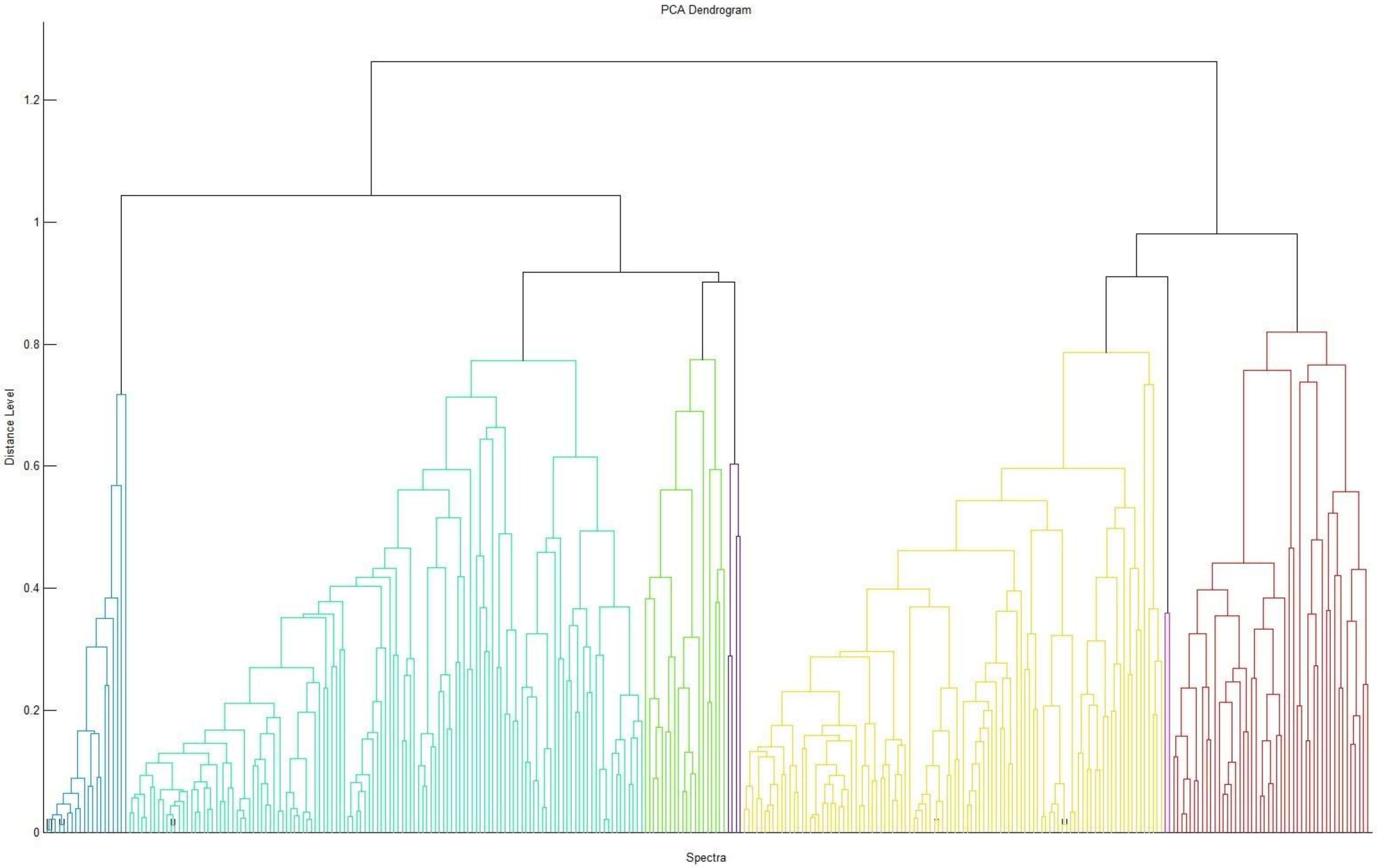

Table S1. the detailed distribution of *Staphylococcus epidermidis* isolates across the identified MALDI-TOF MS clusters. .

Cluster	No. of strains	Strains
Blue	20	hHo221, hCa4, hHo123, sHo12, hHo219, hHo173, hHo184, hHo86, hHo134, hHo93, hHo117, hHo208, hHo180, hHo181, hHo5, hHo113, hHo108, hHo9, hCa17, hHo49
Dark green	125	hHo38, hHo47, hHo15, hHo43, hHo50, hHo42, hHo53, hHo22, hHo32, hHo37, sHo7, hHo64, hHo56, hHo60, hHo44, hHo35, hHo57, hHo68, hHo45, hHo52, hHo67, sHo5, sHo4, hHo41, hHo40, hHo54, hHo55, hHo58, hHo74, hHo65, hHo8, hHo186, hHo30, hHo33, hHo24, hHo34, hHo197, hHo217, hHo3, hHo7, hHo16, hHo17, hHo10, hHo13, hHo18, hHo6, hHo4, hCa18, hFe13, hHo25, hHo31, hHo162, hHo200, hHo72, hHo168, hHo112, hFe18, hFe25, hHo87, sHo10, hHo165, hHo111, hFe23, sHo14, hHo20, hHo21, hHo39, sHo8, hHo135, hHo62, hHo2, hHo61, hHo73, hHo14, hHo66, hHo28, hHo63, hHo80, hHo124, hHo36, hHo48, hHo109, hHo121, hHo185, hHo19, hHo23, sHo2, hHo26, hHo59, hHo27, hHo114, sHo3, hHo107, hHo99, hHo106, hHo78, hHo183, hHo187, hHo193, hCa1, hHo155, sHo19, hHo195, hHo209, hHo119, hHo159, hHo152, hHo216, hHo199, hHo204, hHo202, hHo203, hHo226, hHo167, hHo170, hHo178, hHo211, hHo172, hHo218, hHo222, hHo182, sHo20, hHo196, hHo194, hHo215
Light green	20	sHo1, hHo104, hHo105, hHo132, hHo125, hHo91, hHo136, hHo118, hHo11, hHo76, hHo213, hHo116, hHo192, hHo163, hCa3, hHo88, hHo126, hHo137, hHo150, hHo146
Purple	4	hHo70, hHo79, hFe22, hCa13
Yellow	102	Referential strain + hHo235, hFe24, hHo242, hCa11, hFe4, hFe5, hFe30, hFe26, hHo115, hFe9, hHo232, sHo21, hCa6, sHo6, hHo174, hHo127, hHo143, hHo138, hHo133, hHo238, hHo231, hFe29, hFe1, sFe2, sCa3, hFe14, sHo11, hHo156, hHo169, hHo158, hHo175, hHo176, hHo51, hHo75, hFe3, hHo110, hHo130, hHo198, hHo240, hHo189, hHo243, hFe31, hFe28, hCa12, hFe6, hFe2, hFe27, sCa4, hCa9, hFe32, hFe17, hCa15, hHo12, sHo9, hCa16, hFe19, hCa8, hCa5, hHo92, hCa14, sHo17, hHo147, sFe1, hHo234, hCa2, hHo122, hHo157, hHo84, hHo128, hHo141, hHo153, hHo166, hHo177, hHo227, hHo225, hHo224, hHo229, hHo210, hHo230, hHo69, hHo142, hHo89, hHo151, hHo90, sHo15, hHo179, hHo228, hHo102, hHo145, hHo103, hHo171, hHo81, hHo100, sHo18, hFe10, hHo46, sHo16, hHo206, hFe16, hFe21, hFe11
Pink	2	hHo29, hHo96
Red	48	hHo71, hHo120, hHo77, hHo149, hHo131, hHo95, hHo129, hHo83, hHo97, hHo98, hHo94, hHo1, hHo236, sCa2, hHo239, hFe7, hHo233, hHo154, hHo201, hHo82, hHo101, hHo85, sHo13, hHo139, hHo144, hHo140, sCa1, hHo214, hHo148, hHo237, hHo160, hHo161, hHo188, hHo190, hHo212, hHo220, hHo223, hHo164, hFe20, hCa7, hFe8, hFe15, hHo191, hHo241, hFe12, hCa10, hHo205, hHo207

Table S2. *Staphylococcus epidermidis* isolates and reference sequences included in the partial *rpoB* gene phylogenetic analysis. GenBank accession numbers for the analysed sequences will be provided upon finalization of sequence deposition.

No.	Study isolate ID	Host	Clinical status	Sampling site	GenBank accession number
1.	**hHo7**	Human	Clinically healthy	Oral cavity	QB047028
2.	**sHo3**	Human	Clinically affected	Skin	QB047029
3.	**sHo4**	Human	Clinically affected	Skin	QB047030
4.	**sHo8**	Human	Clinically affected	Skin	QB047031
5.	**hHo73**	Human	Clinically healthy	Skin	QB047032
6.	**hHo74**	Human	Clinically healthy	Skin	QB047033
7.	**sHo9**	Human	Clinically affected	Skin	QB047034
8.	**hHo81**	Human	Clinically healthy	Skin	QB047035
9.	**hHo93**	Human	Clinically healthy	Nostrils	QB047036
10.	**hHo97**	Human	Clinically healthy	Nostrils	QB047037
11.	**hHo105**	Human	Clinically healthy	Nostrils	QB047038
12.	**hHo108**	Human	Clinically healthy	Nostrils	QB047092
13.	**sHo15**	Human	Clinically affected	Nostrils	QB047039
14.	**hHo143**	Human	Clinically healthy	Nostrils	QB047040
15.	**hHo144**	Human	Clinically healthy	Nostrils	QB047041
16.	**sHo18**	Human	Clinically affected	Skin	QB047042
17.	**hHo151**	Human	Clinically healthy	Skin	QB047043
18.	**hHo158**	Human	Clinically healthy	Ear	QB047044
19.	**hHo173**	Human	Clinically healthy	Ear	QB047045
20.	**sHo19**	Human	Clinically affected	Ear	QB047046
21.	**hHo183**	Human	Clinically healthy	Ear	QB047047
22.	**hHo202**	Human	Clinically healthy	Ear	QB047048
23.	**hHo210**	Human	Clinically healthy	Ear	QB047049
24.	**hHo224**	Human	Clinically healthy	Ear	QB047050
25.	**hHo234**	Human	Clinically healthy	Ear	QB047051
26.	**hHo242**	Human	Clinically healthy	Ear	QB047052
27.	**hCa1**	Dog	Clinically healthy	Oral cavity	QB047053
28.	**hCa3**	Dog	Clinically healthy	Oral cavity	QB047054
29.	**hCa4**	Dog	Clinically healthy	Oral cavity	QB047055
30.	**hFe1**	Cat	Clinically healthy	Nostrils	QB047056
31.	**hFe2**	Cat	Clinically healthy	Nostrils	Pending
32.	**hFe3**	Cat	Clinically healthy	Nostrils	QB047057
33.	**hFe4**	Cat	Clinically healthy	Nostrils	QB047058
34.	**hCa16**	Dog	Clinically healthy	Nostrils	QB047059
35.	**hCa5**	Dog	Clinically healthy	Nostrils	QB047060
36.	**hCa8**	Dog	Clinically healthy	Nostrils	QB047088
37.	**hFe14**	Cat	Clinically healthy	Conjunctival sac	QB047066
38.	**hFe6**	Cat	Clinically healthy	Conjunctival sac	QB047061
39.	**hFe9**	Cat	Clinically healthy	Conjunctival sac	QB047062
40.	**hFe10**	Cat	Clinically healthy	Conjunctival sac	QB047063
41.	**hFe11**	Cat	Clinically healthy	Conjunctival sac	QB047064
42.	**hFe13**	Cat	Clinically healthy	Conjunctival sac	QB047065
43.	**hFe15**	Cat	Clinically healthy	Conjunctival sac	QB047067
44.	**hFe16**	Cat	Clinically healthy	Conjunctival sac	QB047068
45.	**hCa9**	Dog	Clinically healthy	Conjunctival sac	QB047069
46.	**hCa10**	Dog	Clinically healthy	Conjunctival sac	QB047070
47.	**hCa11**	Dog	Clinically healthy	Conjunctival sac	QB047074
48.	**sCa1**	Dog	Clinically affected	Conjunctival sac	QB047071
49.	**sCa2**	Dog	Clinically affected	Conjunctival sac	QB047072
50.	**sCa3**	Dog	Clinically affected	Conjunctival sac	QB047073
51.	**hFe17**	Cat	Clinically healthy	Anus	QB047075
52.	**hFe19**	Cat	Clinically healthy	Anus	QB047089
53.	**hFe23**	Cat	Clinically healthy	Skin	QB047078
54.	**hFe21**	Cat	Clinically healthy	Skin	QB047076
55.	**hFe22**	Cat	Clinically healthy	Skin	QB047077
56.	**hFe25**	Cat	Clinically healthy	Skin	QB047079
57.	**hFe26**	Cat	Clinically healthy	Skin	QB047080
58.	**hFe28**	Cat	Clinically healthy	Skin	QB047081
59.	**sFe2**	Cat	Clinically affected	Skin	QB047082
60.	**hCa12**	Dog	Clinically healthy	Skin	QB047083
61.	**hCa13**	Dog	Clinically healthy	Skin	QB047090

**62.**	**hCa14**	Dog	Clinically healthy	Skin	QB047091
**63.**	**sFe1**	Cat	Clinically affected	Skin	QB047084
**64.**	**hFe30**	Cat	Clinically healthy	Ear	QB047085
**65.**	**hFe31**	Cat	Clinically healthy	Ear	QB047086
**66.**	**hCa15**	Dog	Clinically healthy	Ear	Pending
**67.**	**sCa4**	Dog	Clinically affected	Ear	QB047087

Table S3. Genome assembly quality metrics and analysis status of the 67 *Staphylococcus epidermidis* isolates subjected to whole-genome sequencing.

Study isolate ID	Assembly length (Mb)	No. of contigs	Completeness (%)	Contamination (%)	Analysis category
**sHo4**	2.558	2	99.99	0.06	AMR/concordance + HQ phylogenetic backbone
**hHo81**	2.526	4	99.98	0.05	AMR/concordance + HQ phylogenetic backbone
**hHo105**	2.530	2	99.99	0.04	AMR/concordance + HQ phylogenetic backbone
**sHo15**	2.514	3	100.00	0.04	AMR/concordance + HQ phylogenetic backbone
**hHo151**	2.615	3	99.99	0.28	AMR/concordance + HQ phylogenetic backbone
**hHo158**	2.455	4	100.00	0.52	AMR/concordance + HQ phylogenetic backbone
**hHo183**	2.601	4	100.00	0.21	AMR/concordance + HQ phylogenetic backbone
**hHo202**	2.557	2	100.00	0.17	AMR/concordance + HQ phylogenetic backbone
**hHo210**	2.531	5	100.00	0.06	AMR/concordance + HQ phylogenetic backbone
**hHo242**	2.477	72	87.60	1.36	Lower-quality phylogenetic placement only

**hHo73**	2.512	2	99.97	0.03	AMR/concordance + HQ phylogenetic backbone
**hHo7**	2.573	6	100.00	0.74	AMR/concordance + HQ phylogenetic backbone
**sHo3**	2.503	2	99.98	0.04	AMR/concordance + HQ phylogenetic backbone
**sHo8**	2.516	3	99.99	0.05	AMR/concordance + HQ phylogenetic backbone
**hHo74**	2.524	3	99.99	0.03	AMR/concordance + HQ phylogenetic backbone
**sHo9**	2.501	7	99.96	0.20	AMR/concordance + HQ phylogenetic backbone
**hHo93**	2.495	4	99.99	0.03	AMR/concordance + HQ phylogenetic backbone
**hHo97**	2.628	6	99.99	0.13	AMR/concordance + HQ phylogenetic backbone
**hHo108**	2.520	7	99.99	0.19	AMR/concordance + HQ phylogenetic backbone
**hHo143**	2.534	5	100.00	0.73	AMR/concordance + HQ phylogenetic backbone
**hHo144**	2.554	2	99.98	0.04	AMR/concordance + HQ phylogenetic backbone
**sHo18**	2.429	24	97.41	0.88	AMR/concordance + lower-quality phylogenetic placement

**hHo173**	2.539	5	99.98	0.26	AMR/concordance + HQ phylogenetic backbone
**sHo19**	2.496	2	99.98	0.05	AMR/concordance + HQ phylogenetic backbone
**hHo224**	2.538	5	99.99	0.10	AMR/concordance + HQ phylogenetic backbone
**hHo234**	2.512	2	99.97	0.02	AMR/concordance + HQ phylogenetic backbone
**hCa1**	1.911	62	63.49	0.33	Lower-quality phylogenetic placement only
**hCa3**	2.582	4	100.00	0.10	AMR/concordance + HQ phylogenetic backbone
**hCa4**	2.513	8	99.99	0.03	AMR/concordance + HQ phylogenetic backbone
**hFe1**	2.597	9	100.00	0.05	AMR/concordance + HQ phylogenetic backbone
**hFe2**	2.077	83	68.94	2.59	Lower-quality phylogenetic placement only
**hFe3**	1.910	128	70.55	0.13	Lower-quality phylogenetic placement only
**hFe4**	2.513	3	100.00	0.07	AMR/concordance + HQ phylogenetic backbone
**hCa16**	2.532	1	99.99	0.17	AMR/concordance + HQ phylogenetic backbone

**hCa5**	1.362	131	52.72	2.64	Lower-quality phylogenetic placement only
**hCa8**	2.544	3	99.99	0.10	AMR/concordance + HQ phylogenetic backbone
**hFe6**	2.493	3	100.00	0.05	AMR/concordance + HQ phylogenetic backbone
**hFe9**	2.493	3	100.00	0.05	AMR/concordance + HQ phylogenetic backbone
**hFe10**	2.534	5	99.99	0.74	AMR/concordance + HQ phylogenetic backbone
**hFe11**	2.631	32	99.93	0.45	AMR/concordance + HQ phylogenetic backbone
**hFe13**	2.487	3	99.99	0.10	AMR/concordance + HQ phylogenetic backbone
**hFe14**	2.611	7	100.00	0.60	AMR/concordance + HQ phylogenetic backbone
**hFe15**	2.555	6	99.99	0.14	AMR/concordance + HQ phylogenetic backbone
**hFe16**	1.978	104	65.58	0.46	Lower-quality phylogenetic placement only
**hCa9**	2.546	5	99.97	0.42	AMR/concordance + HQ phylogenetic backbone
**hCa10**	2.636	11	99.99	0.07	AMR/concordance + HQ phylogenetic backbone

**sCa1**	2.665	4	99.98	0.46	AMR/concordance + HQ phylogenetic backbone
**sCa2**	2.568	4	99.99	0.80	AMR/concordance + HQ phylogenetic backbone
**sCa3**	2.536	4	99.99	0.60	AMR/concordance + HQ phylogenetic backbone
**hCa11**	2.454	7	99.84	0.03	AMR/concordance + HQ phylogenetic backbone
**hFe17**	2.532	1	99.99	0.17	AMR/concordance + HQ phylogenetic backbone
**hFe19**	2.199	93	90.15	0.80	Lower-quality phylogenetic placement only
**hFe21**	2.460	5	99.99	0.06	AMR/concordance + HQ phylogenetic backbone
**hFe22**	1.778	59	55.84	1.57	Lower-quality phylogenetic placement only
**hFe23**	1.080	87	35.82	2.96	Excluded
**hFe25**	2.464	6	99.99	0.03	AMR/concordance + HQ phylogenetic backbone
**hFe26**	2.351	67	89.21	1.54	Lower-quality phylogenetic placement only
**hFe28**	2.466	7	99.99	0.03	AMR/concordance + HQ phylogenetic backbone
**sFe2**	2.467	6	99.99	0.03	AMR/concordance + HQ phylogenetic backbone

**hCa12**	2.490	3	100.00	0.09	AMR/concordance + HQ phylogenetic backbone
**hCa13**	2.477	3	100.00	0.21	AMR/concordance + HQ phylogenetic backbone
**hCa14**	2.529	22	100.00	0.15	AMR/concordance + HQ phylogenetic backbone
**sFe1**	2.522	2	99.97	0.02	AMR/concordance + HQ phylogenetic backbone
**hFe30**	1.277	150	53.87	3.84	Lower-quality phylogenetic placement only
**hFe31**	2.490	2	99.98	0.02	AMR/concordance + HQ phylogenetic backbone
**hCa15**	1.660	89	51.68	4.35	Lower-quality phylogenetic placement only
**sCa4**	2.574	4	99.99	0.73	AMR/concordance + HQ phylogenetic backbone

Note: For WGS-based antimicrobial resistance and genotype–phenotype concordance analyses, assemblies were required to have a total length of 2.2–2.8 Mb, completeness ≥95%, contamination ≤5%, and fewer than 200 contigs. Assemblies used for the high-quality phylogenetic backbone had completeness ≥99% and contamination ≤3%. Lower-quality assemblies with completeness ≥50% and contamination <5% were eligible for phylogenetic placement.

Table S4. Multilocus sequence typing results for 67 *Staphylococcus epidermidis* isolates, including sequence type assignment, host origin, clinical status, and sampling site.

Study isolate ID	Host	Clinical status	Sampling site	ST	MLST assignment
**hHo7**	Human	Clinically healthy	Oral cavity	—	Ambiguous allele assignment
**sHo3**	Human	Clinically affected	Skin	986	Known ST assigned
**sHo4**	Human	Clinically affected	Skin	7	Known ST assigned
**sHo8**	Human	Clinically affected	Skin	87	Known ST assigned
**hHo73**	Human	Clinically healthy	Skin	19	Known ST assigned
**hHo74**	Human	Clinically healthy	Skin	87	Known ST assigned
**sHo9**	Human	Clinically affected	Skin	54	Known ST assigned
**hHo81**	Human	Clinically healthy	Skin	88	Known ST assigned
**hHo93**	Human	Clinically healthy	Nostrils	153	Known ST assigned
**hHo97**	Human	Clinically healthy	Nostrils	81	Known ST assigned
**hHo105**	Human	Clinically healthy	Nostrils	297	Known ST assigned
**hHo108**	Human	Clinically healthy	Nostrils	17	Known ST assigned
**sHo15**	Human	Clinically affected	Nostrils	297	Known ST assigned
**hHo143**	Human	Clinically healthy	Nostrils	—	Ambiguous allele assignment
**hHo144**	Human	Clinically healthy	Nostrils	190	Known ST assigned
**sHo18**	Human	Clinically affected	Skin	—	Ambiguous allele assignment
**hHo151**	Human	Clinically healthy	Skin	153	Known ST assigned
**hHo158**	Human	Clinically healthy	Ear	57	Known ST assigned
**hHo173**	Human	Clinically healthy	Ear	228	Known ST assigned
**sHo19**	Human	Clinically affected	Ear	—	Ambiguous allele assignment
**hHo183**	Human	Clinically healthy	Ear	59	Known ST assigned
**hHo202**	Human	Clinically healthy	Ear	35	Known ST assigned
**hHo210**	Human	Clinically healthy	Ear	297	Known ST assigned
**hHo224**	Human	Clinically healthy	Ear	153	Known ST assigned
**hHo234**	Human	Clinically healthy	Ear	19	Known ST assigned
**hHo242**	Human	Clinically healthy	Ear	32	Known ST assigned
**hCa1**	Dog	Clinically healthy	Oral cavity	—	Ambiguous allele assignment
**hCa3**	Dog	Clinically healthy	Oral cavity	1146	Known ST assigned
**hCa4**	Dog	Clinically healthy	Oral cavity	153	Known ST assigned
**hFe1**	Cat	Clinically healthy	Nostrils	210	Known ST assigned
**hFe2**	Cat	Clinically healthy	Nostrils	—	Incomplete profile
**hFe3**	Cat	Clinically healthy	Nostrils	—	Incomplete profile
**hFe4**	Cat	Clinically healthy	Nostrils	32	Known ST assigned
**hCa16**	Dog	Clinically healthy	Nostrils	87	Known ST assigned
**hCa5**	Dog	Clinically healthy	Nostrils	—	Incomplete profile
**hCa8**	Dog	Clinically healthy	Nostrils	764	Known ST assigned
**hFe14**	Cat	Clinically healthy	Conjunctival sac	5	Known ST assigned
**hFe6**	Cat	Clinically healthy	Conjunctival sac	210	Known ST assigned
**hFe9**	Cat	Clinically healthy	Conjunctival sac	210	Known ST assigned
**hFe10**	Cat	Clinically healthy	Conjunctival sac	5	Known ST assigned
**hFe11**	Cat	Clinically healthy	Conjunctival sac	—	Complete profile, no known ST assignment
**hFe13**	Cat	Clinically healthy	Conjunctival sac	73	Known ST assigned
**hFe15**	Cat	Clinically healthy	Conjunctival sac	720	Known ST assigned

hFe16	Cat	Clinically healthy	Conjunctival sac	—	Incomplete profile
hCa9	Dog	Clinically healthy	Conjunctival sac	190	Known ST assigned
hCa10	Dog	Clinically healthy	Conjunctival sac	200	Known ST assigned
hCa11	Dog	Clinically healthy	Conjunctival sac	631	Known ST assigned
sCa1	Dog	Clinically affected	Conjunctival sac	190	Known ST assigned
sCa2	Dog	Clinically affected	Conjunctival sac	81	Known ST assigned
sCa3	Dog	Clinically affected	Conjunctival sac	5	Known ST assigned
hFe17	Cat	Clinically healthy	Anus	87	Known ST assigned
hFe19	Cat	Clinically healthy	Anus	—	Ambiguous allele assignment
hFe23	Cat	Clinically healthy	Skin	—	Incomplete profile
hFe21	Cat	Clinically healthy	Skin	35	Known ST assigned
hFe22	Cat	Clinically healthy	Skin	—	Incomplete profile
hFe25	Cat	Clinically healthy	Skin	153	Known ST assigned
hFe26	Cat	Clinically healthy	Skin	—	Incomplete profile
hFe28	Cat	Clinically healthy	Skin	153	Known ST assigned
sFe2	Cat	Clinically affected	Skin	153	Known ST assigned
hCa12	Dog	Clinically healthy	Skin	1242	Known ST assigned
hCa13	Dog	Clinically healthy	Skin	—	Complete profile, no known ST assignment
hCa14	Dog	Clinically healthy	Skin	—	Complete profile, no known ST assignment
sFe1	Cat	Clinically affected	Skin	19	Known ST assigned
hFe30	Cat	Clinically healthy	Ear	—	Incomplete profile
hFe31	Cat	Clinically healthy	Ear	88	Known ST assigned
hCa15	Dog	Clinically healthy	Ear	—	Incomplete profile
sCa4	Dog	Clinically affected	Ear	384	Known ST assigned

Table S5. Phylogenetic inclusion status of *Staphylococcus epidermidis* isolates included in the core-genome phylogenetic analysis.

Study isolate ID	Phylogenetic inclusion status
sFe2	HQ backbone
hFe28	HQ backbone
hFe25	HQ backbone
hHo93	HQ backbone
hCa4	HQ backbone
hHo151	HQ backbone
hHo224	HQ backbone
hHo108	HQ backbone
sHo15	HQ backbone
hHo210	HQ backbone
hHo105	HQ backbone
hHo97	HQ backbone
sCa2	HQ backbone
hHo183	HQ backbone
hHo143	HQ backbone
hHo158	HQ backbone
sHo8	HQ backbone
hHo74	HQ backbone
hCa12	HQ backbone
hCa5	Lower-quality placement
hCa1	Lower-quality placement
hHo202	HQ backbone
hFe21	HQ backbone

**sHo9**	HQ backbone
**sHo4**	HQ backbone
**sHo19**	HQ backbone
**hFe2**	Lower-quality placement
**hFe26**	Lower-quality placement
**hFe6**	HQ backbone
**hFe9**	HQ backbone
**hFe1**	HQ backbone
**sCa4**	HQ backbone
**sHo18**	Lower-quality placement
**sCa1**	HQ backbone
**hHo144**	HQ backbone
**hCa9**	HQ backbone
**hCa15**	Lower-quality placement
**hFe30**	Lower-quality placement
**hFe31**	HQ backbone
**hHo81**	HQ backbone
**hCa11**	HQ backbone
**sHo3**	HQ backbone
**hFe13**	HQ backbone
**hHo173**	HQ backbone
**hFe11**	HQ backbone
**hFe3**	Lower-quality placement
**hFe10**	HQ backbone
**sCa3**	HQ backbone
**hFe14**	HQ backbone
**hHo7**	HQ backbone
**hCa16**	HQ backbone
**hFe17**	HQ backbone
**hFe15**	HQ backbone
**hHo234**	HQ backbone
**hHo73**	HQ backbone
**sFe1**	HQ backbone
**hFe16**	Lower-quality placement
**hFe22**	Lower-quality placement
**hHo242**	Lower-quality placement
**hFe4**	HQ backbone
**hCa10**	HQ backbone
**hFe19**	Lower-quality placement
**hCa3**	HQ backbone
**hCa13**	HQ backbone
**hCa14**	HQ backbone
**hCa8**	HQ backbone

Note: The maximum-likelihood phylogenetic backbone comprised 54 high-quality genome assemblies. Twelve lower-quality assemblies fulfilling the predefined placement criteria were subsequently placed onto the backbone using EPA-ng and gappa, resulting in a final phylogeny comprising 66 isolates.

Table S6. Distribution of antimicrobial resistance and resistance-associated genes identified by CARD among 55 *Staphylococcus epidermidis* genome assemblies.

Study isolate ID	*aac(6')-Ie-aph(2")-Ia*	*ANT(4')-Ia*	*ANT(9)-Ia*	*APH(3')-IIIa*	E. faecium chloramphenicol acetyltransferase	*ermA*	*ermC*	*FosD*	*FosY*	*PC1*	*blaZ*	*SAT-4*	*LmrS*
**sHo4**	0	0	0	0	0	0	0	1	0	0	1	0	1
**hHo81**	0	0	0	0	0	0	0	0	1	0	1	0	1
**hHo105**	0	0	0	0	0	0	0	0	1	0	0	0	1
**sHo15**	0	1	0	0	0	0	0	0	1	1	0	0	1
**hHo151**	1	0	0	0	0	0	0	0	1	0	1	0	1
**hHo158**	0	1	0	0	0	0	0	1	0	1	0	0	1
**hHo183**	0	0	0	0	0	0	0	0	1	0	0	0	1
**hHo202**	0	1	0	0	0	0	0	0	1	0	1	0	1
**hHo210**	0	1	0	0	0	0	1	0	1	1	0	0	1
**hHo73**	0	0	0	0	0	0	0	1	0	0	0	0	1
**hHo7**	1	1	0	0	0	0	1	1	0	1	1	0	1
**sHo3**	0	0	0	0	0	0	0	0	1	1	0	0	1
**sHo8**	0	0	0	0	0	0	0	0	1	0	1	0	1
**hHo74**	0	0	0	0	0	0	0	0	1	0	1	0	1
**sHo9**	0	0	0	0	0	0	0	1	0	0	1	0	1
**hHo93**	0	0	0	0	0	0	0	0	1	0	1	0	1
**hHo97**	1	0	0	0	0	0	0	0	1	1	0	0	1
**hHo108**	0	0	0	1	0	0	0	1	0	0	1	1	1
**hHo143**	0	0	0	0	0	0	0	0	1	1	0	0	1
**hHo144**	0	0	0	0	0	0	0	1	0	0	0	0	1
**sHo18**	0	0	0	0	0	0	0	1	0	0	1	0	1
**hHo173**	0	1	1	0	0	1	0	0	1	0	1	0	1
**sHo19**	0	0	0	0	0	0	0	1	0	0	1	0	1
**hHo224**	1	0	0	0	0	0	0	0	1	0	1	0	1
**hHo234**	0	0	0	0	0	0	0	1	0	0	0	0	1
**hCa3**	0	0	0	0	0	0	0	0	1	1	0	0	1
**hCa4**	0	0	0	0	0	0	0	0	1	0	1	0	1
**hFe1**	1	1	0	0	0	0	1	1	0	0	0	0	1
**hFe4**	0	0	0	0	0	0	0	1	0	1	0	0	1
**hCa16**	1	0	1	0	0	1	0	0	1	0	1	0	1
**hCa8**	0	0	0	0	0	0	0	0	1	0	0	0	1

**hFe6**	1	1	0	0	0	0	1	1	0	0	1	0	1
**hFe9**	1	1	0	0	0	0	1	1	0	0	1	0	1
**hFe10**	0	0	0	0	0	0	1	1	0	0	1	0	1
**hFe11**	0	0	0	0	0	0	0	1	0	0	1	0	1
**hFe13**	0	0	0	0	0	0	0	0	1	0	1	0	1
**hFe14**	0	0	0	0	0	0	1	1	0	0	1	0	1
**hFe15**	0	0	0	0	0	0	0	1	0	0	1	0	1
**hCa9**	0	0	0	0	0	0	0	1	0	1	0	0	1
**hCa10**	0	0	0	0	0	0	1	1	0	0	1	0	1
**sCa1**	1	0	0	1	0	0	0	1	0	0	0	1	1
**sCa2**	1	0	0	0	0	0	0	0	1	1	0	0	1
**sCa3**	0	0	0	0	0	0	1	1	0	0	1	0	1
**hCa11**	0	0	0	0	0	0	0	0	1	0	1	0	1
**hFe17**	1	0	1	0	0	1	0	0	1	0	1	0	1
**hFe21**	0	0	0	0	0	0	0	0	1	0	1	0	1
**hFe25**	0	0	0	0	0	0	0	0	1	0	1	0	1
**hFe28**	0	0	0	0	0	0	0	0	1	0	1	0	1
**sFe2**	0	0	0	0	0	0	0	0	1	0	1	0	1
**hCa12**	0	0	0	0	0	0	0	1	0	0	1	0	1
**hCa13**	0	0	0	0	0	0	0	1	0	0	0	0	1
**hCa14**	0	0	0	0	0	0	1	0	1	0	1	0	1
**sFe1**	0	0	0	0	0	0	0	1	0	0	1	0	1
**hFe31**	0	0	0	0	0	0	0	1	0	0	1	0	1
**sCa4**	0	0	0	0	0	0	1	1	0	0	1	0	1

**Study isolate ID**	*mupA*	**S. intermedius chloramphenicol acetyltransferase**	*aad(6)*	*arlR*	*arlS*	*dfrC*	*dfrG*	*fusB*	*lnuA*	*mecA*	*mecI*	*mecR1*	*mgrA*
**sHo4**	0	0	0	1	1	1	0	0	0	0	0	0	1
**hHo81**	0	0	0	1	1	1	0	0	0	0	0	0	1
**hHo105**	0	0	0	1	1	1	0	0	0	0	0	0	1
**sHo15**	0	0	0	1	1	1	0	0	0	0	0	0	1
**hHo151**	0	0	0	1	1	1	0	1	0	0	0	0	1
**hHo158**	1	0	0	1	1	1	0	0	0	1	0	0	1
**hHo183**	0	0	0	1	1	1	0	0	0	0	0	0	1
**hHo202**	0	0	0	1	1	1	0	0	0	1	0	0	1
**hHo210**	0	0	0	1	1	1	0	0	0	0	0	0	1
**hHo73**	0	0	0	1	1	1	0	0	0	0	0	0	1
**hHo7**	1	0	0	1	1	1	0	1	0	1	0	0	1
**sHo3**	0	0	0	1	1	1	0	0	0	0	0	0	1
**sHo8**	0	0	0	1	1	1	0	0	0	0	0	0	1
**hHo74**	0	0	0	1	1	1	0	0	0	0	0	0	1
**sHo9**	0	0	0	1	1	1	0	0	0	1	0	0	1
**hHo93**	0	0	0	1	1	1	0	0	0	0	0	0	1
**hHo97**	0	0	0	1	1	1	0	0	0	1	0	0	1
**hHo108**	0	0	1	1	1	1	0	0	0	1	0	0	1
**hHo143**	1	1	0	1	1	1	0	0	0	1	0	0	1
**hHo144**	0	0	0	1	1	1	0	0	0	0	0	0	1
**sHo18**	0	0	0	1	1	1	0	0	0	0	0	0	1
**hHo173**	0	0	0	1	1	1	0	0	0	0	0	0	1
**sHo19**	0	0	0	1	1	1	0	1	0	0	0	0	1
**hHo224**	0	0	0	1	1	1	0	0	0	0	0	0	1
**hHo234**	0	0	0	1	1	1	0	0	0	0	0	0	1
**hCa3**	0	0	0	1	1	1	0	0	0	0	0	0	1
**hCa4**	0	0	0	1	1	1	0	0	0	0	0	0	1
**hFe1**	0	0	0	1	1	1	1	0	1	1	0	0	1
**hFe4**	0	0	0	1	1	1	0	0	0	0	0	0	1
**hCa16**	0	0	0	1	1	1	0	1	0	1	0	0	1
**hCa8**	0	0	0	1	1	1	0	0	0	0	0	0	1
**hFe6**	0	0	0	1	1	1	1	0	1	1	0	0	1
**hFe9**	0	0	0	1	1	1	1	0	1	1	0	0	1
**hFe10**	1	0	0	1	1	1	0	0	0	1	0	0	1
**hFe11**	0	0	0	1	1	1	0	0	0	0	0	0	1
**hFe13**	0	0	0	1	1	1	0	0	0	0	0	0	1
**hFe14**	1	0	0	1	1	1	0	0	0	1	0	0	1
**hFe15**	0	0	0	1	1	1	0	0	0	0	0	0	1
**hCa9**	0	1	0	1	1	1	0	0	0	0	0	0	1
**hCa10**	0	0	0	1	1	1	0	0	0	0	0	0	1
**sCa1**	0	0	1	1	1	1	0	0	0	0	0	0	1
**sCa2**	0	0	0	1	1	1	0	0	0	1	0	0	1
**sCa3**	1	0	0	1	1	1	0	0	0	1	0	0	1

**hCa11**	0	0	0	1	1	1	0	0	0	0	0	0	1
**hFe17**	0	0	0	1	1	1	0	1	0	1	0	0	1
**hFe21**	0	0	0	1	1	1	0	0	1	1	0	0	1
**hFe25**	0	0	0	1	1	1	0	0	0	0	0	0	1
**hFe28**	0	0	0	1	1	1	0	0	0	0	0	0	1
**sFe2**	0	0	0	1	1	1	0	0	0	0	0	0	1
**hCa12**	0	0	0	1	1	1	0	0	0	0	0	0	1
**hCa13**	0	0	0	1	1	1	0	0	0	0	0	0	1
**hCa14**	0	0	0	1	1	1	0	0	0	0	0	0	1
**sFe1**	0	0	0	1	1	1	0	0	0	0	0	0	1
**hFe31**	0	0	0	1	1	1	0	0	0	0	0	0	1
**sCa4**	1	0	0	1	1	1	0	0	0	0	0	0	1

**Study isolate ID**	*mphC*	*msrA*	*norA*	*qacA*	*qacH*	*sdrM*	*sepA*	*tet(K)*	*vatB*	*vgaA*	*vgaALC*	*vgaB*
**sHo4**	0	0	1	0	0	1	1	0	0	0	0	0
**hHo81**	1	1	1	1	0	1	1	0	0	0	0	0
**hHo105**	1	1	1	1	0	1	1	0	0	0	0	0
**sHo15**	0	0	1	1	0	1	1	0	0	0	0	0
**hHo151**	1	1	1	1	0	1	1	0	0	0	0	0
**hHo158**	1	1	1	1	1	1	1	1	0	1	0	0
**hHo183**	1	1	1	1	0	1	1	0	0	0	0	0
**hHo202**	1	1	1	1	0	1	1	0	0	0	0	0
**hHo210**	0	0	1	1	0	1	1	1	0	1	0	0
**hHo73**	0	0	1	0	0	1	1	0	0	0	0	0
**hHo7**	0	0	1	1	0	1	1	1	0	0	0	0
**sHo3**	0	0	1	0	0	1	1	0	0	0	0	0
**sHo8**	1	1	1	0	0	1	1	0	0	0	0	0
**hHo74**	1	1	1	1	0	1	1	0	0	0	0	0
**sHo9**	1	1	1	0	0	1	1	1	0	0	0	0
**hHo93**	0	0	1	0	0	1	1	0	0	0	0	0
**hHo97**	1	1	1	1	0	1	1	1	0	1	0	0
**hHo108**	1	1	1	0	0	1	1	1	0	1	0	0
**hHo143**	1	1	1	1	0	1	1	1	0	0	0	0
**hHo144**	0	0	1	0	0	1	1	0	0	0	0	0
**sHo18**	0	0	1	0	0	1	1	0	0	0	0	0
**hHo173**	0	0	1	0	0	1	1	0	0	0	0	0
**sHo19**	0	0	1	0	0	1	1	0	0	0	0	0
**hHo224**	1	1	1	0	0	1	1	1	0	0	0	0
**hHo234**	0	0	1	0	0	1	1	0	0	0	0	0
hCa3	0	1	1	0	0	1	1	0	0	0	0	0
hCa4	0	0	1	0	0	1	1	1	0	0	0	0
**hFe1**	0	0	1	0	0	1	1	1	0	0	0	0
**hFe4**	1	1	1	1	0	1	1	0	0	0	0	0
hCa16	0	0	1	1	0	1	1	0	1	0	1	1
hCa8	0	1	1	0	0	1	1	0	0	0	0	0

**hFe6**	0	0	1	1	0	1	1	1	0	0	0	0
**hFe9**	0	0	1	1	0	1	1	1	0	0	0	0
**hFe10**	0	0	1	0	1	1	1	0	0	0	0	0
**hFe11**	0	1	1	0	0	1	1	0	0	1	0	0
**hFe13**	0	1	1	0	0	1	1	0	0	1	0	0
**hFe14**	0	0	1	0	0	1	1	0	0	0	0	0
**hFe15**	0	0	1	0	0	1	1	0	0	1	0	0
hCa9	0	0	1	0	0	1	1	0	0	0	0	0
hCa10	0	0	1	0	0	1	1	1	0	0	0	0
sCa1	1	1	1	0	1	1	1	0	0	0	0	0
sCa2	0	0	1	1	1	1	1	1	0	1	0	0
sCa3	0	0	1	0	1	1	1	0	0	0	0	0
hCa11	0	0	1	0	0	1	1	0	0	0	0	0
**hFe17**	0	0	1	1	0	1	1	0	1	0	1	1
**hFe21**	1	1	1	0	0	1	1	0	0	1	0	0
**hFe25**	0	0	1	0	0	1	1	0	0	0	0	0
**hFe28**	0	0	1	0	0	1	1	0	0	0	0	0
**sFe2**	0	0	1	0	0	1	1	0	0	0	0	0
hCa12	1	1	1	1	0	1	1	0	0	0	0	0
hCa13	1	1	1	1	0	1	1	0	0	0	0	0
hCa14	1	1	1	1	0	1	1	0	0	0	0	0
**sFe1**	0	0	1	0	0	1	1	0	0	0	0	0
**hFe31**	0	0	1	0	0	1	1	0	0	0	0	0
sCa4	0	0	1	1	0	1	1	0	0	0	0	0

Table S7. Genotype–phenotype concordance between WGS-detected antimicrobial resistance determinants and phenotypic antimicrobial susceptibility among 55 *Staphylococcus epidermidis* isolates.

**Antimicrobial**	**Phenotypic method**	**WGS determinant(s)**	**P+/G+**	**P–/G–**	**P+/G–**	**P–/G+**	**Agreement (%)**	**Cohen's κ**	**McNemar p-value**	**Predominant discordance**
Oxacillin	MIC	*mecA*	14	12	26	3	47.27	0.101	<0.001	Phenotype R / genotype –
Oxacillin	Disk diffusion	*mecA*	11	13	25	6	43.64	-0.008	<0.001	Phenotype R / genotype –
Erythromycin	Disk diffusion	*ermA, ermC, msrA, mphC*	27	9	10	9	65.45	0.226	1.000	No directional predominance
Erythromycin	MIC	*ermA, ermC, msrA, mphC*	35	4	15	1	70.91	0.221	<0.001	Phenotype R / genotype –

Clindamycin	Disk diffusion	*ermA*, *ermC*, *lnuA*, *vgaA*, *vgaALC*, *vgaB*	14	13	20	8	49.09	0.028	0.036	Phenotype R / genotype −
Clindamycin	MIC	*ermA*, *ermC*, *lnuA*, *vgaA*, *vgaALC*, *vgaB*	14	8	25	8	40.00	−0.107	0.005	Phenotype R / genotype −
Tetracycline	Disk diffusion	*tet(K)*	10	14	27	4	43.64	0.036	<0.001	Phenotype R / genotype −
Tetracycline	MIC	*tet(K)*	12	10	31	2	40.00	0.060	<0.001	Phenotype R / genotype −
Mupirocin	Disk diffusion	*mupA*	2	48	0	5	90.91	0.411	0.063	Genotype + / phenotype S
Gentamicin	Disk diffusion	*aac(6′)-Ie-aph(2″)-Ia*	3	44	0	8	85.45	0.375	0.008	Genotype + / phenotype S

Note: P+/G+ = phenotypically resistant and genotype positive; P−/G− = phenotypically susceptible and genotype negative; P+/G− = phenotypically resistant and genotype negative; P−/G+ = phenotypically susceptible and genotype positive. Disk-diffusion and MIC classifications were analysed independently.

Table S8. Gene-specific concordance between PCR- and WGS-based detection of antimicrobial resistance determinants among 55 *Staphylococcus epidermidis* isolates.

Gene	PCR positive n/55	WGS positive n/55	PCR+/WGS+	PCR+/WGS−	PCR−/WGS+	PCR−/WGS−	Overall agreement (%)	Cohen's κ	McNemar p-value
blaZ	39	36	32	7	4	12	80.0	0.541	0.549
mecA	13	17	13	0	4	38	92.7	0.818	0.125
aac(6′)-Ie-aph(2″)-Ia	11	11	11	0	0	44	100.00	1.000	1.000
tetK	13	14	5	8	9	33	69.1	0.166	1.000
ermA	2	3	0	2	3	50	90.9	−0.046	1.000
ermC	5	11	5	0	6	44	89.1	0.571	0.031
fusB	5	5	1	4	4	46	85.5	0.120	1.000
mupA	3	7	1	2	6	46	85.5	0.134	0.289

Note: PCR+/WGS+ = positive by both methods; PCR+/WGS− = PCR-positive/WGS-negative; PCR−/WGS+ = PCR-negative/WGS-positive; PCR−/WGS− = negative by both methods.

Table S9. Recurrent phenotypic antimicrobial resistance profiles identified among 320 *Staphylococcus epidermidis* isolates.

**Resistance profile**	**N**	**Isolates**
OX–DA–E–TET	30	**hHo12, hHo75, hHo96, hHo99, hHo118, hHo119, hHo147, hHo154, hHo167, hHo173, hHo183, hHo195, hHo197, hHo198, hHo199, hHo201, hHo206, hHo208, hHo210, hHo221, hHo223, hHo224, hHo225, hHo228, hHo240, hHo242, hFe23, hFe25, sFe2, hCa12**
OX–E–TET	22	**sHo21, hHo2, hHo13, hHo48, hHo77, hHo129, hHo137, hHo172, hHo174, hHo176, hHo193, hHo205, hHo207, hHo220, hHo233, hHo234, hHo238, hHo239, hFe2, hFe7, hFe11, sCa4**
OX–E	18	**sHo5, sHo7, sHo8, hHo16, hHo40, hHo41, hHo46, hHo47, hHo57, hHo59, hHo61, hHo63, hHo109, hHo170, hHo178, hHo179, hCa7, hCa17**
OX	17	**sHo1, sHo14, hHo5, hHo15, hHo44, hHo49, hHo52, hHo53, hHo58, hHo60, hHo62, hHo64, hHo65, hHo66, hHo69, hHo70, hHo73**
OX–DA–E	17	**hHo34, hHo54, hHo55, hHo67, hHo117, hHo122, hHo141, hHo142, hHo152, hHo185, hHo186, hHo188, hHo190, hHo241, hFe27, hFe29, hCa16**
DA–E–TET	14	**sHo16, hHo92, hHo102, hHo103, hHo108, hHo113, hHo139, hHo230, hHo243, hFe1, hFe3, hFe22, hFe32, hCa13**
OX–DA–TET	14	**sHo17, hHo100, hHo105, hHo126, hHo163, hHo164, hHo165, hHo209, hHo211, hHo216, hHo217, hFe5, hCa9, hCa11**
P–OX–DA–E–TET	13	**hHo114, hHo171, hHo180, hHo191, hHo192, hHo212, hHo214, hHo222, hHo226, hHo231, hFe4, hFe24, hCa5**
AMP–P–OX–DA–E–TET	9	**hHo86, hHo87, hHo135, hHo143, hHo145, hHo181, hHo213, hHo215, hFe31**
AMP–P–DA–E–TET	7	**hHo124, hHo125, hHo132, hHo136, hHo138, hHo202, hFe21**
E–TET	7	**hHo76, hHo79, hHo89, hHo232, hHo236, hFe9, hFe28**
OX–TET	7	**hHo160, hHo161, hHo162, hHo218, hFe13, sFe1, hCa4**
DA	6	**sHo12, sHo13, sHo19, hHo110, hHo146, hCa10**
DA–TET	5	**hHo128, hHo149, hHo229, hCa3, hCa18**
E	5	**hHo28, hHo38, hHo130, hFe16, hCa15**
P–OX–E	5	**hHo36, hHo37, hHo43, hHo74, hHo184**

AMP–AMC–P–E–TET	4	**hHo81, hHo93, hHo121, hHo131**
AMP–P–E	4	**sHo3, hHo26, hHo80, hFe19**
DA–E	4	**sHo11, hHo140, hHo144, hFe18**
OX–DA	4	**sHo10, hHo78, hHo107, hCa14**
AMP–AMC–P	3	**hHo3, hHo25, hHo106**
AMP–AMC–P–OX–DA–E–TET	3	**sHo15, hHo82, hHo88**
AMP–P–OX–DA–E	3	**hHo133, hHo168, hHo189**
AMP–P–OX–E–TET	3	**hHo94, hHo95, hHo237**
AMP–P–OX–MUP–DA–E	3	**hHo156, hHo158, hHo203**
P–DA–E–TET	3	**hHo153, hHo194, hHo227**
P–OX	3	**hHo8, hHo42, hHo68**
P–OX–DA–E	3	**hHo177, hHo187, hHo196**
TET	3	**hHo235, hCa1, hCa2**
AMP–AMC–P–DA–TET	2	**hHo111, hHo148**
AMP–AMC–P–E	2	**hHo4, hHo91**
AMP–AMC–P–OX	2	**hHo14, hHo18**
AMP–AMC–P–OX–DA–TET	2	**hHo83, hHo97**
AMP–AMC–P–OX–E	2	**hHo6, hHo39**

AMP–P–DA	2	**hHo150**, **hFe30**
AMP–P–OX	2	**sHo2**, **hHo33**
AMP–P–OX–DA–TET	2	**hHo112, hHo134**
AMP–P–OX–TET	2	**hHo20, hHo85**
OX–E–C	2	**hHo30**, **sCa1**
Fully susceptible	12	**sHo6**, **hHo11**, **hHo45, hHo50, hHo51, hHo56, hHo72, hHo98, hHo101, hHo104**, **hFe15**, **hCa8**

Note: The table includes resistance profiles detected in at least two isolates, as well as 12 isolates susceptible to all antimicrobial agents tested. The remaining 49 resistance profiles were unique to individual isolates and are therefore not presented separately. Only antimicrobial agents for which resistance was detected are listed within each recurrent profile.

Figure S2. Heatmap showing MIC–MBC relationships for *Staphylococcus epidermidis* isolates against selected antimicrobial agents. The complete dataset was divided into eight panels (A–H) for clarity, with consecutive subsets of isolates presented in each panel. Columns correspond to the antimicrobial agents oxacillin (OX), vancomycin (VAN), teicoplanin (TEICO), clindamycin (DA), erythromycin (E), and tetracycline (TET). Numerical values represent the $\log_2$(MBC/MIC) ratio for each isolate–antimicrobial combination, with increasing colour intensity indicating a greater separation between MIC and MBC values. Phenotypic susceptibility classification is shown as S (susceptible), I (intermediate), or R (resistant). Blank cells indicate combinations for which either the MIC or MBC value could not be determined.

A. Isolates 1-40

	OX		VAN		TEIKO		DA		E		TET	
sHo1	1	R	1	S	0	S	0	S	2	S	0	S
sHo2	1	R	1	S	9	S	6	S	1	S	3	S
sHo3	3	R	1	R	0	S	5	S		R	9	S
sHo4	1	R	1	S	6	S	6	S	1	S	7	S
sHo5	1	R	1	S	2	S	3	S	6	R	5	S
sHo6	3	R	1	S	9	S	5	S	9	R	5	S
sHo7	3	R	1	S	9	S	5	S	1	R	14	S
sHo8	2	R	2	S	0	S	4	S	1	R	14	S
sHo9	1	R	1	S	2	S	5	R		R		R
sHo10	1	R	1	S	2	S	4	R	2	S	7	S
sHo11	1	R	1	S	2	S	3	R	4	R	5	S
sHo12	2	R	1	S	2	S	2	R	4	S	4	S
sHo13	2	R	1	S	2	S	2	R	4	S	4	S
sHo14	1	R	1	S	1	S	3	S	3	S	5	S
sHo15	1	R	1	S	1	S	4	R	4	R	7	R
sHo16	1	R	1	S	1	S	5	R		R	6	R
sHo17	1	R	1	S	1	S	5	R		R	6	R
sHo18	1	R	1	S	1	S		R		R	6	R
sHo19	1	R	3	S	1	S	5	R	6	R	7	R
sHo20	1	R	1	S	1	S	4	R	3	R	7	R
sHo21	1	R	1	S	1	S	5	S	4	R	3	R
hHo1	1	R	1	S	1	S	4	R	9	R	6	R
hHo2	1	R	1	S	1	S	0	S	1	R	1	S
hHo3	1	R	1	S	1	S	0	S	0	S	1	S
hHo4	1	R	1	S	1	S	4	S	0	S	1	S
hHo5	1	S	1	S	0	S	0	S	0	S	1	S
hHo6	1	R	1	S	1	S	1	S	2	R	1	S
hHo7	1	R	1	S	1	S	1	S	1	R	3	S
hHo8	1	S	1	S	0	S	0	S	2	S	1	S
hHo9	1	S	1	S	0	S	0	S	7	R	1	S
hHo10	1	R	1	S	1	S	1	S	6	R	2	S
hHo11	1	S	1	S	1	S	1	S	7	R	3	R
hHo12	1	R	1	S	1	I	4	R	1	R	2	S
hHo13	1	S	1	S	1	S	2	S	3	S	1	S
hHo14	1	R	1	S	1	S	0	S	1	S	1	S
hHo15	1	S	1	S	0	S	1	S	1	S	2	S
hHo16	1	R	1	S	1	S	1	S	1	R	1	S
hHo17	1	R	1	S	3	S	1	S	1	S	1	S
hHo18	1	S	1	S	1	S	1	S	1	S	1	S
hHo19	1	R	1	S	0	S	6	S	5	S	6	R

B. Isolates 41-80

	OX		VAN		TEIKO		DA		E		TET	
hHo20	2	R	1	S	0	S	6	S	5	S	7	R
hHo21	1	R	6	S	2	S	5	S	6	R	7	R
hHo22	1	R	1	S	8	S	7	R	5	R	8	S
hHo23	1	R	1	S	0	S	5	R	5	R	9	S
hHo24	1	R	2	S	8	S	1	S	2	S	6	S
hHo25	1	R	1	S	1	S	7	S	1	S	8	S
hHo26	1	R	1	S	1	S	5	S	5	R	11	S
hHo27	1	R	1	S	0	S	5	S	3	S	7	S
hHo28	1	R	1	S	1	S	6	S	4	R	7	S
hHo29	1	R	1	S	1	S	6	S	4	R	7	S
hHo30	1	R	1	S	0	S	6	S	4	R	9	S
hHo31	1	R	3	S	1	S	6	R	4	R	8	R
hHo32	1	R	1	S	0	S	6	R	7	R	8	S
hHo33	1	R	1	S	1	I	1	S	4	S	9	R
hHo34	1	R	6	S	1	S	8	R	7	R	7	R
hHo35	3	R	1	S	0	S	5	S	5	S	9	S
hHo36	1	R	1	S	9	S	6	S	5	R	9	S
hHo37	1	R	1	S	5	S	5	S	9	R	9	S
hHo38	1	R	1	R	0	S	5	S	5	R	3	S
hHo39	1	R	1	S	0	S	5	S	4	R	8	R
hHo40	1	R	1	S	0	S	6	S	7	R	9	R
hHo41	1	R	1	S	7	S	5	S	6	R	11	S
hHo42	3	R	1	R	2	S		S	5	S	8	S
hHo43	3	R	1	S	2	S	5	S	7	R	7	R
hHo44	1	R	1	S	8	S	5	S	7	S	1	S
hHo45	1	R	1	S	1	S	7	S	6	S	11	S
hHo46	1	R	1	S	6	S	5	S	5	R	3	S
hHo47	4	R	1	S	2	S	6	S	7	R	8	S
hHo48	2	R	1	S	4	S	5	S	3	R	5	R
hHo49	3	R	2	S	5	S	6	S	4	S	3	S
hHo50	1	R	1	S	2	S	6	S	4	S	6	S
hHo51	1	R	6	S	6	S	5	S	5	S	7	S
hHo52	1	R	1	S	1	S	5	S	5	S	6	S
hHo53	1	R	7	S	6	S	5	S	7	S	13	S
hHo54	1	R	2	S	0	S	8	R	6	R	7	S
hHo55	1	R	1	S	1	S	7	R	6	R	7	S
hHo56	1	R	8	S	0	S	5	S	1	S	12	S
hHo57	1	R	1	S	1	S	8	R		R	13	S
hHo58	1	R	3	S	0	S	1	S	0	S	2	S
hHo59	1	R	1	S	1	S	6	R	8	R	11	S

C. Isolates 81–120

	OX		VAN		TEIKO		DA		E		TET	
hHo60	1	R	2	S	0	S	1	S	4	S	11	S
hHo61	2	R	1	S	6	S	3	S		R	12	S
hHo62	1	R	7	S	6	S	3	S		S	11	S
hHo63	1	R	2	S	0	S	4	S	6	R	10	S
hHo64	1	R	1	S	0	S	2	S	3	S	2	S
hHo65	1	R	1	S	0	S	6	S	1	S	7	S
hHo66	1	R	1	S	8	S	5	S	5	S	8	S
hHo67	1	R	1	S	2	S	5	S	6	R	13	S
hHo68	1	R	6	S	0	S	6	S	1	S	6	S
hHo69	1	R	5	S	0	S	3	S	1	S	14	S
hHo70	1	R	2	S	0	S	5	S	1	S	8	S
hHo71	1	R	3	S	0	S	5	R		R	10	S
hHo72	3	R	1	S	0	S	4	S	4	S	10	S
hHo73	1	R	1	S	0	S	5	S	3	S	8	S
hHo74	1	R	1	S	0	S	7	S	6	R	14	S
hHo75	1	R	5	S	2	S	6	R	4	R		R
hHo76	1	R	1	S	2	S	5	R	2	R	8	R
hHo77	1	R	3	S	2	S	10	R	4	R	7	S
hHo78	1	R	1	S	1	S	4	R	2	S	8	S
hHo79	3	R	4	S	3	S	6	R	4	R	9	R
hHo80	2	R	2	S	3	S	7	R		R	6	S
hHo81	2	R	1	S	4	S	4	R		R	6	R
hHo82	2	R	4	S	5	S	6	R		R	8	R
hHo83	3	R	4	S	2	S	7	R	4	R	7	R
hHo84	1	R	2	S	2	S	5	R		R	9	R
hHo85	1	R	1	S	6	S	5	R		R	6	R
hHo86	1	R	5	S	2	S	7	R	7	S		R
hHo87	1	R	1	S	2	S	5	R	6	S	3	R
hHo88	1	R	4	S	1	S	4	R	7	R	5	R
hHo89	1	R	3	S	2	S	8	R	6	R	9	R
hho90	1	R	7	S	2	S	5	R		R	8	R
hHo91	1	R	3	S	2	S	12	R		R	6	S
hHo92	1	R	2	S	2	S	7	R	5	R	7	R
hHo93	1	R	4	S	2	S	6	R	3	R	7	R
hHo94	1	R	1	S	2	S	8	R		R	5	R
hHo95	2	R	2	S	2	S	6	R	5	R	4	R
hHo96	3	R	1	S	2	S	4	R	5	R	6	R
hHo97	1	R	2	S	1	S	1	R	4	R	3	R
hHo98	2	R	1	S	1	S	7	S	6	S	5	S
hHo99	1	R	1	S	1	S	4	R	4	R	4	R

D. Isolates 121–160

	OX		VAN		TEIKO		DA		E		TET	
hHo100	2	R	1	S	2	S	5	R	3	R	6	R
hHo101	2	R	1	S	1	S	4	S	5	S	7	S
hHo102	2	R	1	S	1	S		R		R	2	R
hHo103	1	R	1	S	1	S	2	R	3	R	4	R
hHo104	1	R	2	S	1	S	6	S	7	S	6	S
hHo105	1	R	1	S	1	S	4	R	3	R	5	R
hHo106	2	R	1	S	1	S	4	S	4	S	7	S
hHo107	1	S	1	S	1	S	4	S	4	S	4	S
hHo108	1	R	1	S	1	S	4	R	4	R	4	R
hHo109	1	R	1	S	1	S	6	S	5	R	7	S
hHo110	1	R	1	S	3	S	5	R	6	S	8	S
hHo111	1	R	1	S	1	S	5	R	4	R	4	R
hHo112	1	R	1	S	1	S	5	R	9	R	5	R
hHo113	1	R	1	R	1	S	4	R	3	R	4	R
hHo114	1	R	1	S	1	S	3	R	4	R	5	R
hHo115	1	R	1	S	1	S	3	R	4	R	7	S
hHo116	1	R	1	S	1	S	3	R	4	R	7	S
hHo117	1	R	1	S	1	S	6	R	4	R	7	S
hHo118	1	R	1	S	1	S	5	R	1	R	5	R
hHo119	1	R	1	S	1	S	6	R	6	R	7	R
hHo120	1	R	1	S	1	S	6	R	5	R	6	R
hHo121	1	R	1	S	1	S	6	R		R	6	R
hHo122	1	R	1	S	1	S	5	R	4	R	6	R
hHo123	1	R	1	S	1	S	6	R	4	R	2	R
hHo124	1	R	1	S	1	S	6	R	9	R	5	R
hHo125	1	R	1	S	1	S	6	R		R		R
hHo126	1	R	1	S	1	S	7	R		R	5	R
hHo127	1	R	1	S	1	S	5	R		R	6	R
hHo128	1	R	1	S	1	S	5	R	6	R	6	R
hHo129	1	R	1	S	1	S	4	R	6	R	8	R
hHo130	1	R	1	S	1	S	6	R		R	5	R
hHo131	1	R	1	S	1	S	6	R	6	R	5	R
hHo132	1	R	1	S	1	S		R	5	R	8	R
hHo133	1	R	1	S	1	S		R	5	R	5	R
hHo134	1	R	1	S	1	S		R		R	7	R
hHo135	1	R	1	S	1	S	5	R	5	R	5	R
hHo136	1	R	1	S	1	S	4	R	6	R	9	R
hHo137	1	R	1	S	1	S	5	R	10	R	3	R
hHo138	1	R	1	S	1	S	5	R	9	R	5	R
hHo139	1	R	1	S	1	S	4	R	6	R	5	R

E. Isolates 161–200

	OX		VAN		TEIKO		DA		E		TET	
hHo140	1	R	1	S	1	S	6	R		R	5	R
hHo141	1	R	1	S	1	S	5	R	9	R	5	R
hHo142	1	R	1	S	1	S		R		R	6	R
hHo143	1	R	1	S	1	S	6	R		R		R
hHo144	1	R	1	S	1	S	4	R	6	R	3	S
hHo145	1	R	1	S	0	S		R	5	R	5	R
hHo146	1	R	1	S	1	S	5	R	2	S	7	S
hHo147	1	R	1	S	1	S	5	R		R	5	R
hHo148	1	R	1	S	1	S	5	R		R	6	R
hHo149	1	R	1	S	1	S	5	R		R	6	R
hHo150	1	R	1	S	3	S	7	R	9	R	4	R
hHo151	1	R	1	S	1	S	4	R		R	4	R
hHo152	1	R	1	S	1	S	4	R		R	4	R
hHo153	1	R	1	S	1	S	3	R	5	R	4	R
hHo154	1	R	2	S	2	S	2	R	5	R	4	R
hHo155	1	R	1	S	1	S	1	R	2	R	4	R
hHo156	1	R	1	S	1	S	1	R	1	R		R
hHo157	2	R	1	S	4	S		R		R	1	R
hHo158	1	R	1	S	1	S	1	R	1	R	1	R
hHo159	1	R	1	S	1	S	1	R	1	R	1	R
hHo160	1	R	1	S	2	S	1	R	4	R	4	R
hHo161	1	R	1	S	2	S	1	R	4	R	4	R
hHo162	1	R	4	S	1	S	4	R	3	R	5	R
hHo163	1	R	2	S	1	S	4	R	5	R	5	R
hHo164	1	R	1	S	1	S		R		R	1	R
hHo165	1	R	1	S	1	S		R	8	S	4	R
hHo166	1	R	1	S	3	S		R		R	2	R
hHo167	1	R	1	S	3	S	2	R	3	R	8	R
hHo168	1	R	1	S	1	S	1	R	2	R	4	R
hHo169	1	R	1	S	1	S	1	R	3	R	2	R
hHo170	1	R	1	S	3	S	1	R	2	R	5	R
hHo171	1	R	1	S	2	S	1	R	3	R	4	R
hHo172	1	R	1	S	1	S	1	R	2	R	4	R
hHo173	1	R	1	S	1	S		R		R	4	R
hHo174	1	R	1	S	1	S	4	R	1	R	3	R
hHo175	1	R	1	S	1	S		R		R		R
hHo176	1	R	1	S	1	S	1	R	3	R	3	R
hHo177	1	R	1	S	1	S	5	R	5	R	8	S
hHo178	1	R	1	S	3	S	3	R	4	S	6	S
hHo179	1	R	1	S	1	S	1	R	6	S	2	R

F. Isolates 201–240

	OX		VAN		TEIKO		DA		E		TET	
hHo180	1	R	1	S	3	S	1	R	9	S	2	R
hHo181	1	R	1	S	2	S	1	R	4	S	4	R
hHo182	1	R	1	S	1	S	1	R	2	R	4	R
hHo183	1	R	1	S	1	S	5	R	4	S	4	R
hHo184	1	R	1	S	5	S	2	R	1	R	4	R
hHo185	1	R	1	S	3	S		R	2	R	3	R
hHo186	1	R	1	S	4	S	1	R	2	R	5	R
hHo187	1	R	1	S	3	S	3	R	4	R	2	R
hHo188	1	R	1	S	1	S	4	R	4	R	5	R
hHo189	1	R	1	S	1	S	1	R	4	R	4	R
hHo190	3	R	2	S	1	S	3	R	5	R	4	R
hHo191	1	R	1	S	1	S	1	R	3	R	2	R
hHo192	1	R	1	S	3	S	1	R	5	R	5	R
hHo193	1	R	1	S	3	S	1	R	5	R	6	S
hHo194	1	R	1	S	2	S	1	R	4	S	4	R
hHo195	1	R	1	S	1	S	1	R	3	R	4	R
hHo196	1	R	1	S	1	S	1	R	5	R	2	R
hHo197	1	R	1	S	3	S	1	R	4	S	5	R
hHo198	1	R	1	S	3	S	1	R	4	S	4	R
hHo199	1	R	1	S	1	S	1	R	4	S	4	R
hHo200	1	R	2	S	3	S	1	R	5	R	5	R
hHo201	1	R	1	S	1	S	4	R	5	R	5	R
hHo202	1	R	1	S	2	S	4	R		R	5	R
hHo203	1	R	1	S	1	S	6	R	3	R	7	S
hHo204	1	R	1	S	2	S	4	R		R		R
hHo205	1	R	1	S	2	S	1	R	4	R	8	S
hho206	1	R	1	S	3	S	1	R	1	R	3	R
hHo207	1	R	1	S	1	S	1	R	4	R	5	S
hHo208	1	R	1	S	1	S	4	R	7	S	7	S
hHo209	1	R	1	S	1	S	4	R	7	S	7	S
hHo210	1	R	2	S	1	S		R		R		R
hHo211	1	R	1	S	1	S	1	R	5	R	3	R
hHo212	1	R	1	S	2	S		R	5	R	8	R
hHo213	1	R	2	S	4	S	1	R	2	R	4	R
hHo214		S	2	S	2	S	4	R	3	R	5	R
hHo215	1	R	2	S	2	S	1	R	3	R	2	R
hHo216	1	R	1	S	1	S	1	R	3	R	6	S
hho217	1	R	1	S	3	S	1	R	5	R	5	R
hHo218	1	R	1	S	2	S	1	R	4	S	4	R
hHo219	1	R	1	S	1	S	1	R	3	R	4	R

G. Isolates 241–280

	OX		VAN		TEIKO		DA		E		TET	
hHo220	1	R	1	S	1	S	1	R	5	R	3	R
hHo221	1	R	1	S	3	S	1	R	4	S	5	R
hHo222	1	R	1	S	2	S	1	R	4	S	4	R
hHo223	1	R	1	S	1	S	1	R	4	S	4	R
hHo224	1	R	1	S	3	S	2	R		R		R
hHo225	1	R	1	S	3	S	4	R	2	R	7	R
hHo226	1	R	1	S	4	S	4	R	3	R	6	R
hHo227	1	R	5	S	7	S	7	R	4	R	8	R
hHo228	1	R	2	S	4	S	4	R	4	R	7	R
hHo229	1	R	2	S	4	S	5	R	6	R	3	R
hHo230	1	R	1	S	2	S	6	R	7	S	4	R
hHo231	1	R	1	S	2	S	4	R	6	S	4	R
hHo232	1	R	3	S	1	S	6	S	2	R	8	S
hHo233	1	R	1	S	1	S	5	S	4	S	5	S
hHo234	1	R	2	S	1	S	7	S	4	S	4	R
hHo235	1	R	3	S	1	S	5	S	4	R	6	R
hHo236	1	R	3	S	1	S	6	S	2	R	3	R
hHo237	1	R	1	S	1	S	5	S	2	R	6	R
hHo238	1	R	2	S	1	S	4	S	2	R	6	R
hHo239	1	R	1	S	2	S	7	S	4	R	2	R
hHo240	1	R	1	S	1	S	7	R	2	R	8	S
hHo241	1	R	1	S	1	S	4	R	2	R	3	R
hHo242	1	R	1	S	1	S	5	R	2	R	2	R
hHo243	1	R	1	S	1	S	7	R	3	R	6	R
hFe1	5	S	2	S	5	S	5	R	3	R	3	R
hFe2	5	S	2	S	5	S	5	R	4	R	2	R
hFe3	5	S	2	S	1	S	5	R	4	R	2	R
hFe4	5	S	2	S	3	S	7	R	5	R	10	R
hFe5	5	S	2	S	1	S	5	R	2	R	6	R
hFe6	3	S	2	S	1	S		R		R		R
hFe7	1	R	2	S	1	S	6	S	2	R	6	R
hFe8	1	R	2	S	1	S	5	S	3	R	3	R
hFe9	1	R	2	S	1	S	6	S	4	R	2	R
hFe10	1	R	2	S	1	S	6	S	4	R	6	R
hFe11	3	S	2	S	5	S	6	S	2	R	3	R
hFe12	1	R	2	S	5	S	5	S	3	R	2	R
hFe13	1	R	2	S	3	S	6	S	2	R	8	S
hFe14	1	R	3	S	3	S	5	S	2	R	3	R
hFe15	1	R	3	S	5	S	4	S	3	R	6	R
hFe16	1	R	3	S	3	S	5	S	4	R	3	R

H. Isolates 281–320

	OX		VAN		TEIKO		DA		E		TET	
hFe17	1	R	3	S	7	S		R		R	6	R
hFe18	1	R	2	S	1	S	5	R	2	R	6	R
hFe19	3	S	2	S	3	S	6	S	4	R	2	R
hFe20	1	R	3	S	3	S	7	S	4	R	2	R
hFe21	1	R	3	S	4	S	5	R	3	R	6	R
hFe22	1	R	2	S	3	S	6	R	4	R	2	R
hFe23	1	R	2	S	3	S	6	R	4	R	2	R
hFe24	6	S	2	S	7	S	6	R	3	R	9	R
hFe25	1	R	2	S	1	S	5	R	2	R	8	S
hFe26	1	R	2	S	3	S	6	S	2	R	6	R
hFe27	1	R	2	S	3	S	5	R	4	S	3	R
hFe28	1	R	2	S	3	S	5	S	4	S	3	R
hFe29	1	R	2	S	1	S	5	R	4	R	2	R
hFe30	1	R	2	S	5	S	7	R	10	R	8	R
hFe31	1	R	2	S	3	S	5	R		R	14	S
hFe32	1	R	2	S	3	S	6	R	10	R	5	S
sFe1	1	R	4	S	1	S	7	R	8	R	8	R
sFe2	1	R	2	S	1	S	7	R	4	R	2	R
hCa1	5	S	3	S	1	S	6	R	2	R	3	R
hCa2	5	S	4	S	1	S	5	R	4	R	6	R
hCa3	5	S	3	S	1	S	5	R	3	R	2	R
hCa4	5	S	2	S	1	S	5	R	3	R	3	R
hCa5	4	S	2	S	3	S	6	R		R	9	S
hCa6	4	S	2	S	3	S	6	R		R	7	R
hCa7	4	S	2	S	1	S	5	R	4	R	2	R
hCa8	4	S	2	S	1	S	5	R	4	R	2	R
hCa9	6	S	2	S	1	S	5	R	2	R	3	R
hCa10	6	S	2	S	1	S	5	R	3	R	6	R
hCa11	6	S	4	S	3	S	5	R	2	R	3	R
hCa12	6	S	2	S	3	S	5	R	4	R	3	R
hCa13	6	S	2	S	5	S	5	R	4	R	3	R
hCa14	6	S	4	S	1	S	6	R	4	R	2	R
hCa15	1	R	2	S	3	S	4	S	2	R	3	R
hCa16	4	S	4	S	1	S	6	R	4	R	2	R
hCa17	4	S	2	S	3	S	5	R	4	R		S
hCa18	4	S	2	S	1	S	5	R	4	R		S
sCa1	1	R	2	S	3	S	7	S	2	R	6	R
sCa2	1	R	2	S	3	S	5	R	2	R	7	S
sCa3	1	R	2	S	5	S	5	R	3	R	5	S
sCa4	4	S	2	S	3	S	5	S	3	R	2	R

Table S10. Genotype–phenotype concordance between PCR-detected antimicrobial resistance genes and phenotypic antimicrobial susceptibility among *Staphylococcus epidermidis* isolates.

Comparison	Method	n	TP	FP	FN	TN	Agreement (%)	Sensitivity (%)	Specificity (%)	Cohen's κ	McNemar p-value
***mecA/mecC*–OX**	MIC	320	44	9	240	27	22.2	15.5	75.0	−0.025	<0.001
***vanA/B*–VAN**	MIC	320	0	3	4	313	97.8	0.0	99.1	−0.011	1.000
***vanA/B*–TEICO**	MIC	320	0	3	0	317	99.1	—	99.1	0.000	0.250
***ermA/B/C*–DA**	MIC	320	25	8	192	95	37.5	11.5	92.2	0.026	<0.001
***ermA/B/C*–E**	MIC	320	28	5	217	70	30.6	11.4	93.3	0.024	<0.001
***tetK/L/M/O*–TET**	MIC	320	56	24	152	88	45.0	26.9	78.6	0.043	<0.001
***blaZ*–AMP**	Disk diffusion	320	59	103	25	133	60.0	70.2	56.4	0.205	<0.001
***blaZ*–P**	Disk diffusion	320	84	78	43	115	62.2	66.1	59.6	0.246	0.002
***mecA/mecC*–OX**	Disk diffusion	320	36	17	179	88	38.8	16.7	83.8	0.004	<0.001
***mupA*–MUP**	Disk diffusion	320	2	3	12	303	95.3	14.3	99.0	0.192	0.035
***fusB*–FD**	Disk diffusion	320	0	26	5	289	90.3	0.0	91.7	−0.027	<0.001
***aac(6′)-Ie-aph(2′′)-Ia*–CN**	Disk diffusion	320	4	9	2	305	96.6	66.7	97.1	0.406	0.065
***aac(6′)-Ie-aph(2′′)-Ia*–TOB**	Disk diffusion	320	0	13	0	307	95.9	—	95.9	0.000	<0.001
***ermA/B/C*–DA**	Disk diffusion	320	19	14	153	134	47.8	11.0	90.5	0.015	<0.001
***ermA/B/C*–E**	Disk diffusion	320	23	10	190	97	37.5	10.8	90.7	0.010	<0.001
***tetK/L/M/O*–TET**	Disk diffusion	320	45	35	133	107	47.5	25.3	75.4	0.006	<0.001

Note: TP = genotype-positive/phenotype-resistant; FP = genotype-positive/phenotype-susceptible; FN = genotype-negative/phenotype-resistant; TN = genotype-negative/phenotype-susceptible. For binary genotype–phenotype concordance analyses, MIC results classified as intermediate (I) were grouped with susceptible/non-resistant results. Disk-diffusion results recorded as R/S were treated as resistant.

Table S11. Specific growth rates and doubling times of representative *Staphylococcus epidermidis* isolates and reference strains at 37 °C, 38 °C, and 39 °C.

**Isolate / reference strain**	**μ 37 °C (h^{-1})**	**tD 37 °C (h)**	**μ 38 °C (h^{-1})**	**tD 38 °C (h)**	**μ 39 °C (h^{-1})**	**tD 39 °C (h)**	**Temperature-dependent growth pattern**
**hHo236**	0.1646	4.21	0.1425	4.86	0.1633	4.25	Decrease followed by recovery
**hHo238**	0.0779	8.89	0.0926	7.48	0.0955	7.26	Gradual increase
**hHo240**	0.1619	4.28	0.1985	3.49	0.1392	4.98	Peak at 38 °C
**hHo242**	0.1666	4.16	0.1920	3.61	0.1418	4.89	Peak at 38 °C
**hHo243**	0.1617	4.29	0.1404	4.94	0.1659	4.18	Decrease followed by recovery
**sHo21**	0.1215	5.71	0.1361	5.09	0.1960	3.54	Gradual increase
**hFe5**	0.2016	3.44	0.1669	4.15	0.1533	4.52	Gradual decrease
**sFe1**	0.1997	3.47	0.1898	3.65	0.1978	3.50	Temperature-independent
**sFe2**	0.2039	3.40	0.1924	3.60	0.2000	3.47	Temperature-independent
**hCa12**	0.1550	4.47	0.1539	4.50	0.1634	4.24	Temperature-independent
**hCa13**	0.1955	3.55	0.1753	3.95	0.1829	3.79	Decrease followed by recovery
**hCa14**	0.1632	4.25	0.1413	4.90	0.1555	4.46	Decrease followed by recovery
**hCa16**	0.1680	4.13	0.1705	4.07	0.1901	3.65	Gradual increase
**sCa1**	0.2158	3.21	0.1223	5.67	0.1339	5.18	Decrease followed by recovery
**sCa4**	0.1621	4.28	0.1815	3.82	0.1700	4.08	Peak at 38 °C
S. aureus ATCC 43300	0.1504	4.61	0.1335	5.19	0.1315	5.27	Gradual decrease
S. epidermidis PCM 1869	0.1006	6.89	0.0936	7.41	0.0940	7.37	Temperature-independent

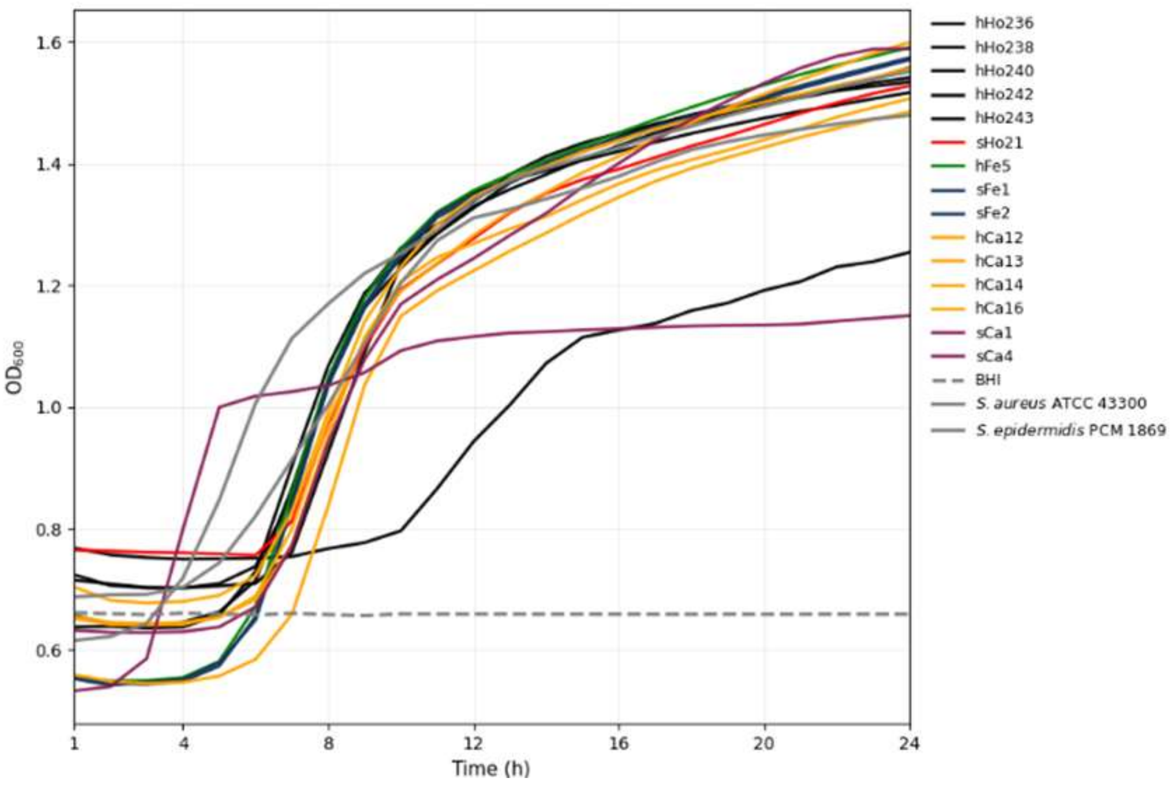

Figure S3a. Growth curves of all selected strains in 37 ℃.

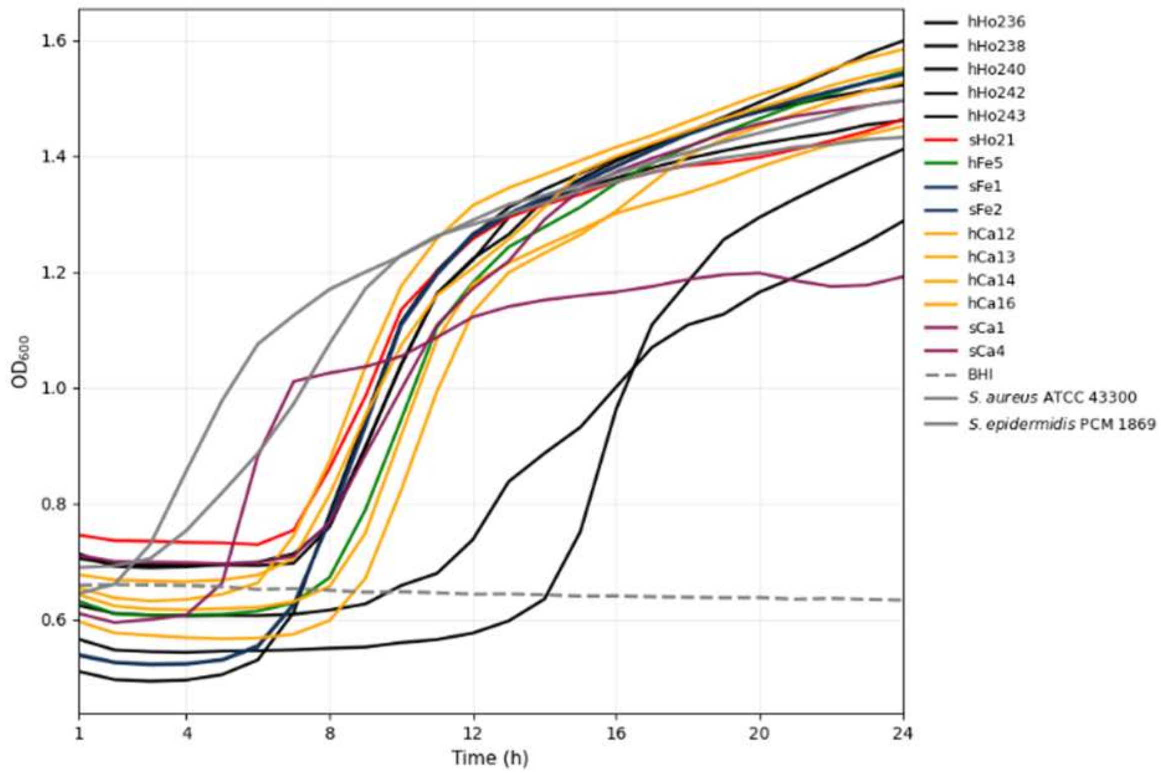

Figure S3b. Growth curves of all selected strains in 38 ℃.

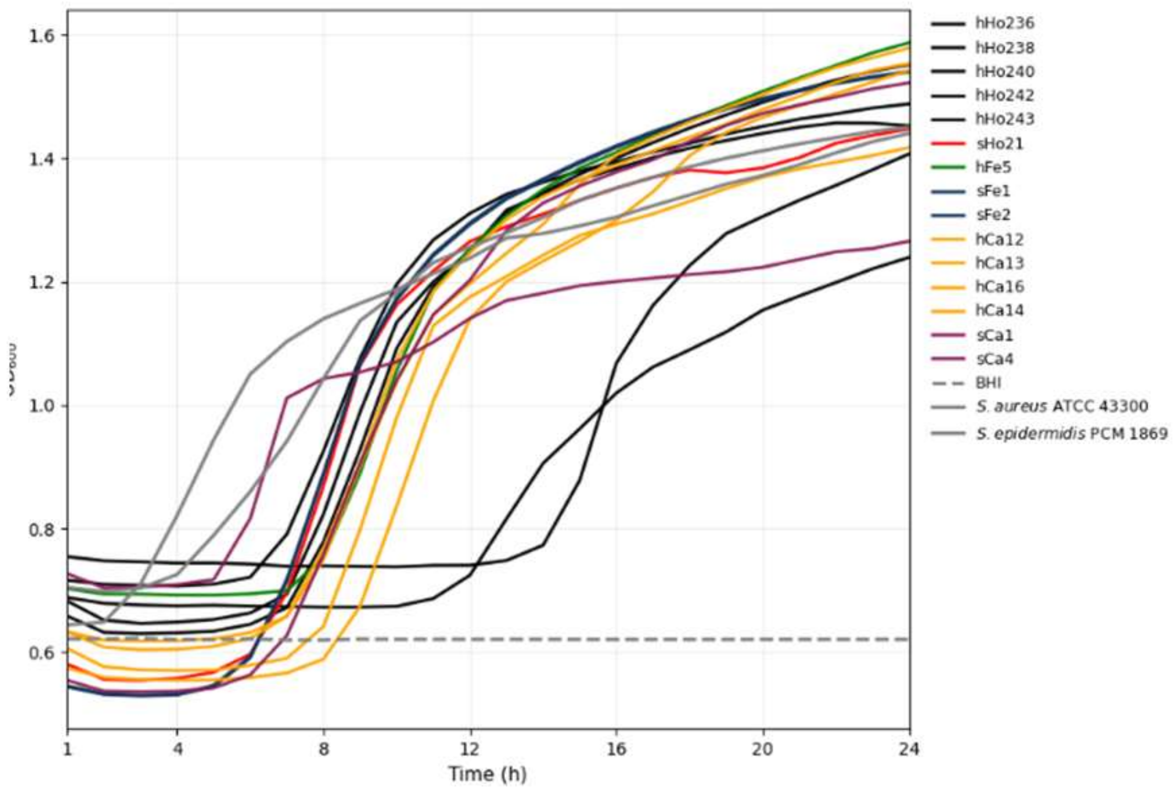

Figure S3c. Growth curves of all selected strains in 39 ℃.

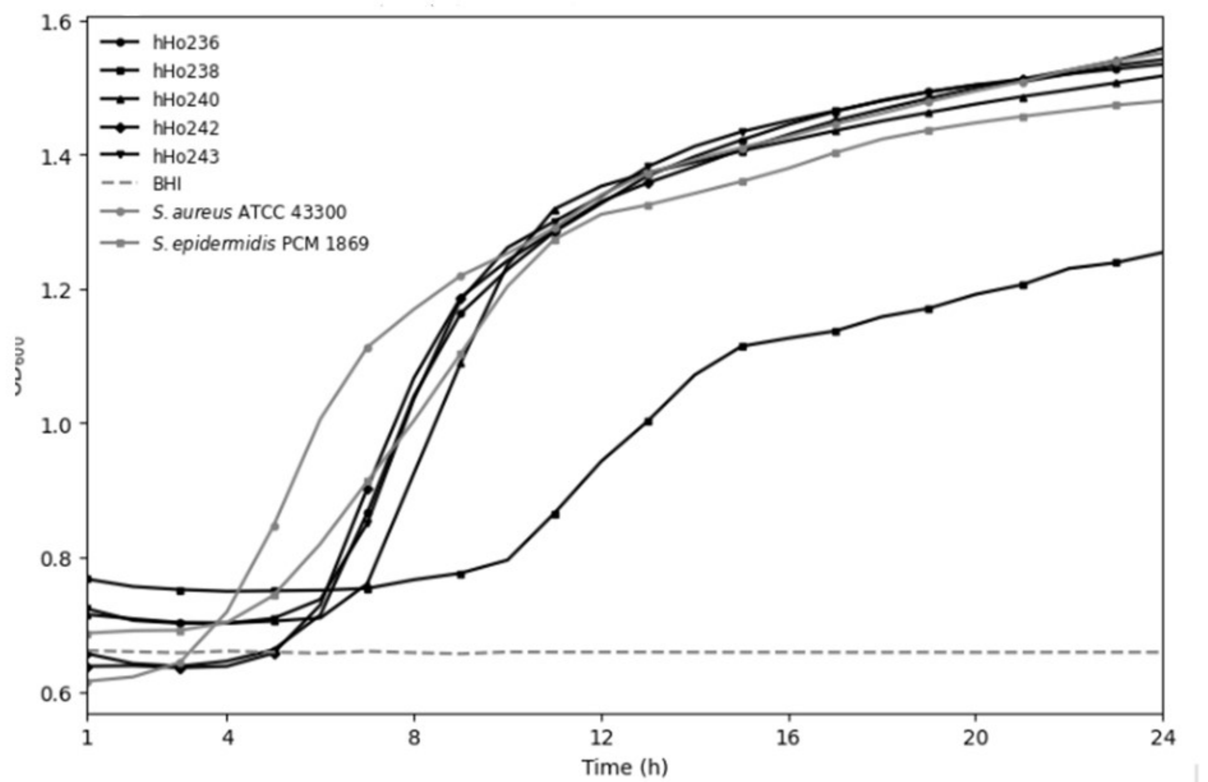

Figure S3d. Growth curves of all selected hHo strains in 37 ℃.

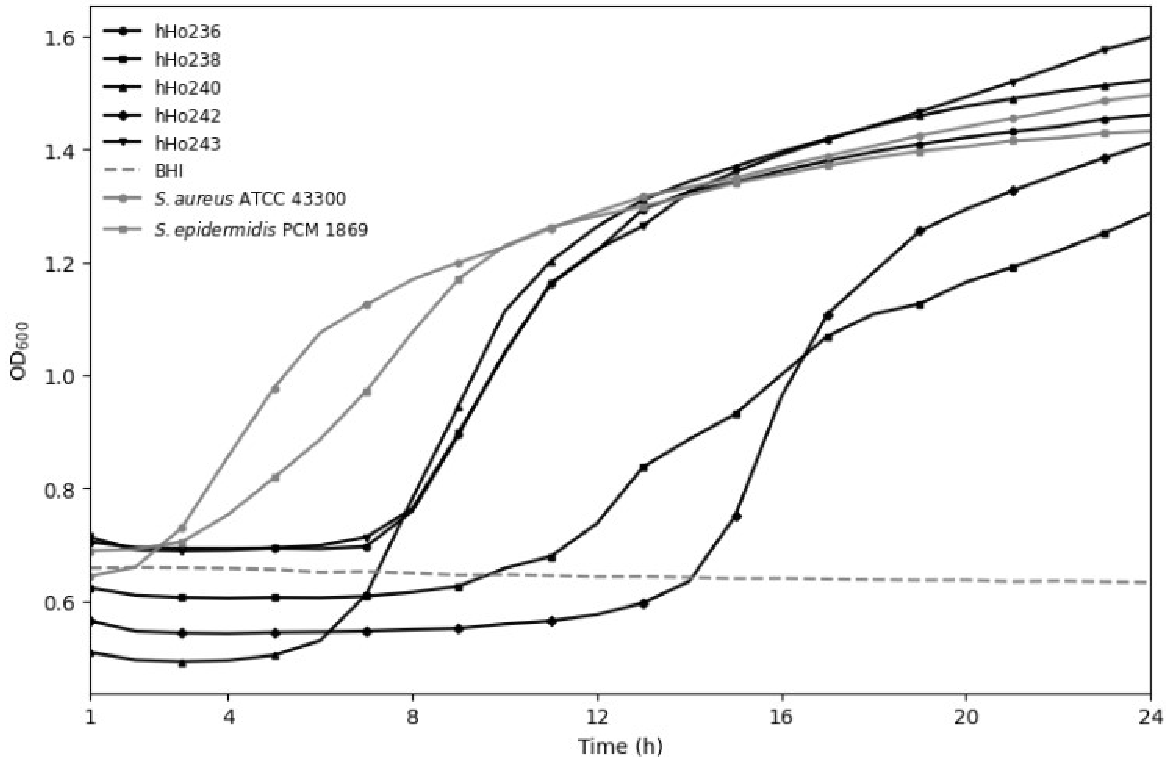

Figure S3e. Growth curves of all selected hHo strains in 38 ℃.

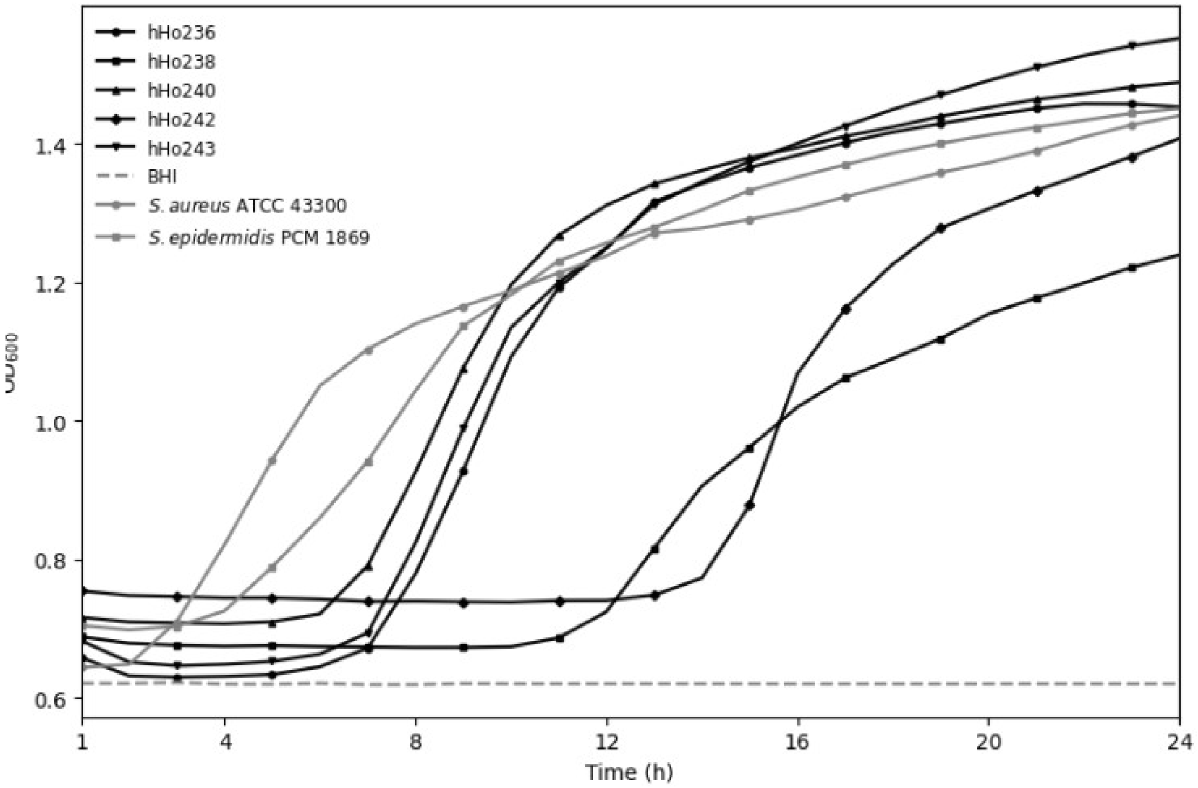

Figure S3f. Growth curves of all selected hHo strains in 39 ℃.

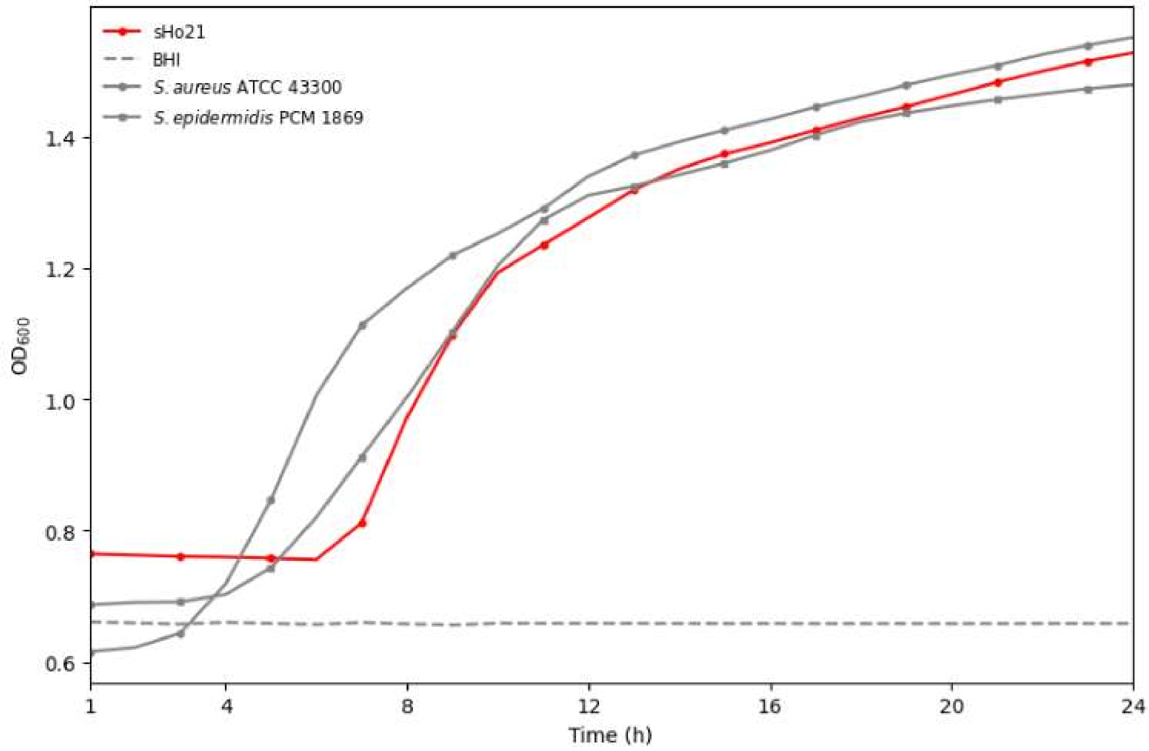

Figure S3g. Growth curves of all selected sHo strains in 37 ℃.

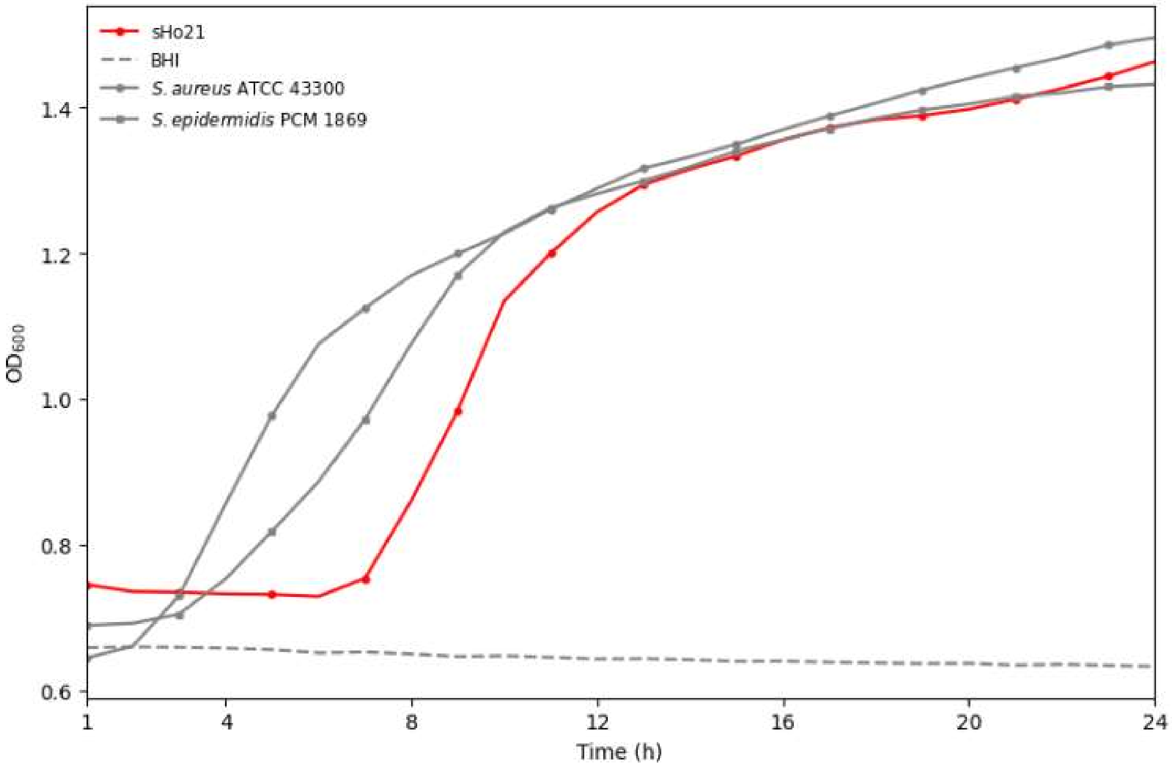

Figure S3h. Growth curves of all selected sHo strains in 38 ℃.

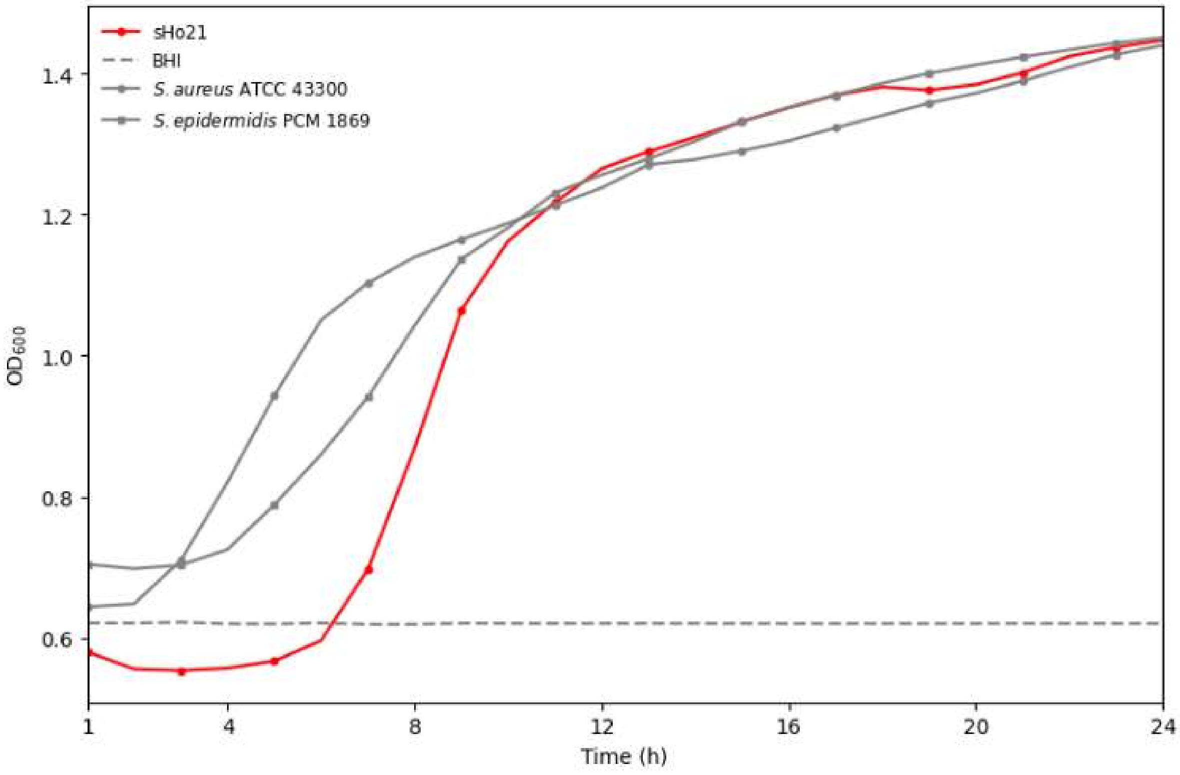

Figure S3i. Growth curves of all selected sHo strains in 39 ℃.

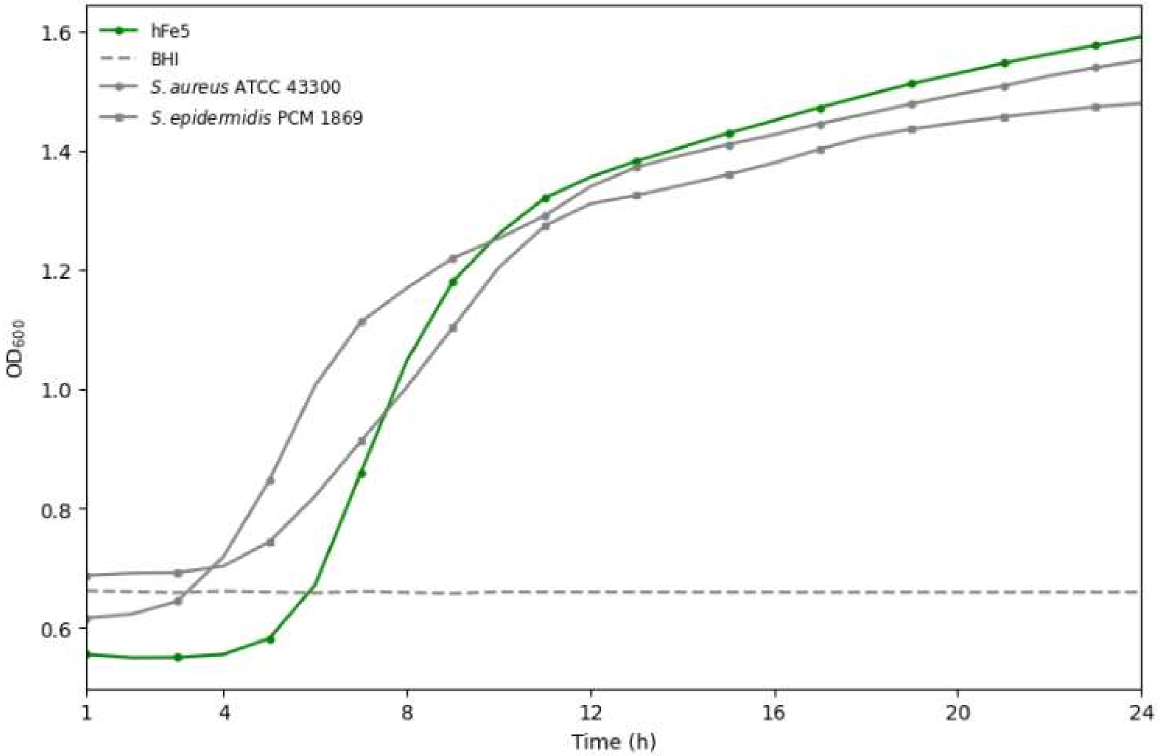

Figure S3j. Growth curves of all selected hFe strains in 37 ℃.

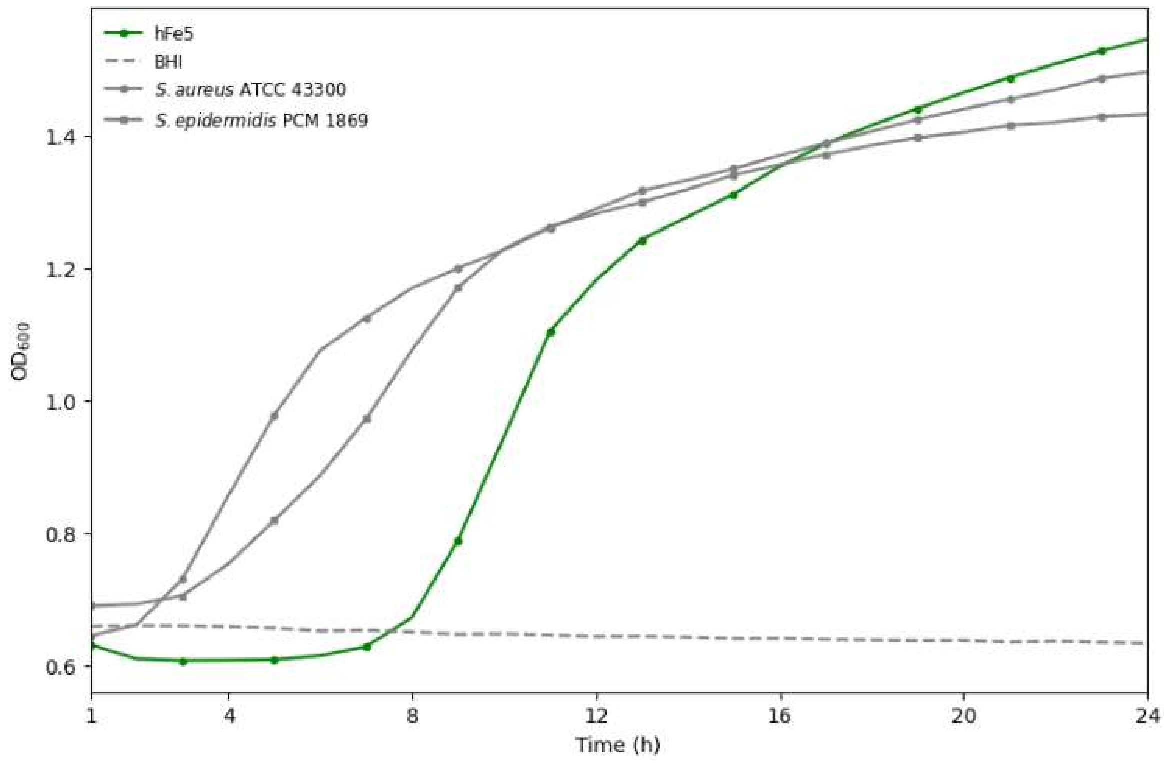

Figure S3k. Growth curves of all selected hFe strains in 38 ℃.

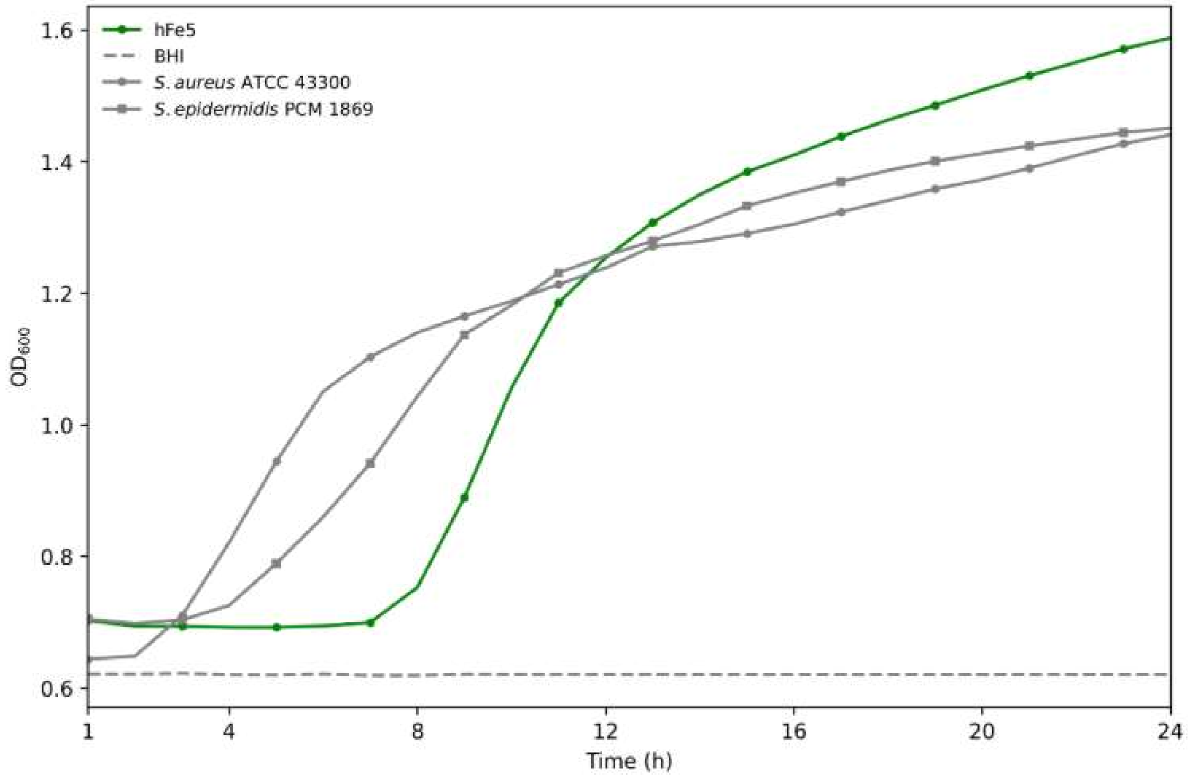

Figure S3l. Growth curves of all selected hFe strains in 39 ℃.

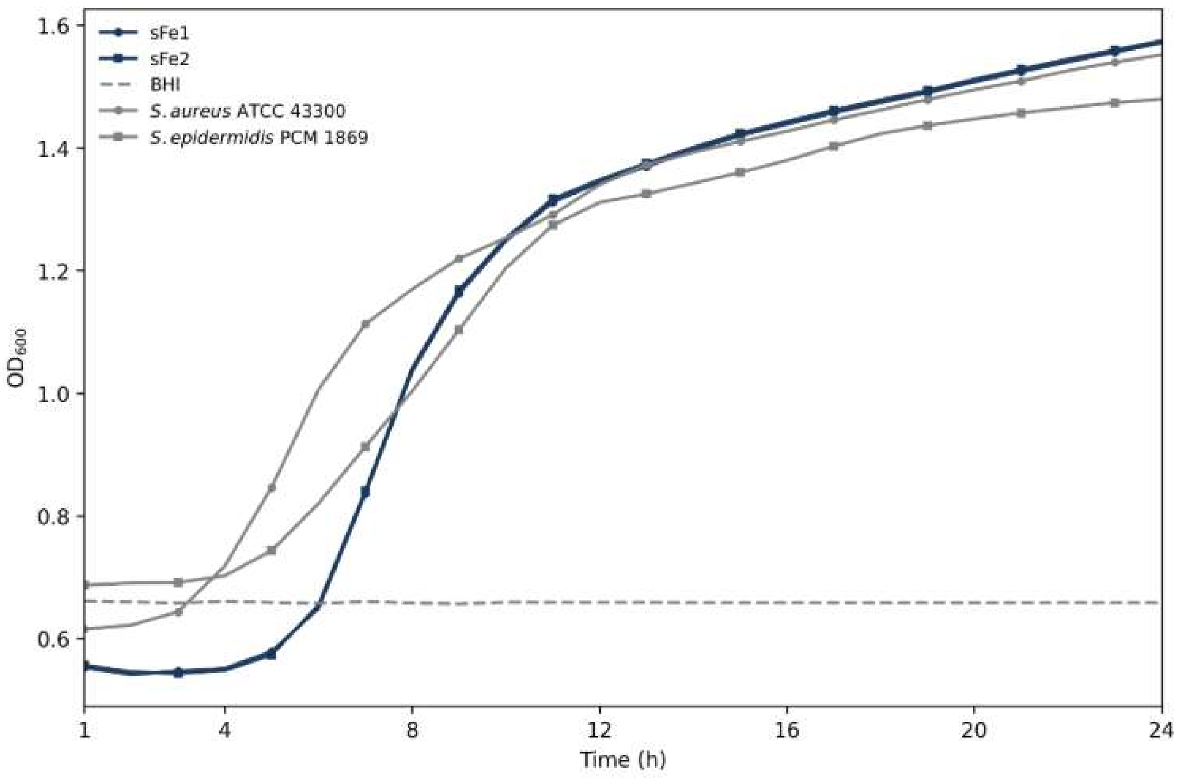

Figure S3m. Growth curves of all selected sFe strains in 37 ℃.

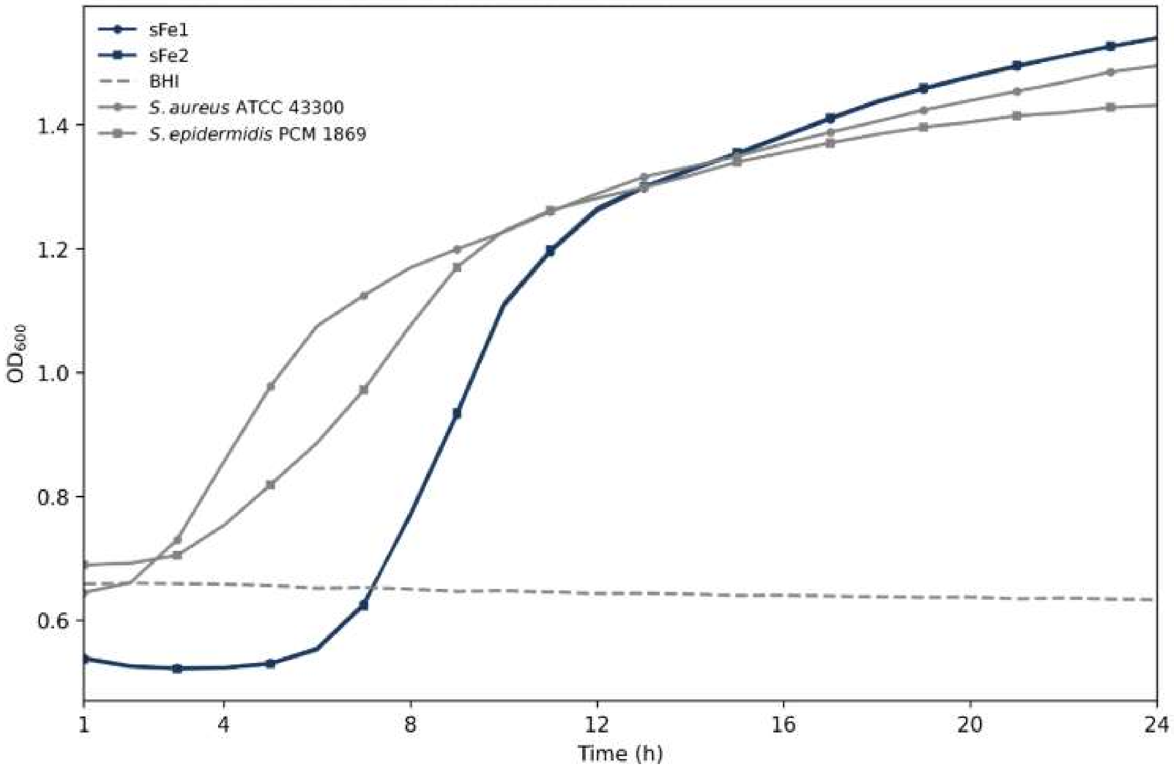

Figure S3n. Growth curves of all selected sFe strains in 38 ℃.

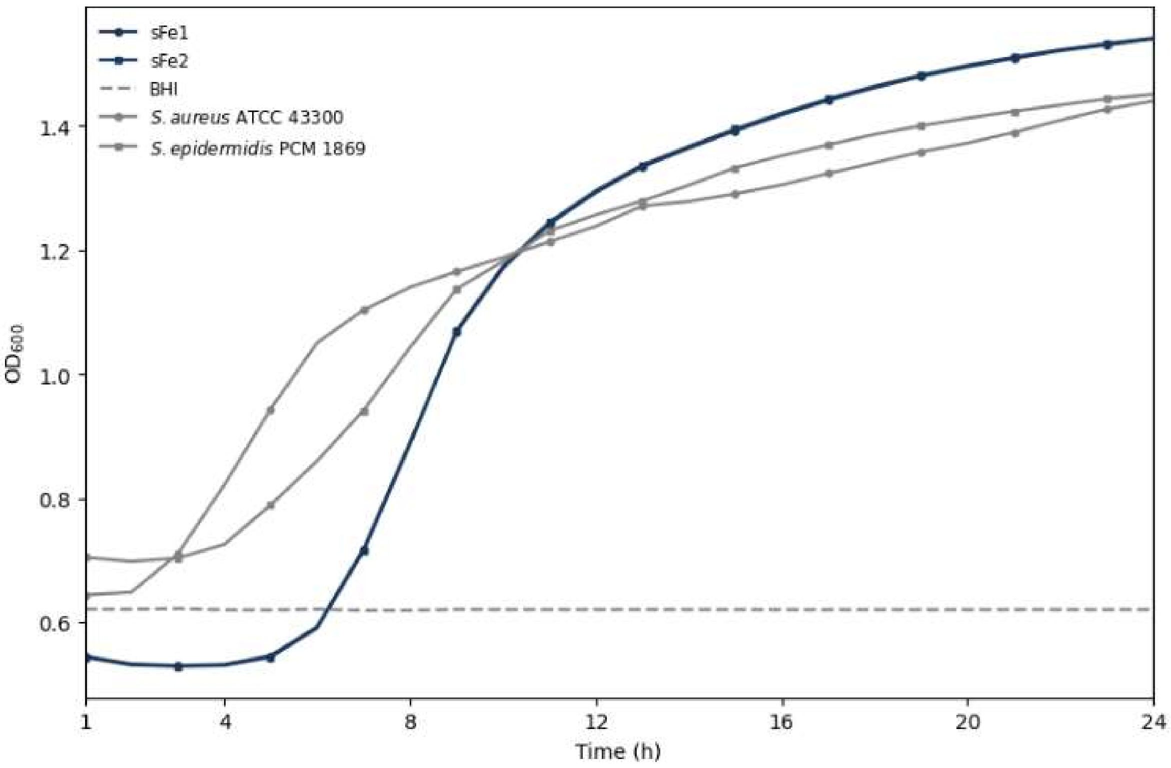

Figure S3o. Growth curves of all selected sFe strains in 39 °C.

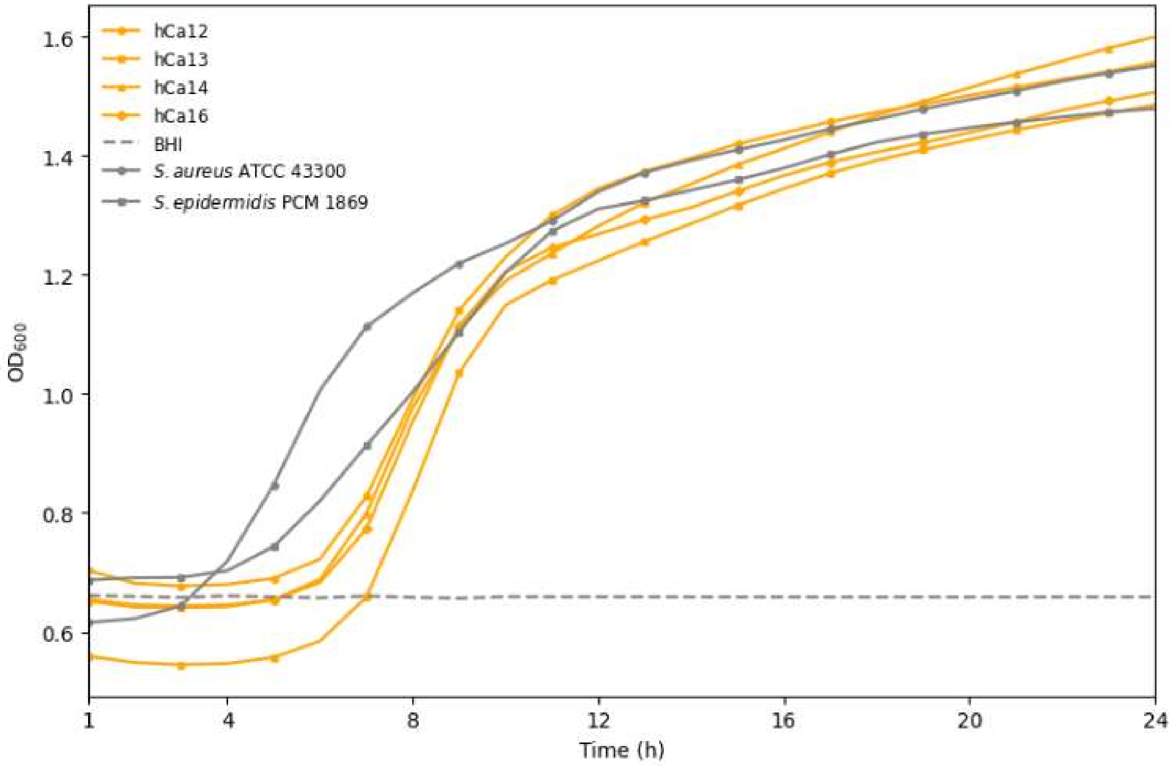

Figure S3p. Growth curves of all selected hCa strains in 37 °C.

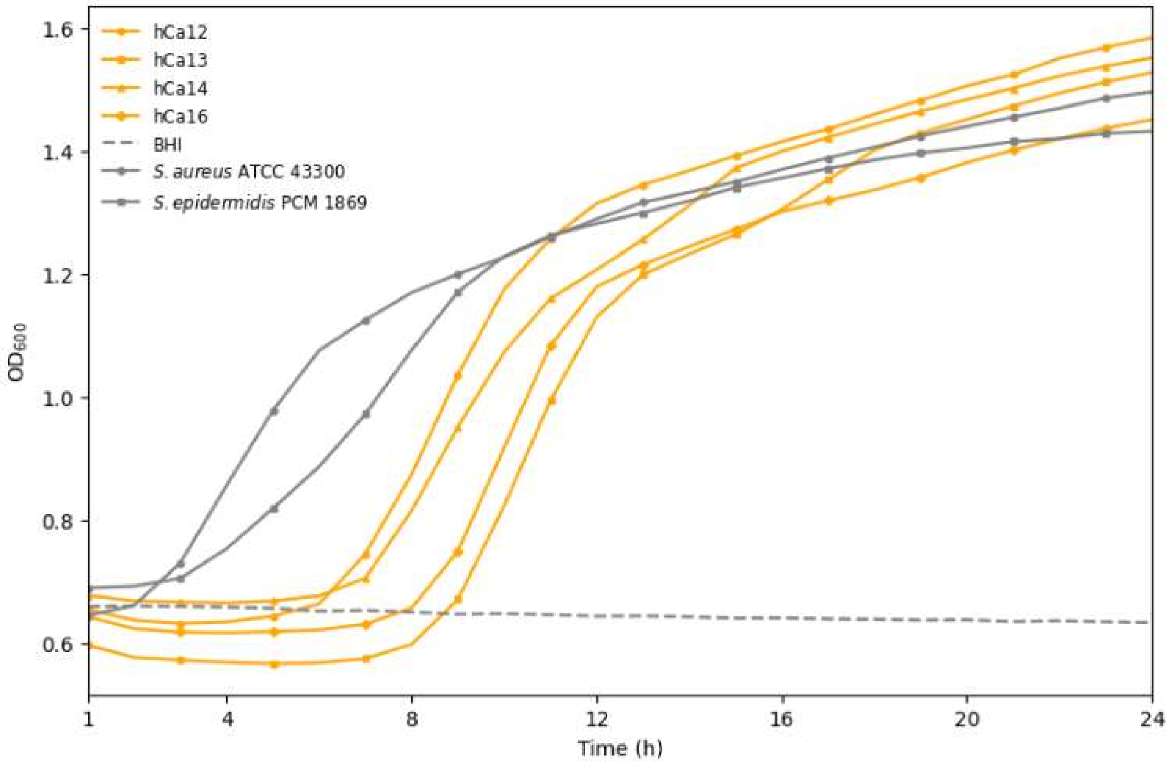

Figure S3q. Growth curves of all selected hCa strains in 38 ℃.

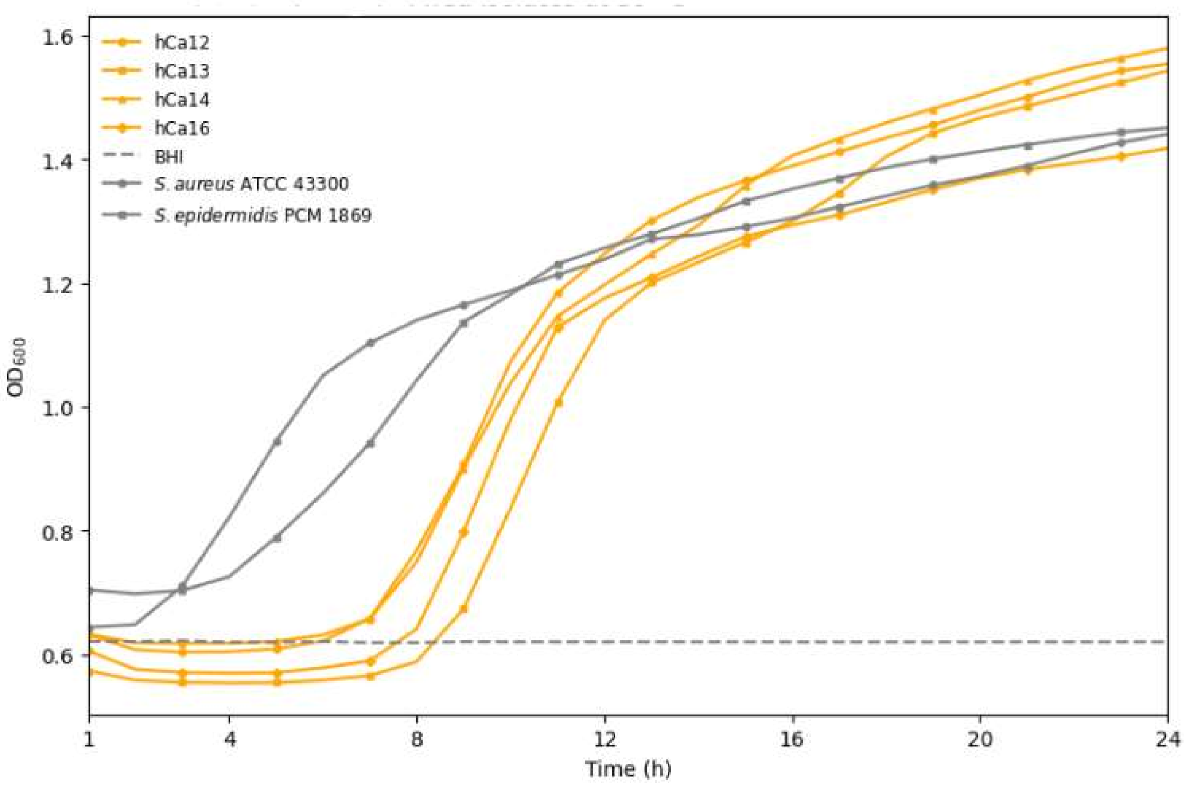

Figure S3r. Growth curves of all selected hCa strains in 39 ℃.

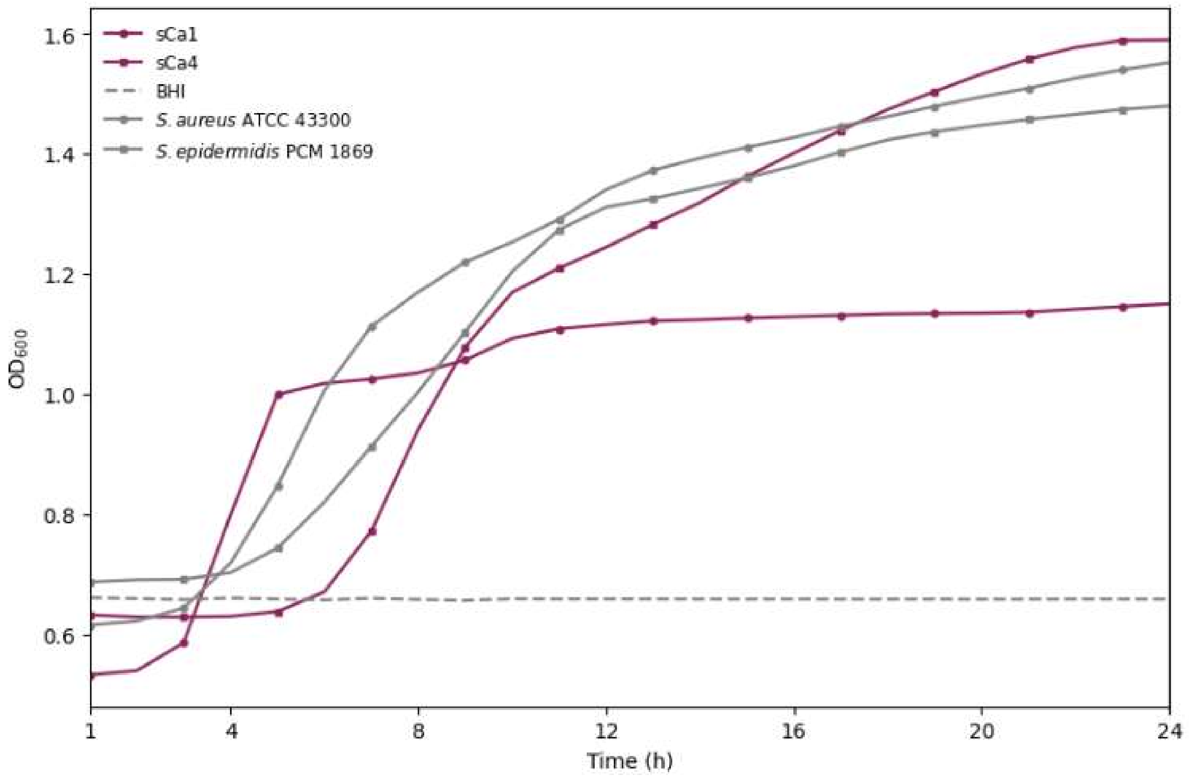

Figure S3s. Growth curves of all selected sCa strains in 37 ℃.

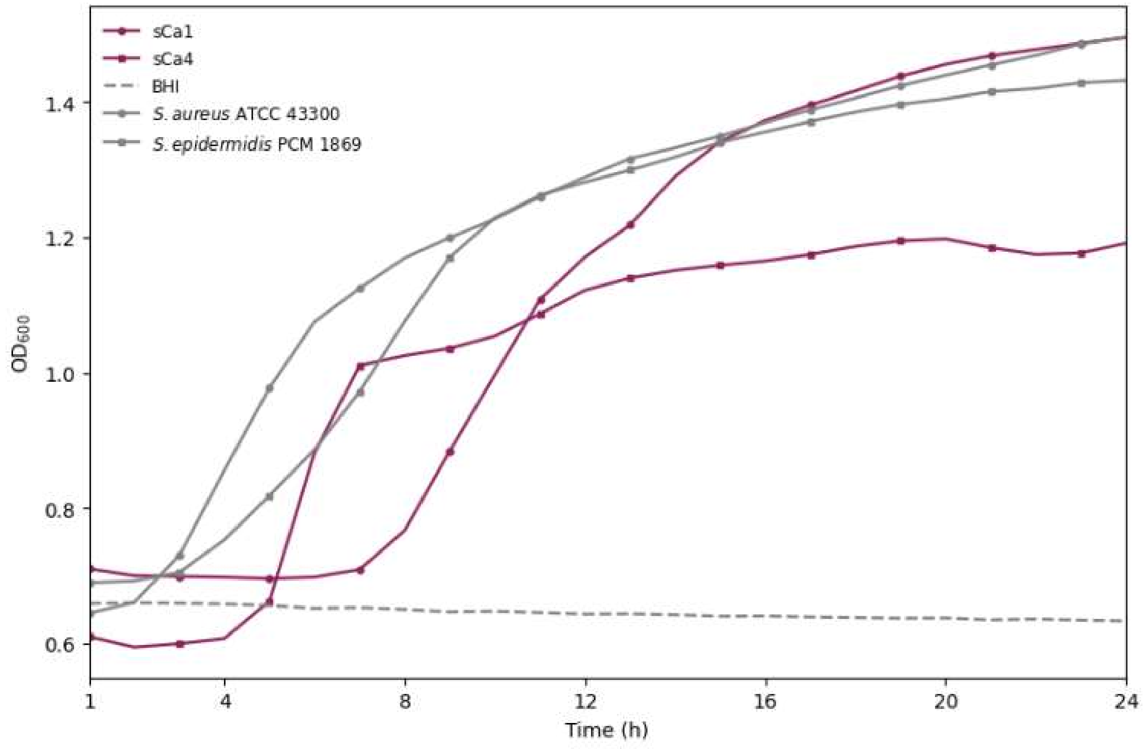

Figure S3t. Growth curves of all selected sCa strains in 38 ℃.

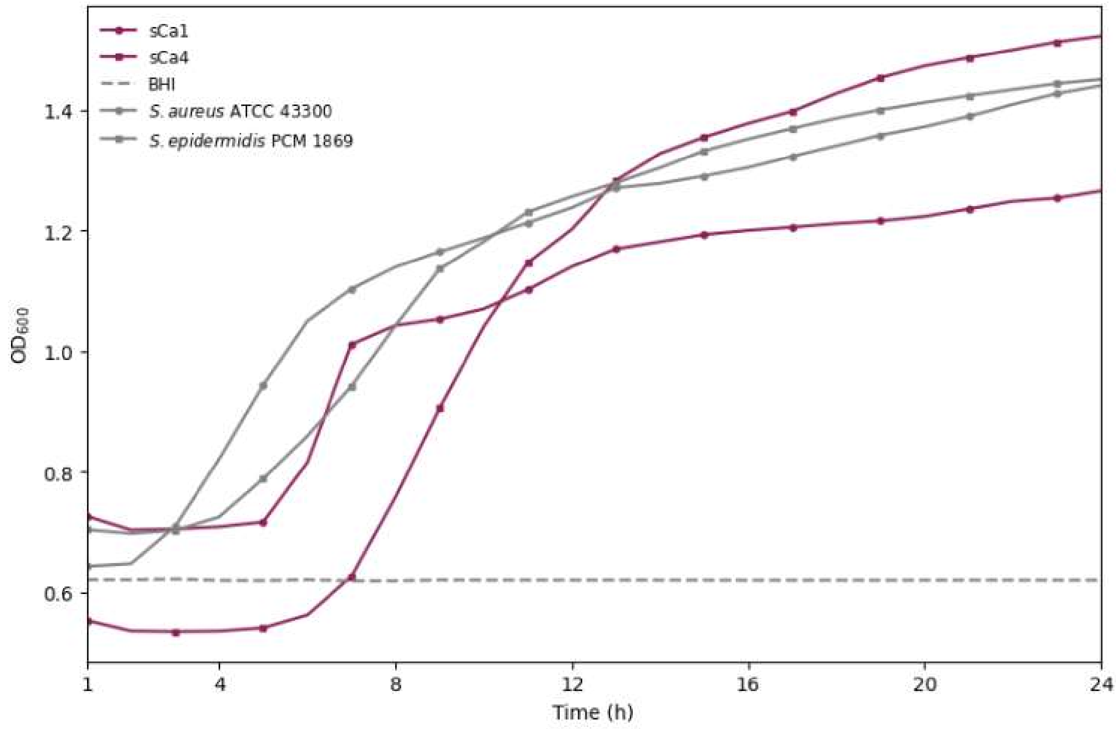

Figure S3u. Growth curves of all selected sCa strains in 39 ℃.

Figure S4. Doubling times of selected *Staphylococcus epidermidis* isolates and reference strains incubated at 37 °C, 38 °C, and 39 °C.

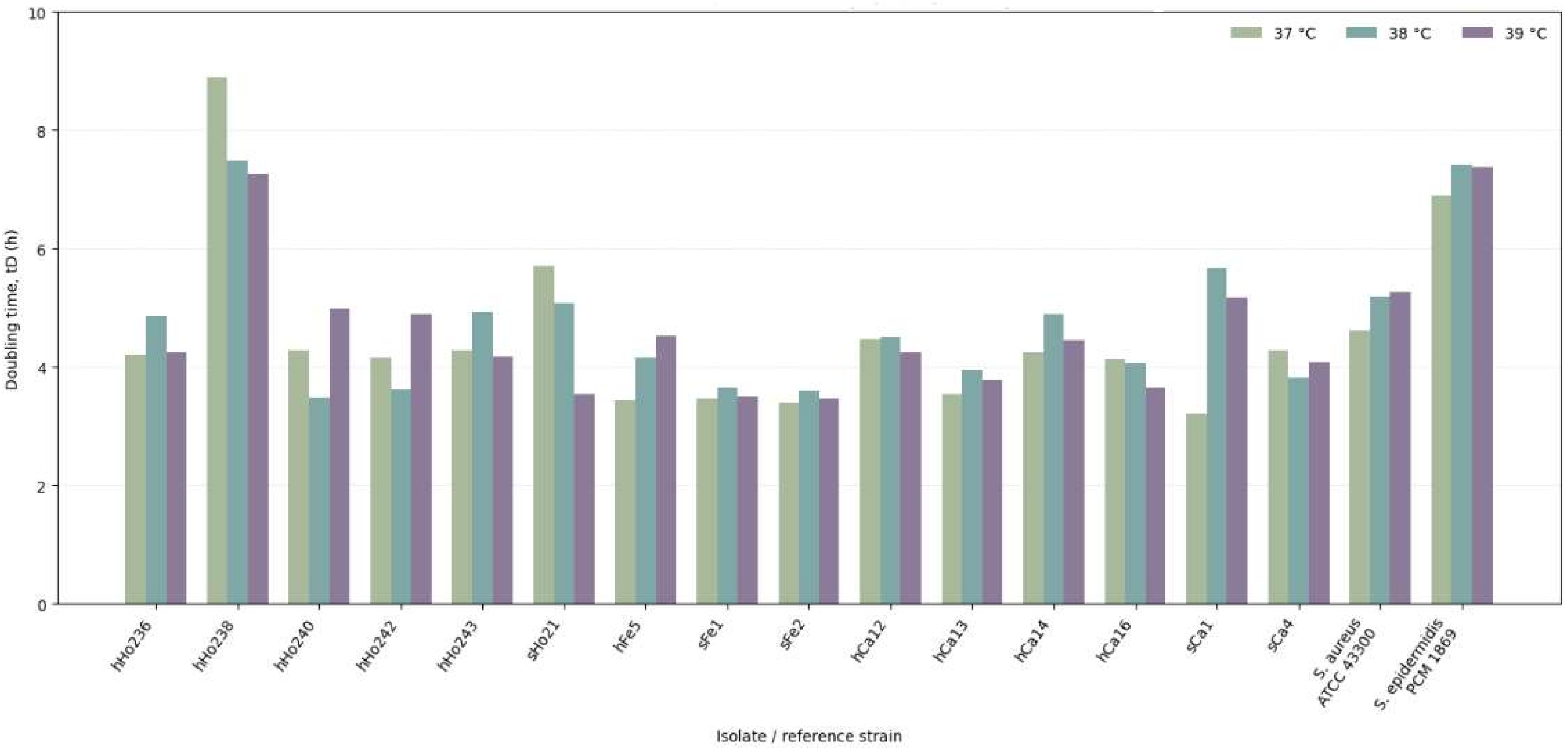

Table S12. Quantitative biofilm biomass (OD_{570}) of individual *Staphylococcus epidermidis* isolates after 24 h of incubation at 37 °C, 38 °C, and 39 °C.

	37	38	39
**sHo1**	2.4377	2.1215	1.0756
**sHo2**	2.4316	2.1588	2.3763
**sHo3**	2.4797	1.2420	0.5809
**sHo4**	1.6048	0.8903	0.9322
**sHo5**	1.2123	1.8710	1.3115
**sHo6**	3.0331	1.6034	3.6258
**sHo7**	2.7262	1.9189	1.5076
**sHo8**	1.8667	1.5596	1.6436
**sHo9**	3.800	3.7654	3.2345
**sHo10**	0.7878	0.5555	0.9988
**sHo11**	1.1305	1.1405	1.0931
**sHo12**	2.7336	3.1184	2.9014
**sHo13**	2.5666	3.0981	2.7654
**sHo14**	1.5530	2.6333	2.2722
**sHo15**	3.2028	3.1569	3.0979
**sHo16**	2.5860	2.8702	2.4102
**sHo17**	2.5677	1.9876	2.0145
**sHo18**	2.9008	3.4601	2.9161
**sHo19**	0.2987	0.4363	0.6819
**sHo20**	0.1582	0.2293	0.4056
**sHo21**	0.8555	0.3904	0.7168
**hHo1**	1.8901	1.0537	0.9214
**hHo2**	2.3760	2.5058	1.9869
**hHo3**	1.8693	2.2088	2.3589
**hHo4**	1.7930	1.3678	0.8877
**hHo5**	2.4869	1.3607	1.7666
**hHo6**	1.9816	1.5529	1.1458
**hHo7**	2.0510	1.8661	1.7558
**hHo8**	3.2202	3.0905	1.8904
**hHo9**	3.2184	2.5064	2.2339
**hHo10**	1.6989	3.7950	3.2571
**hHo11**	1.3246	1.1234	1.0123
**hHo12**	2.4924	1.2294	2.4183
**hHo13**	1.4752	1.5047	1.2533
**hHo14**	1.4464	0.7017	0.6036
**hHo15**	2.4763	1.6510	1.3819
**hHo16**	1.8235	2.7475	1.8767
**hHo17**	1.5893	3.6228	2.1209
**hHo18**	3.900	3.800	2.6012
**hHo19**	1.9022	1.0369	0.8379

**hHo20**	2.4561	1.7773	1.8798
**hHo21**	2.8267	1.1792	1.6017
**hHo22**	2.2212	1.3114	1.2389
**hHo23**	1.6827	1.5211	1.7027
**hHo24**	1.0868	0.8321	0.8676
**hHo25**	1.7232	0.6783	0.9670
**hHo26**	2.4078	1.5845	1.9621
**hHo27**	2.2931	2.2673	2.1461
**hHo28**	1.6619	0.8474	0.5802
**hHo29**	2.4200	1.6356	1.4274
**hHo30**	2.3846	1.3317	1.4764
**hHo31**	2.2484	1.4123	1.6884
**hHo32**	2.2353	3.0957	1.9544
**hHo33**	2.6233	1.9083	0.8947
**hHo34**	2.0823	1.2275	1.1267
**hHo35**	3.0656	1.6584	2.1015
**hHo36**	1.8276	2.1358	1.5072
**hHo37**	2.0687	3.900	1.6256
**hHo38**	3.3417	1.5054	1.0458
**hHo39**	3.0819	2.8424	1.7105
**hHo40**	1.5536	1.6773	0.5322
**hHo41**	1.9848	1.5371	1.1428
**hHo42**	1.3827	2.3261	1.3516
**hHo43**	3.5107	2.4634	0.7457
**hHo44**	2.1194	1.3016	0.8274
**hHo45**	1.7452	2.7310	2.3988
**hHo46**	0.9855	1.0538	1.0560
**hHo47**	1.6693	1.4934	1.5988
**hHo48**	2.1816	1.5874	1.2778
**hHo49**	2.1936	1.3706	1.2545
**hHo50**	1.9202	1.5019	2.3039
**hHo51**	2.7664	0.7836	0.4359
**hHo52**	2.7306	2.5004	0.7471
**hHo53**	3.9221	2.1072	1.8961
**hHo54**	2.4539	1.1067	0.9999
**hHo55**	2.3754	1.8247	1.4877
**hHo56**	2.6909	2.2514	0.9015
**hHo57**	1.1815	1.2239	0.9929
**hHo58**	1.3854	1.6891	0.8481
**hHo59**	1.4555	1.4665	1.9104
**hHo60**	1.8480	1.9004	0.8186
**hHo61**	3.0828	3.1796	3.3187
**hHo62**	1.6280	1.5359	1.0899

**hHo63**	1.0330	1.0034	0.8109
**hHo64**	3.0679	2.4695	0.9725
**hHo65**	3.2084	2.7197	2.2598
**hHo66**	1.0210	0.8627	0.5615
**hHo67**	1.4208	1.7389	1.3697
**hHo68**	1.8024	1.5761	0.7225
**hHo69**	2.1233	2.3730	1.7695
**hHo70**	2.2030	1.6334	1.0123
**hHo71**	2.7387	2.0168	0.8286
**hHo72**	2.2024	1.5783	0.8392
**hHo73**	3.4414	2.8561	2.6542
**hHo74**	2.6182	1.2719	0.7368
**hHo75**	4.5	3.8	3.7
**hHo76**	1.0471	0.9737	0.6842
**hHo77**	1.1308	1.9363	1.9436
**hHo78**	0.5757	0.5050	0.7878
**hHo79**	0.6826	2.0210	1.1627
**hHo80**	1.0137	0.7577	1.0422
**hHo81**	2.1889	1.2029	0.9302
**hHo82**	4.500	3.8415	3.800
**hHo83**	1.2026	0.7567	0.8718
**hHo84**	1.0645	1.0661	0.8836
**hHo85**	2.1605	1.4024	1.0983
**hHo86**	2.8710	3.2928	2.0591
**hHo87**	2.7412	3.1256	2.1956
**hHo88**	0.9601	0.7878	0.7522
**hHo89**	1.6853	0.9651	0.9780
**hHo90**	1.8429	1.1900	0.9042
**hHo91**	0.9667	0.6344	1.0536
**hHo92**	0.7724	1.0332	0.6681
**hHo93**	1.1053	0.8874	0.7592
**hHo94**	3.3433	2.5499	1.7840
**hHo95**	3.5969	3.3272	2.9094
**hHo96**	4.000	3.7958	3.4023
**hHo97**	3.500	3.1516	2.0111
**hHo98**	0.7984	1.1802	1.6111
**hHo99**	0.9332	1.5157	1.3871
**hHo100**	1.0532	1.0323	1,0036
**hHo101**	0.9670	1.0825	0.8784
**hHo102**	1.5462	1.9308	1.7437
**hHo103**	1.3516	1.9109	1.1427
**hHo104**	1.2338	1.5865	1.4567
**hHo105**	4.5	4.0	3.8

**hHo106**	0.8842	1.4392	1.1540
**hHo107**	0.9855	1.7138	1.1524
**hHo108**	3.0414	1.6467	1.9651
**hHo109**	4.500	4.000	3.800
**hHo110**	2.9785	3.2011	2.0158
**hHo111**	2.3275	2.2637	3.3985
**hHo112**	1.3459	2.1416	1.3538
**hHo113**	2.0618	0.8461	1.3866
**hHo114**	3.5958	2.4769	2.0745
**hHo115**	2.1222	1.3212	0.9889
**hHo116**	2.5555	3.1021	2.1020
**hHo117**	2.6158	3.2024	2.0125
**hHo118**	2.2691	1.4996	1.1174
**hHo119**	3.8000	3.7890	3.4566
**hHo120**	1.7357	1.8626	1.8271
**hHo121**	0.6094	0.3953	0.8490
**hHo122**	2.7323	3.1002	2.2000
**hHo123**	2.0691	1.4317	0.8176
**hHo124**	2.1956	1.4233	0.8123
**hHo125**	1.0551	1.4845	1.6377
**hHo126**	1.6353	1.7829	1.5140
**hHo127**	0.6679	1.3567	1.0424
**hHo128**	0.7772	0.8082	1.0137
**hHo129**	1.3234	1.5335	1.5366
**hHo130**	0.6583	1.3733	1.1117
**hHo131**	1.5169	1.6340	1.5355
**hHo132**	2.2603	2.6688	2.3817
**hHo133**	1.3096	0.9971	1.7379
**hHo134**	1.7429	2.5055	1.6868
**hHo135**	1.8554	1.2134	0.5081
**hHo136**	1.1536	1.0106	1.0298
**hHo137**	1.8413	1.4171	0.9671
**hHo138**	1.8080	1.2428	0.7748
**hHo139**	0.7745	0.5508	0.9531
**hHo140**	1.3447	1.1047	1.8351
**hHo141**	1.9880	1.6645	1.2425
**hHo142**	0.4155	0.3318	0.4769
**hHo143**	4.000	3.0973	3.8234
**hHo144**	1.5437	1.0899	0.9410
**hHo145**	2.5049	2.8406	2.4978
**hHo146**	1.1345	0.8765	0.5674
**hHo147**	0.5800	0.5068	0.7857
**hHo148**	0.5800	0.5068	0.7857

**hHo149**	0.8497	0.5097	1.0730
**hHo150**	2.3187	2.1741	1.6115
**hHo151**	1.5154	1.8470	2.1628
**hHo152**	0.4467	0.2469	0.2762
**hHo153**	0.1926	0.3238	0.3801
**hHo154**	0.0719	0.1365	0.0778
**hHo155**	0.1258	0.2228	0.3247
**hHo156**	0.2608	0.5950	0.6946
**hHo157**	0.1733	0.2672	0.5561
**hHo158**	0.1005	0.3063	0.1706
**hHo159**	0.1631	0.2670	0.4841
**hHo160**	0.6528	0.3224	1.2195
**hHo161**	0.6661	0.3344	1.3233
**hHo162**	3.1718	2.0108	2.9926
**hHo163**	1.3267	2.2055	0.1302
**hHo164**	0.0910	0.2079	0.1914
**hHo165**	0.0791	0.1110	0.0968
**hHo166**	0.0886	0.2627	0.2666
**hHo167**	0.1045	0.1491	0.0939
**hHo168**	0.3271	0.5237	0.5397
**hHo169**	0.1152	0.1581	0.1454
**hHo170**	0.0898	0.2137	0.1322
**hHo171**	0.7219	0.2576	0.5050
**hHo172**	0.7935	0.2521	0.5309
**hHo173**	1.1518	1.5036	1.7466
**hHo174**	0.1858	0.2305	0.1407
**hHo175**	0.8742	0.9571	0.7126
**hHo176**	0.5891	0.2791	0.5886
**hHo177**	0.3905	0.3928	0.8846
**hHo178**	3.5828	2.5815	3.2477
**hHo179**	0.2308	0.2110	0.1143
**hHo180**	2.3733	3.5206	2.8304
**hHo181**	0.9587	0.1974	0.2478
**hHo182**	0.0922	0.0859	0.0919
**hHo183**	0.0933	0.0881	0.0901
**hHo184**	1.1414	1.3603	0.4474
**hHo185**	3.6188	3.4146	0.1089
**hHo186**	0.0869	0.4157	0.2441
**hHo187**	0.0841	0.1391	0.1255
**hHo188**	0.3333	0.9856	0.1795
**hHo189**	0.4350	0.3078	0.3083
**hHo190**	0.2741	0.1848	0.4566
**hHo191**	0.1494	0.3071	0.1285

**hHo192**	0.1005	0.1274	0.1038
**hHo193**	0.4444	0.4000	0.3700
**hHo194**	0.1496	0.4103	0.2293
**hHo195**	0.3836	0.5559	0.2740
**hHo196**	2.0883	1.1667	2.5078
**hHo197**	0.2859	0.2756	0.6056
**hHo198**	0.1925	0.3467	0.1164
**hHo199**	0.4245	0.3670	0.0893
**hHo200**	0.3716	0.2841	0.3967
**hHo201**	1.7633	1.0278	1.0949
**hHo202**	0.0979	0.1716	0.1050
**hHo203**	0.4979	0.5436	0.3999
**hHo204**	0.0866	0.0882	0.0902
**hHo205**	0.3991	0.5346	0.7381
**hHo206**	0.3496	0.3078	0.5253
**hHo207**	0.4776	0.4045	0.3975
**hHo208**	0.9871	0.3394	0.1652
**hHo209**	0.3547	0.2879	0.0926
**hHo210**	0.1096	0.4617	0.2308
**hHo211**	0.0970	0.1747	0.1270
**hHo212**	0.3007	0.9360	0.2984
**hHo213**	0.4626	0.8229	0.2465
**hHo214**	0.4046	0.5132	0.6204
**hHo215**	0.7288	0.6137	0.8268
**hHo216**	0.3464	0.4187	0.5423
**hHo217**	0.5609	0.2077	0.4157
**hHo218**	0.4165	0.2218	0.4250
**hHo219**	0.8539	0.5969	0.5722
**hHo220**	0.1547	1.1580	0.1445
**hHo221**	0.4980	0.7212	0.1829
**hHo222**	0.1428	0.5671	0.2579
**hHo223**	0.2845	0.6453	0.1195
**hHo224**	0.1071	0.3778	0.1793
**hHo225**	0.1035	0.1180	0.0952
**hHo226**	0.1623	0.2772	0.2201
**hHo227**	0.1093	0.2212	0.2113
**hHo228**	0.1155	0.3748	0.7204
**hHo229**	0.1488	0.2162	0.3501
**hHo230**	0.1259	0.1595	0.0923
**hHo231**	0.2290	0.8314	0.1981
**hHo232**	0.6777	0.3950	0.3898
**hHo233**	0.6037	0.6389	0.3239
**hHo234**	0.5306	0.2886	0.5258

**hHo235**	0.7024	0.4035	0.4301
**hHo236**	0.5634	0.4709	0.3756
**hHo237**	0.7326	0.8127	0.3120
**hHo238**	1.0961	1.2658	0.9507
**hHo239**	1.0190	0.5061	0.4494
**hHo240**	0.3913	0.2828	0.3253
**hHo241**	0.9692	0.4348	0.5103
**hHo242**	0.6916	0.6840	0.2455
**hHo243**	0.8142	0.3623	0.3220
**hFe1**	0.7471	0.3487	0.3111
**hFe2**	0.5331	0.3350	0.4005
**hFe3**	0.6094	0.4225	0.3300
**hFe4**	0.4504	0.3660	0.2101
**hFe5**	0.6376	0.4202	0.2542
**hFe6**	0.4842	0.2408	0.3542
**hFe7**	1.1546	0.3303	0.3904
**hFe8**	0.4625	0.3073	0.3616
**hFe9**	0.7007	0.3093	0.3138
**hFe10**	0.5208	0.2744	0.2956
**hFe11**	2.6009	0.6623	0.7843
**hFe12**	0.6231	0.3337	0.3837
**hFe13**	0.5017	0.4025	0.5478
**hFe14**	0.5458	0.4282	0.2673
**hFe15**	0.7162	0.7867	0.3839
**hFe16**	0.4248	0.2325	0.3223
**hFe17**	0.7152	0.3178	0.5490
**hFe18**	0.7425	0.3458	0.5558
**hFe19**	0.7688	0.3972	0.2532
**hFe20**	0.5804	0.4197	0.3398
**hFe21**	4,2	3.6580	3.5785
**hFe22**	0.4545	0.3002	0.2760
**hFe23**	0.7498	0.3283	0.3213
**hFe24**	0.5773	0.4428	0.3506
**hFe25**	0.5894	0.3402	0.3415
**hFe26**	0.3919	0.3826	0.3581
**hFe27**	0.5548	0.2629	0.2822
**hFe28**	0.9864	0.4113	0.3318
**hFe29**	0.8463	0.2991	0.3083
**hFe30**	0.8867	0.4486	0.3416
**hFe31**	0.8913	0.2534	0.3957
**hFe32**	0.6784	0.6238	0.2633
**sFe1**	0.6252	0.2397	0.4232
**sFe2**	0.7600	0.3024	0.3601

**hCa1**	3.8438	2.3568	2.5826
**hCa2**	0.5000	0.4212	0.3044
**hCa3**	0.5003	0.3778	0.3040
**hCa4**	0.7080	0.3644	0.3341
**hCa5**	0.5608	0.2267	0.3636
**hCa6**	3.4410	3.6668	3.800
**hCa7**	0.4652	0.2565	0.3632
**hCa8**	0.4221	0.2321	0.3322
**hCa9**	1.9607	0.6825	0.5171
**hCa10**	0.9445	0.7584	0.5368
**hCa11**	0.4829	0.2687	0.3421
**hCa12**	0.5547	0.2888	0.2171
**hCa13**	0.2861	0.3035	0.2419
**hCa14**	0.7205	0.4333	0.3479
**hCa15**	0.5696	0.3169	0.2558
**hCa16**	0.4854	0.5295	0.2473
**hCa17**	0.3852	0.4952	0.3212
**hCa18**	0.4021	0.2420	0.3326
**sCa1**	0.3831	0.2552	0.2539
**sCa2**	0.6875	0.3167	0.3679
**sCa3**	0.5458	0.2599	0.3499
**sCa4**	1.0268	0.6176	0.3552

Biofilm-forming capacity was classified as follows: non-producer, $OD_{570} \leq 0.2122$; weak producer, $0.2122 < OD_{570} \leq 0.4244$; moderate producer, $0.4244 < OD_{570} \leq 0.8488$; strong producer, $OD_{570} > 0.8488$. The “non-producer” category denotes OD_{570} values at or below the predefined ODc threshold and does not imply a complete absence of biofilm-forming capacity.

Table S13. Antimicrobial susceptibility testing criteria, including disc contents, interpretive thresholds, bacterial groups, and reference standards.

Antimicrobial agent	Abbreviation	Disk content	Interpretive criterion	Source / basis	Comment
Amoxicillin/clavulanic acid	AMC	20/10 µg	**S ≥20 mm; R ≤19 mm**	**[52]**	Historical CLSI breakpoint for *Staphylococcus spp.*; archived by CLSI.
Ampicillin	AMP	10 µg	**S ≥29 mm; R ≤28 mm**	**[53]**	Historical CLSI breakpoint for *Staphylococcus spp.*
Penicillin G	P	10 U	**S ≥29 mm; R ≤28 mm**	**[50]**	Current CLSI criterion for all staphylococci; β-lactamase/*blaZ* should be considered in apparently susceptible isolates.
Oxacillin	OX	**1 µg**	**S ≥18 mm; R ≤17 mm**	**[50]**	Species-specific disk-diffusion criterion for *S. epidermidis*
Mupirocin	MUP	200 µg	**S ≥30 mm; I 18–29 mm; R <18 mm**	**[55]**	*S. aureus* criterion used as surrogate for *S. epidermidis*. EUCAST specifies ≥30 mm for susceptibility and <18 mm for resistance in nasal decolonization.
Marbofloxacin	MAR	5 µg	**S ≥23 mm; I 16–22 mm; R ≤16 mm**	**[51]**	Veterinary criterion for *Staphylococcus spp.*; current CLSI materials retain this interpretation.
Ciprofloxacin	CIP	5 µg	**S ≥21 mm; I 16–20 mm; R ≤15 mm**	**[50]**	Current CLSI criterion for staphylococci.
Linezolid	LZD	30 µg	**S ≥26 mm; I 23–25 mm; R ≤22 mm**	**[50]**	Current CLSI criterion.
Tigecycline	TGC	15 µg	**S ≥26 mm; R ≤25 mm**	**[56]**	Published BSAC-derived disk-diffusion criterion for *Staphylococcus* spp.
Erythromycin	E	15 µg	**S ≥23 mm; I 14–22 mm; R ≤13 mm**	**[50]**	Current CLSI criterion for staphylococci.
Clindamycin	DA	2 µg	**S ≥21 mm; I 15–20 mm; R ≤14 mm**	**[50]**	Current CLSI criterion; inducible resistance should be considered where relevant.
Tetracycline	TET	30 µg	**S ≥19 mm; I 15–18 mm; R ≤14 mm**	**[50]**	Current CLSI criterion.
Tobramycin	TOB	10 µg	**S ≥15 mm; I 13–14 mm; R ≤12 mm**	**[54]**	Breakpoint archived after Ed27; no longer active in current M100.
Fusidic acid	FD	10 µg	**S ≥24 mm; R <24 mm**	**[55]**	Criterion listed for *Staphylococcus spp.*; no surrogate annotation.
Chloramphenicol	C	30 µg	**S ≥18 mm; I 13–17 mm; R ≤12 mm**	**[50]**	Current CLSI criterion.
Gentamicin	CN	10 µg	**S ≥15 mm; I 13–14 mm; R ≤12 mm**	**[50]**	Current CLSI criterion.
Trimethoprim/sulfamethoxazole	SXT	1.25/23.75 µg	**S ≥16 mm; I 11–15 mm; R ≤10 mm**	**[50]**	Current CLSI criterion.
Rifampicin	RD	5 µg	**S ≥20 mm; I 17–19 mm; R ≤16 mm**	**[50]**	Current CLSI criterion.
